# Assessing Sodium Amide Reagents for Ester Amidations in Deep Eutectic Solvents in Continuous Flow

**DOI:** 10.1021/acssuschemeng.5c08775

**Published:** 2025-10-31

**Authors:** Andrew W. J. Platten, Bruno Pinho, Laura Torrente-Murciano, Eva Hevia

**Affiliations:** † Department für Chemie Biochemie und Pharmazie, 27210 Universität Bern, Freiestrasse 3, 3012 Bern, Switzerland; ‡ Department of Chemical Engineering and Biotechnology, 2152 University of Cambridge, Philippa Fawcett Drive, Cambridge CB3 0AS, U.K.

**Keywords:** sodium amides, Deep Eutectic Solvents, continuous flow, solvent effects, sustainable organometallic reagents

## Abstract

Advancing the synthetic applications of sodium amides, this study pioneers their use in deep eutectic solvents (DES) for efficient amidation of a range of esters and for the C–F bond amination of difluoropyridine in continuous flow. These processes occur at room temperature and tolerate the presence of air and moisture, without the requirement for strictly dried organic solvents, conditions typically disallowed in sodium amide chemistry. Reactivity studies demonstrate that the DES plays a key role by facilitating the formation of a unique biphasic system with the organic solvents employed, which can operate under segmented flow in a coiled reactor. These conditions allow access to a wide range of amides with higher conversions and selectivities than those when working in conventional batch conditions, while working under quasi-stoichiometric conditions. Notably, the sodium salts (NaOR, NaF, etc.) formed as byproducts from these reactions can be finely dispersed through the DES, preventing clogging of the flow reactor. To address the limited availability of commercially viable sodium amide reagents, we demonstrate their *in situ* flow synthesis using NaN­(SiMe_3_)_2_ as a precursor. Combining X-ray crystallographic and spectroscopic studies, a closer look into the constitution of these powerful amidation reagents is provided, uncovering their tendency to form kinetically activated monomeric/dimeric species in THF solutions.

## Graphical abstract

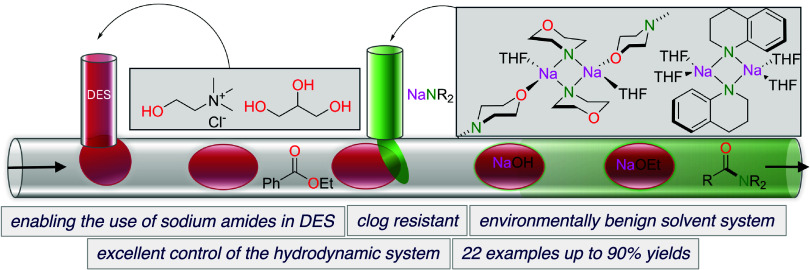

## Introduction

In contrast with the widespread applications of organolithium and lithium amide reagents,^1–3^ amides and alkyl compounds of sodium have made a limited impact in organic chemistry. This situation is in part due to a combination of operational challenges in their manipulation associated with their low solubility in organic solvents, their extreme air and moisture sensitivity, the limited commercial availability of their precursors, and their untamed reactivity, which in many cases compromises their selectivity.^4^ However, the relatively higher crustal abundance of sodium (2.6% mass) compared to that of lithium (0.002% mass)^5^ has sparked renewed interest in the use of organosodium and sodium amide complexes. Recent studies highlighting the unique reactivity profiles of these reagents have further contributed to this resurgence.^4,6^ Takai et al. showcased the synthesis of sodium aryls^7^ and sodium amides^8^ from sodium dispersions, triggering significant exploration into their uses in synthesis for the formation of C–C bonds, which exploits their strong nucleophilic power. Using an alternative strategy, Knochel reported *in situ* synthesis of organosodium reagents in a flow system using sodium-packed columns and hexane, along with amine donors as a solvent system, where products containing unreacted reagents were subsequently quenched with assorted electrophiles under batch conditions (Figure 1a).^9,10^ These flow approaches have also been extended to sodium amide metalations. Remarkably, the use of flow reactors is critical for the success of this approach, with the same reactions often failing in batch due to the fast decomposition of the highly sensitive organometallic intermediates.^11^ While all of these studies require the use of dry, degassed organic solvents, recent studies by Capriati have shown that organosodium reagents can also be used in deep eutectic solvents (DES). In this single report, the organosodium reagents are generated in organic solvents using Na bricks and the relevant alkyl or aryl chloride and then treated with an electrophile using a DES made up of choline chloride (ChCl) and urea as solvents (Figure 1b).^12^

**Figure 1:**
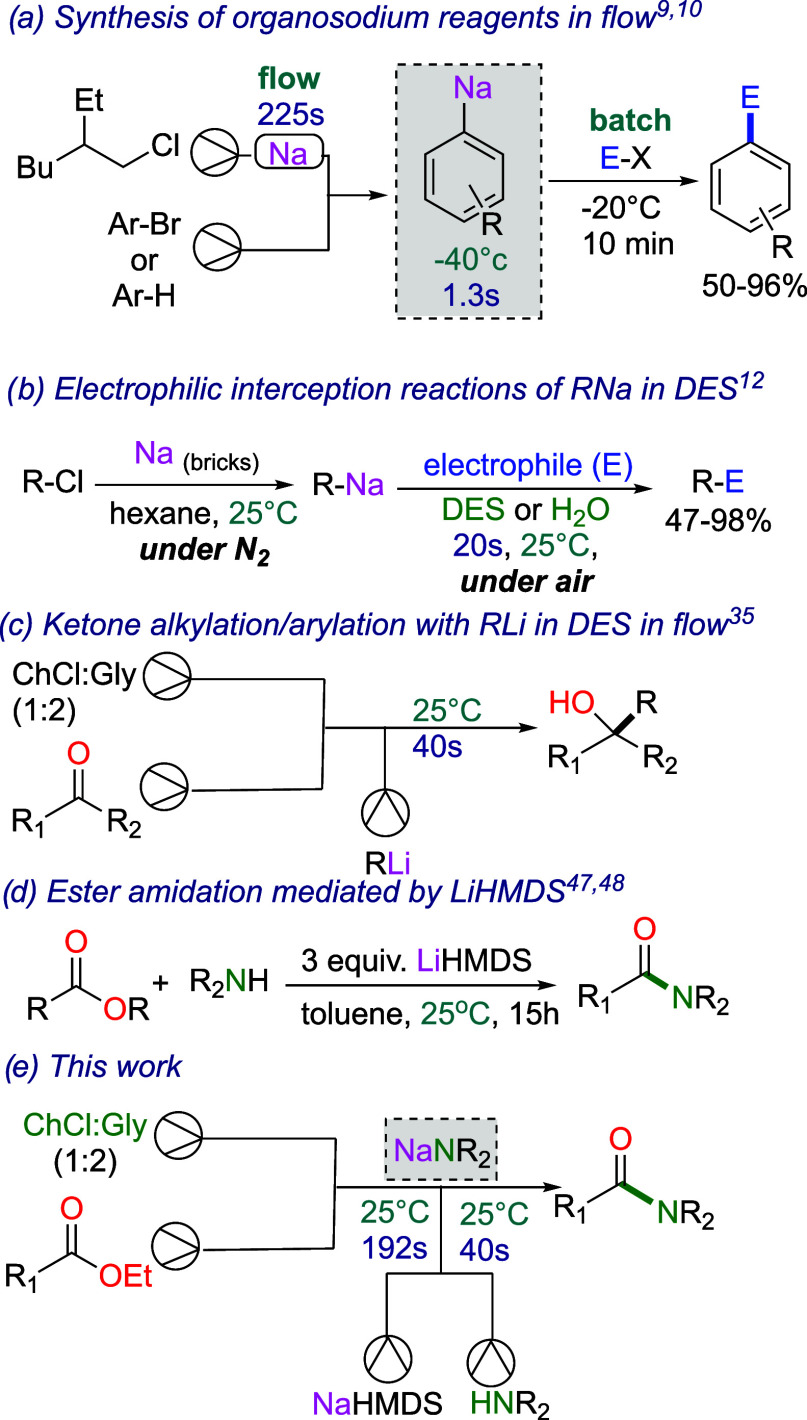
Selected examples of amidations and developments in organosodium chemistry. (a) *In situ* preparation of organosodium (flow) followed by metalation and quenching (batch) (Knochel et al.),^9,10^ (b) electrophilic interception reactions of organosodiums in DES (Capriati et al.),^12^ (c) ester amidations mediated by LiHMDS in toluene (Szostak et al.),^47,48^ (d) ketone alkylation/arylation using organolithiums in DES in continuous flow (Torrente et al),^35^ and (e) this work.

It should be noted that the use of DES as solvents generates widespread interest due to their ability to act as uniquely tunable green solvents,^13^ being safe and more environmentally benign alternatives to toxic organic solvents.^14–16^ Offering a diverse range of properties, DES solvents have been employed in a multitude of organic transformations under batch conditions, including reactions catalyzed by transition metal complexes^16–20^ and enzymes.^21–25^ Studies by the groups of Capriati, Garcia-Alvarez, Prandi, and Hevia have shown that DESs based on choline chloride (ChCl) in combination with glycerol (Gly), water, or ethylene glycol, among other H-bond donors, can be excellent reaction media for polar organometallic reagents such as organolithium and Grignard reagents.^26–34^

Due to the high basicity and moisture sensitivity of these reagents, it could be expected that they would be incompatible with these solvent combinations, yet these studies have demonstrated that, against conventional wisdom, these solvents can kinetically enhance their reactivity, leading to special chemoselectivities that cannot be achieved in conventional organic toxic solvents while working under aerobic conditions.^26–34^ Advancing the synthetic applicability of these protocols, our groups have recently shown that organolithium and Grignard reagents can be used in nucleophilic arylation and alkylation of ketones and imines using DES as a carrier solvent in continuous flow, with a high stability and tolerance to water.^35^ These studies have also revealed that the DES plays an important role to avoid clogging in the reactor under ambient conditions, favoring the formation of a two-phase system.

Inspired by these more sustainable innovations, we next pondered if amides containing earth-abundant sodium could also be used in DES under continuous flow conditions. Bearing in mind their enhanced reactivity in comparison with their lithium counterparts,^4,36,37,38^ their use under these conditions poses a greater challenge in terms of precluding their fast decomposition in the DES media but simultaneously opens up potential opportunities for unlocking new reactivities. Exploiting the expected enhanced nucleophilic power of sodium amides over their lithium analogues, here we explore their potential to promote ester amidation reactions to access organic amides. The synthesis of amides is of particular importance due to their versatile reactivity within the pharmaceutical and agrochemical industries.^39^ While many methodologies exist, new approaches for the synthesis of amides have been repeatedly identified as a key area of research that requires more development.^40,41^ The simplest methodology is the introduction of carboxylic acids to amines, which normally requires high temperatures and activated substrates to work effectively, with only a few uses in industry.^42,43^ While several alternative methods have been developed, including the preactivation of the carboxylic acid partner to promote coupling with the relevant amine under mild conditions, recent approaches have shifted toward activating the amine partner.^44–46^ This can be achieved by converting the amine into a more nucleophilic lithium amide, which can subsequently react with less activated substrates such as esters. Indeed, Szostak et al. have shown that LiHMDS (HMDS = 1,1,1,3,3,3-hexamethyldisilazide) can mediate amidation of esters with a wide range of amines at room temperature (Figure 1c).^47,48^ However, reactions need to be carried out in toluene under inert atmosphere conditions while using a wasteful excess of lithium amide and the relevant amine. Increasing the sustainability of this approach, our group has subsequently shown that using biorenewable and strongly coordinating 2-MeTHF, ester amidations can occur at ambient temperature under air with exceedingly short reaction times (up to 20 s).^49^ More recently, Collum has shown that sodium isopropyl­(trimethylsilyl)­amide can promote the amidation of methylbenzoate of a small selection of amines,^50^ where the choice of solvent or donor additive can greatly influence the rate of these reactions.

Taking the use of polar Group 1 metal reagents in DES into unexplored territory, in this work, applications of sodium amides in DES in continuous flow for the amidation of esters to access synthetically relevant organic amides in high yields with excellent selectivity are reported (Figure 1d). By optimizing the interactions of the biphasic DES system, along with the trapping and characterization of key important organometallic intermediates, new operational and mechanistic insights have been gained, which enable the extension of this approach for the transformation of challenging C–F bonds into C–N bonds using 2,6-difluoropyridine as a model substrate.

## Experimental Section

### Materials

NaHMDS (used as a 2 M solution in THF), organic substrates, and solvents were purchased from commercial suppliers and used as received. The following substrates were used in this study: **1a** Ethyl benzoate, **1b** ethyl 4-chlorobenzoate, **1c** ethyl 4-bromobenzoate,**1d** ethyl 3-iodobenzoate, **1e** ethyl 4-methoxybenzoate, **1f** ethyl furan-2-carboxylate,**1g** ethyl picolinate,**1h** ethyl 2-naphthoate, **1i** ethyl hexanoate, **1j**ethyl acetate, **1k** ethyl trifluoroacetate, **2** difluoropyridine, **3a** *N*-methylaniline, **3b** *N1,N1*-dimethylbenzene-1,4-diamine, **3c** 4-methoxyaniline, **3d** 3,4-(Methylenedioxy)­aniline, **3e** 2-iodoaniline, **3f** trimethylaniline, **3g** morpholine, **3h** tetrahydroquinoline

### Reactions in Flow, Microreactor Characteristics

The device was made of perfluoroalkoxy alkane (PFA) tubing (*D* = 1.01 mm for reactor 1 *D* = 0.76 mm for reactor 2 internal diameter), ethylene tetrafluoroethylene (ETFE)/PFA fittings, and four syringe pumps for delivering different fluids. A solution of the reactant in toluene was introduced via a T-mixer into a stream of the carrier phase (DES) to give a segmented flow regime with the droplets of the organic phase enveloped by the DES. Downstream, a commercial solution of NaHMDS (2 M) in THF was added using a second T-mixer. Subsequently, the addition reaction took place in a tubular microreactor (0.23 mL). The microreactor length (50 cm) allowed for an excess of residence time (on the order of 40 s) to ensure the full conversion of the amide. The outlet was led into a collection vial containing water to ensure the full hydrolysis of the hazardous unreacted organosodium species and to quench the reaction. After the reaction was complete, the reactor was flushed with water, followed by toluene prior to further use.

### Preparation of DES

All DES were synthesized according to literature precedent by taking the constituent components and heating in a vacuum oven. Storage of the DES in a vacuum oven was used to prevent high water content of the DES.

### Preparation of Reagent Solutions

Amines were measured out and dissolved in commercially available undried THF. Esters and difluoropyridine were dissolved in toluene along with hexamethylbenzene. Each of these solutions was added to 5 mL of their lock syringe to be mounted on the employed syringe pumps.

### Product Collection and Analysis

The reactor outlet was collected in a 10 mL glass vial as waste until the reactor was flushed 2.5 times based on its volume and the involved flow rates. Then, a 10 mL glass vial was used for collecting the solution from the reactor outlet. Hexamethylbenzene dissolved in the ester stock solution was used as an internal standard for the yield determination of the reactor output. Selectivity was determined based on the integral of remaining reactant, product, and (if any) side products.

## Results and Discussion

Investigating the potential for DES as a solvent for amidation reactions, ethyl benzoate **1a** was suspended in 1 g of DES ChCl:Gly (1:2) in an open-to-air batch reactor, to which a vigorously stirred 2 M solution of LiNMePh was added to form methyl-*N*-phenylbenzamide **4a** in a 60% yield (Table 1–amide screening). The results obtained proved encouraging, considering the highly air- and moisture-sensitive nature of lithium amides. Screening of various alkali-metal amides reveals a delicate balance: the alkali metal amides must be sufficiently reactive to facilitate the transformation within a short time frame, yet not so reactive that they decompose quickly through competing hydrolysis in the presence of the DES. The more electropositive nature of sodium enhances the reactivity of the N–Na bond in these compounds compared to LiNMePh, resulting in an increased yield of 77% (Table 1). In contrast, heavier alkali-metal amides such as KNMePh lead to a decreased yield, managing only 51% of **4a** (under the same conditions). This can be partly due to the reduced solubility of this metal amide in THF. By reducing the concentration of NaNMePh to 1M, near quantitative yields are obtained (94%) without the need of dry solvents or an oxygen-free atmosphere.

**Table 1:**
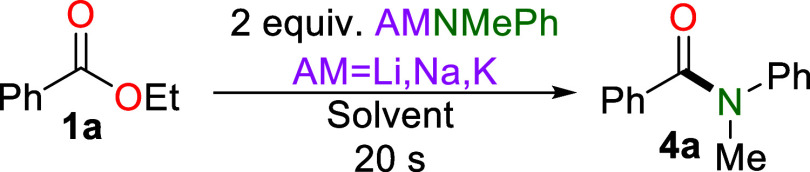
Optimization of Amidation of Ethyl Benzoate **1a** Using AMNMePh (AM= Li, Na) in a Batch Reactor^a^

amide	solvent	amide (M)	yield (%)
LiNMePh	ChCl:Gly (1:2)	2M	60
NaNMePh	ChCl:Gly (1:2)	2M	77
KNMePh	ChCl:Gly (1:2)	2M	51
NaNMePh	water	1M	10
NaNMePh	ChCl:Urea (1:2)	1M	63
NaNMePh	ChCl:Etgly (1:2)	1M	65
NaNMePh	ChCl:H_2_O (1:2)	1M	65
NaNMePh	ChCl:Gly (1:2)	1M	94
NaHMDS	ChCl:Gly (1:2)	1M	75

a Reactions performed on a 1 mmol scale by the addition of **AM**NMePh to **1a** in THF solution. 1 mmol in 1g of DES. Yields quantified by NMR against hexamethylbenzene as an average of two repetitions.

Other choline chloride-based deep eutectic solvents, such as the less viscous ChCl:EtGly (1:2), and ChCl:Urea (1:2) give yields of 65% and 63% respectively. While these yields are relatively high, they are still lower than that obtained with ChCl:Gly (94%: Table 1). Interestingly, choline chloride has been previously found to mediate coupling of carboxylic acids and amides although high temperatures (80–100 °C) are required.^43^ Despite organolithium and organosodium reagents being reactive enough to undergo addition reactions in water,^12,51^ water cannot facilitate the amidation reactions, with sodium amides yielding only 10% of **4a**. Impressively, in contrast, when the water-based DES ChCl:H_2_O (1:2) is used, the reaction yield jumps up to 65%. This demonstrates the competing effect of DES on subduing the hydrolysis reaction by forming complex hydrogen bonding networks.^35,52^

Unlike organolithium or Grignard reagents, hardly any sodium amides or organosodium reagents are commercially available due to their poor stability and limited solubility in organic solvents. In this case, the NaNMePh used in our initial studies (Table 1) was prepared under inert atmosphere conditions using *n*BuNa, which is highly sensitive and pyrophoric in the presence of air or moisture^53^ and insoluble in organic solvents. In order to facilitate its practical application, NaNMePh was prepared from commercially available NaHMDS [HMDS= N­(SiMe_3_)_2_] in THF under argon and reacted with **1a** on ChCl:Gly (1:2) DES, forming **4a** in similar yields of 90%. The under-air *in situ* formation of sodium amide **1a** was confirmed by adding a commercially available solution of NaHMDS to **1a** along with *N*-methylaniline in the presence of ChCl:Gly (1:2) DES. Achieving 75% of **4a** (Table 1) demonstrates that both amide formation and amidation reactions work in the presence of DES.

To better understand the effect of the alkali metal on the stability of the organometallic species, the order of addition was inverted, adding first the alkali metal amide (1M) to the ChCl:Gly (1:2) DES, followed by the ester after a set time (Figure 2).

**Figure 2:**
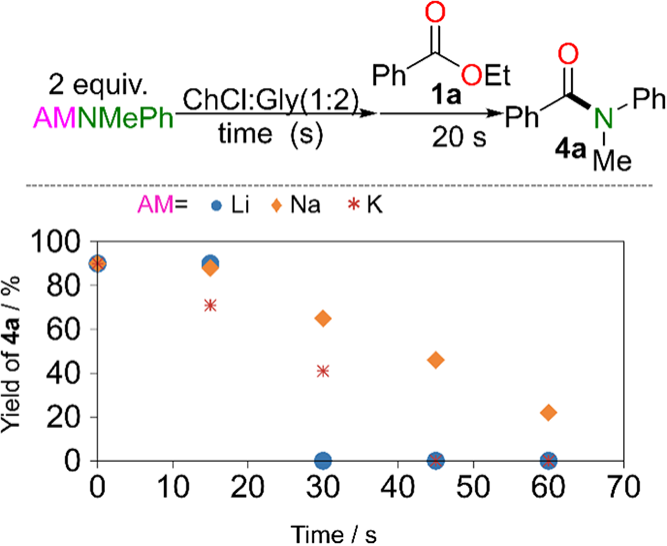
Comparison of (AM)­NMePh (where AM = Li, Na, K) for reverse order addition. (AM)­NMePh was first added to DES for a related time before the addition of ethyl benzoate, then further reaction for 20 s. Yields quantified by NMR against hexamethylbenzene as an internal standard were based on the average yield after two repetitions.

While initially LiNMePh still shows high reactivity when it is left to stir in the DES for 10 or 20 s, a dramatic drop is observed after 30 s, suppressing the formation of **4a**. Interestingly, when using KNMePh, which a priori can be expected to be more reactive than its lithium congener, and therefore decompose more rapidly in DES in the absence of **1a**, it permits the formation of **4a** in a 40% yield after 30 s. It is only after 45 s that no **4a** conversion is observed. In contrast, NaNMePh presents the optimum balance between reactivity and stability, with 20% of **4a** still being formed after 1 min. While these reactions are not a direct indication of the longevity of the alkali metal amide on DES, they do indicate that more reactive reagents, which may be viewed as poor reagents for in-air reactions, offer unexpected kinetic stability and high reactivity in these unconventional green solvents.

Reactions involving DES are biphasic in nature, requiring intimate contact between the DES and the organic solvent in which the organometallic reagent is dissolved.^35^ This contact is critical for the reaction’s success. Indeed, it has been shown that without stirring, such reactions offer diminished yields.^34,54–56^ Controlling and characterizing the degree of mixing in batch systems is normally challenging due to the multiple variables affecting it, such as volume of reactor, stirring rate, stirring system, and addition protocols. Therefore, a flow system is used herein as a repeatable, controllable, and robust alternative to a batch. The continuous platform consists of two reactors (Figure 3). In Reactor I, the relevant sodium amide is formed *in situ* by deprotonation of the amine at room temperature in THF using a commercially available solution of NaHMDS mixed in a 1:1 ratio with the amine. The residence time in Reactor I is 45 s, which was found to be sufficient for complete metalation of all amine substrates, demonstrating that high yields can be achieved within this short time frame. Prior to entering Reactor II, the amide-containing solution is mixed with a segmented flow consisting of the organic phase, containing the ester substrate in toluene, dispersed in the DES [ChCl:Gly (1:2)]. The flow ratio of the organic and DES inlet stream (*Q*_DES_:*Q*_**substrate**_) can be manipulated while keeping constant the *Q*_amide_:*Q*_substrate_ to **3**. In Reactor II, the amidation reaction takes place at room temperature, with a constant amide to substrate stoichiometric ratio of 1.25 and a constant flow rate of 1 mL/min. This results in a residence time of ∼40 s inside Reactor II.

**Figure 3:**
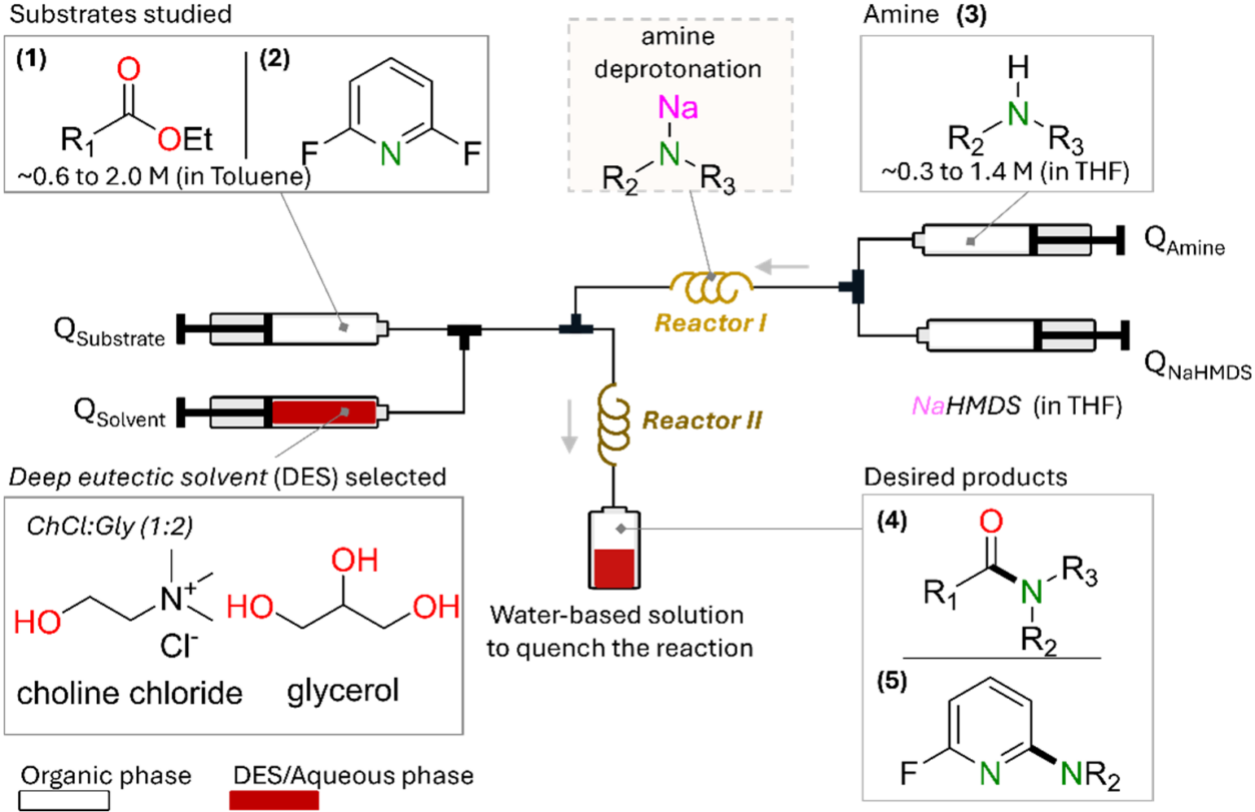
Schematic of the continuous flow reactor system consisting of two reactors (reactors I and II). In Reactor I, the amine deprotonation takes place at room temperature in THF for the *in situ* formation of sodium amide. The outlet of Reactor I is mixed with a segmented flow consisting of the organic phase (*e.g*., the ester substrate in toluene) dispersed in ChCl:Gly (1:2) DES. In Reactor II, the amidation reaction (or nucleophilic aromatic substitution) runs at room temperature with a constant amide to substrate ratio of 1.25, flow rate of 1 mL/min, and residence time of ∼40 s.

Mixing of substrate and amide organic phases in flow Reactor I was optimized to achieve high amide yields (>85%) by amide deprotonation. For this, clogging (due to water content in THF) was avoided by using a large enough reactor diameter (inner diameter 0.095 in) while increasing the flow rate to enhance mixing in the coiled reactor configuration (with a helical radius of 1 cm). Prior to entering the amidation Reactor II (inner diameter 0.03 in, and helical diameter of 1 cm), the outlet of Reactor I is mixed with the segmented flow consisting of a substrate-containing organic phase dispersed in DES (using a T-mixer). Two scenarios can happen: (i) the amide-containing THF solution is introduced in the substrate-containing organic segment with the amidation reaction taking place, yielding **4a** along with NaOEt or (ii) the amide-containing THF solution is introduced in the DES segment, leading to its hydrolyzation to form the free amine along with NaOH. Varying the *Q*_DES_:*Q*_Substrate_ ratio affects not only the statistical probability of both scenarios but also impacts the hydrodynamics and mass transfer within the system. Specifically, it affects the magnitude of flow recirculation and flow patterns, including the presence of Dean vortices with radial and transverse flows, resulting from the biphasic flow, typically presented as segmented flow. This phenomenon is known as Taylor–Dean flow.^57^ Indeed, adjusting the ratio of *Q*_DES_:*Q*_Substrate_ between 18:2 and 2:18 has a significant impact on the yield of 4**a**, as shown in Table 2, with yields ranging from 45% (lowest) to 86% (highest). Notably, an optimum *Q*_DES_:*Q*_Substrate_ ratio of 10:10 leads to a yield of 86%. Interestingly, if the reaction is carried out in neat toluene, **4a** is formed in comparable yields, demonstrating that, under the optimized reaction conditions employed, it is possible to minimize the competing decomposition of the sodium amide in the presence of DES.

**Table 2:**
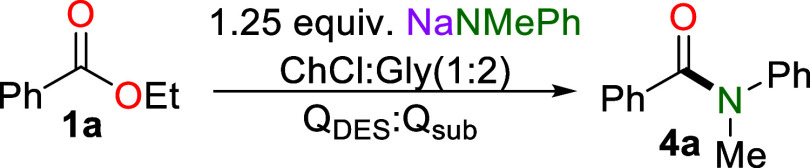
Effect of the *Q*_DES_/*Q*_Substrate_ Ratio on the Continuous Amination of Ethyl Benzoate **1a** with *In-*Situ-Formed NaNMePh^a^

*Q*_DES_/*Q*_substrate_	yield (%)^a^
2/18	54
5/15	76
10/10	86
15/5	68
18/2	45

a Reactions performed on a 1 mmol output. Hexamethylbenzene was added to the substrate stock solution to quantify yield by ^1^H NMR.

Clogging is a common problem in flow reactors when using polar organometallics,^58^ yet in all cases no clogging is observed in Reactor II (for all flow rates tested). In addition, high amidation yields are obtained with close to stoichiometric (1.25 equiv) NaHMDS concentrations, higher than the ones achieved in batch systems (Table 1). Visualization of the segmented flow of the substrate-containing organic phase (toluene) and the DES was achieved by dissolving acridine orange dye in the DES stream. This enhances the contrast between the phases, facilitating visualization and postprocessing inspection (using a Matlab-based code). Figure 4 shows the periodicity and the DES distribution in the segmented flow for a total outlet flow rate of 1 mL/min. The analysis includes both the mixing stage (DES and the substrate) and the reaction stage (DES, the substrate, and sodium amide). Refer to the Supporting Information for further details.

**Figure 4:**
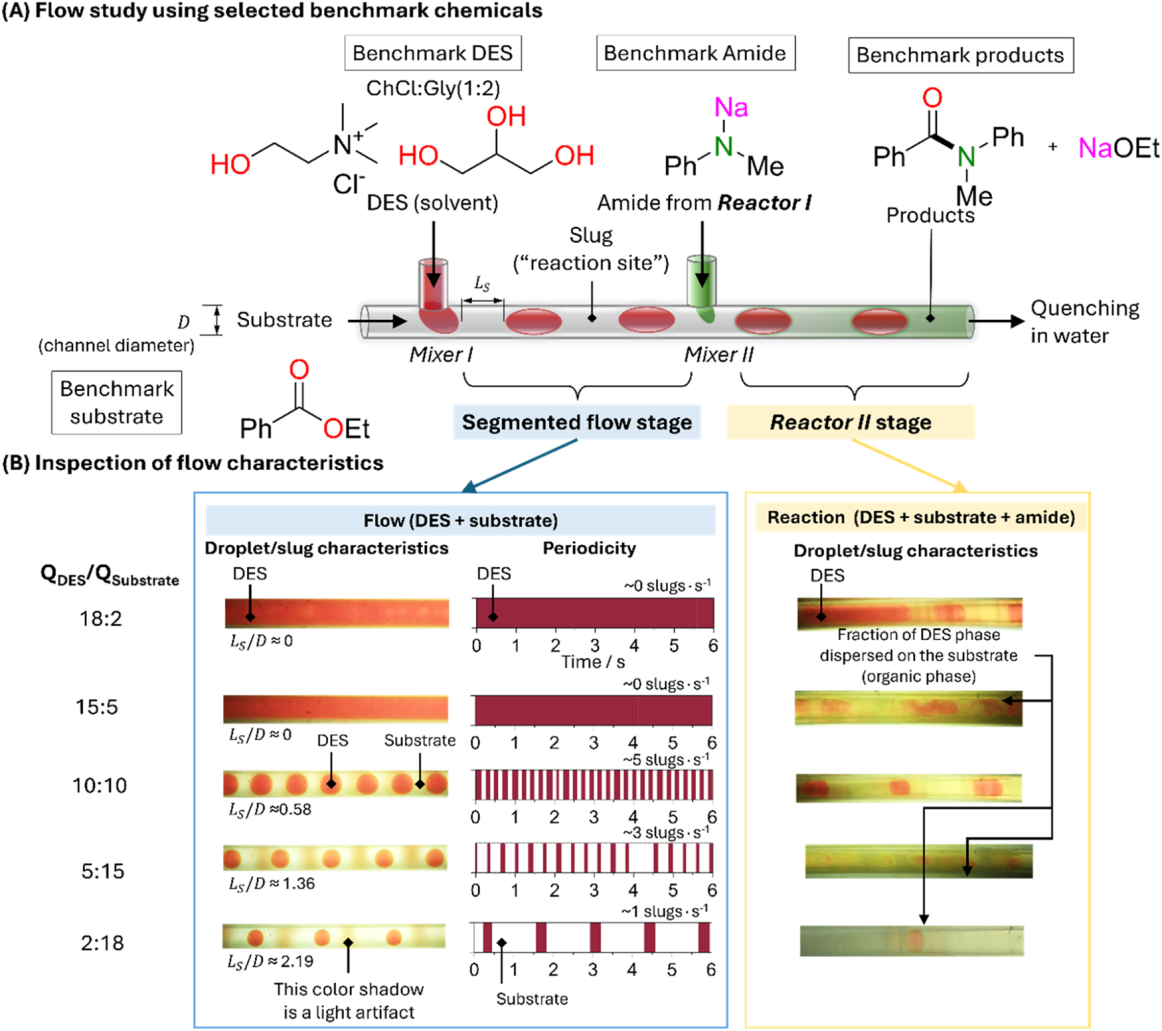
Visualization of segmented flow formation by mixing of immiscible substrate-containing organic phase and DES (ChCl:Gly (1:2)) and after its mixing with the Reactor I outlet stream containing the amide in THF in Reactor II. Acridine orange was dissolved in the DES to facilitate the DES visualization. The total flow rate is 1 mL/min at the end of Reactor II. (a) Representation of flow study using selected benchmark chemicals; and (b) Inspection of flow characteristics.

While both *Q*_DES_:*Q*_Substrate_ ratios of 18:2 and 15:5 do not lead to segmented flow, the yield rises from 45% (18:2) to 68% (15:5) due to the more defined segmented flow produced in the latter case after the addition of the amide-containing stream from Reactor I. In both cases, the relatively low yield values are associated with the high degree of hydrolyzation of the NaNMePh amide as it is in direct contact with the DES. As aforementioned, a *Q*_DES_:*Q*_Substrate_ ratio of 10:10 maximizes the yield (86%), where a high periodicity of well-defined DES droplets (∼5 droplets per second) is achieved at the mixing stage (Figure 4). However, this periodicity is disrupted following the addition of the amide-containing stream from reaction stage I due to the surfactant effect associated with NaNMePh. Decreasing the *Q*_DES_:*Q*_Substrate_ ratio leads to a lower periodicity of DES droplets, which is translated into lower yields (76% and 54% for *Q*_DES_:*Q*_Substrate_ ratios of 15:3 and 18:2, respectively). Decreasing the *Q*_DES_:*Q*_Substrate_ ratio (lower DES content) decreases the periodicity of the DES droplets with two opposite effects. On one hand, decreasing the periodicity of the DES droplets decreases the probability of the amide-containing stream being injected in direct contact with the DES, leading to their hydrolyzation (effectively increasing the yield). On the other hand, decreasing the periodicity of the DES droplets is translated to a lower contact area between the organic and DES phases, with detrimental effects on the stability of the amide species.

To better understand the importance of the contact area between the organic and DES phases and its effect on the amide hydrolyzation degree, the reaction was repeated using 2-iodoaniline as an amine. In this way, the iodine group enables tracking the distribution of the *in situ*-formed sodium amide in the organic and the DES phases, by measuring the iodine content by ICP-MS after separating both phases at the outlet of the reactor (see SI for further details). Increasing the *Q*_DES_:*Q*_Substrate_ ratio from 10:10 to 15:5 to 18:2 increases the amount of hydrolyzed sodium amide from 2.5% to 7.5% to 14.3% respectively, reflecting a similar trend to the yield values presented in Figure 4. While increasing the contact area between the DES and organic phases enhances the amide hydrolysis, an intimate contact between phases is also critical to achieving high yields, as discussed above.

Further evidence of the beneficial effect of the presence of DES and its intimate contact with the organic phase is shown by varying the total flow rate in Reactor II from the standard 1 mL/min down to 0.5 mL/min (keeping the residence time constant). We have previously demonstrated that decreasing the flow rate under Taylor–Dean flow (biphasic flow in a coiled reactor) decreases the magnitude of the Taylor and Dean vortices^57,59^ and, as a consequence, decreases the mass transfer between both phases. Indeed, decreasing the total flow rate from 1 to 0.5 mL/min for a *Q*_DES_:*Q*_substrate_ of 10:10 led to a drop in yield from 86% to 63%. An even more significant decrease is observed at a *Q*_DES_:*Q*_substrate_ of 2:18, where the yield drops from 54% to 9% due to the low periodicity of droplets under these conditions. This aligns with the batch observations whereby no stirring drastically reduced the yield.^34,54–56^ Conversely, in cases where no segmented flow is achieved (*i.e*., high DES content, such as a *Q*_DES_:*Q*_substrate_ of 18:2), no change in yield is observed when the total flow rate is decreased. Higher flow rates of 2 mL/min were also tested with little to no improvement over 1 mL/min yields.

To further explore the impact of this work, substrate screening was carried out. To maximize the volume of DES used, a *Q*_DES_:*Q*_substrate_ ratio of 15:5 and 1.5 equiv of sodium amide were used (Figure 5). The total flow rate at the outlet (of Reactor II) was kept at 1 mL/min. These conditions led to a similar **4a** yield of 83%. Comparably high yields (78–80%) are encountered using halogen-substituted benzoate esters **1b**–**1d** which furnished amides **4b**–**4d** (Figure 5). These substrates are expected to have similar diffusion coefficients to **4a** and, thus, similar hydrodynamic conditions under flow. The data also demonstrates good functional group tolerance of the sodium amide. Electron rich esters can also effectively undergo amidation reactions as shown for ethyl 4-methoxybenzoate providing **4e** in 83% yield, showing similar reactivity to that previously observed using 2-MeTHF as a solvent in batch conditions.^49^ Esters containing heterocyclic substitutes such as ethyl furan-2-carboxylate and ethyl nicotinate also lead to high yields, such as 70% and 71%for **4f** and **4g**, respectively. Similarly, ethyl 2-naphthoate gave amide **4h** in 74% yield. Long chain ester ethyl pentanoate **1i** can be effectively amidated with **4k**, forming in 92% yield. Contrastingly, shorter chained esters such as ethyl acetate and ethyl trifluoroacetate afford **4j** and **4k** in low yields of 27% and 32% respectively. It should be noted that these substrates show high yields in 2-MeTHF using less activated amines.^49^ The difference is attributed to the high solubility of these particular esters in DES, induced by their polar nature compared to **1i**, which is insoluble in ChCl:Gly (1:2). By being soluble in DES, the substrate is transferred from the organic phase to the DES phase, thereby reducing its interaction with sodium amide in the organic phase. This phenomenon has been previously reported in similar biphasic reactions conducted in batch using glycerol.^54^ This hypothesis is confirmed by decreasing the *Q*_DES_:*Q*_substrate_ ratio from 10:10 to 2:18, increasing the yield from 27% to 74% (see SI for more details). Another point of interest is that this trend is contrary to the one observed with DES insoluble substrates, as shown above. Further support on the importance of adjusting the relative solubility of the organic substrate in the DES has been recently reported in a study which investigates the addition of the Grignard reagent *i*PrMgCl in THF to acetophenone using ChCl:Gly (1:2). By combining experimental liquid diffraction, neutron reflectometry, NMR, interfacial tension measurements, and computational modeling, it has been shown that the DES is a poor solvent for the ketone, promoting its accumulation at the DES surface, which seems to be key to favor the addition reaction of the Grignard reagent to form the relevant tertiary alcohol.^56^

**Figure 5:**
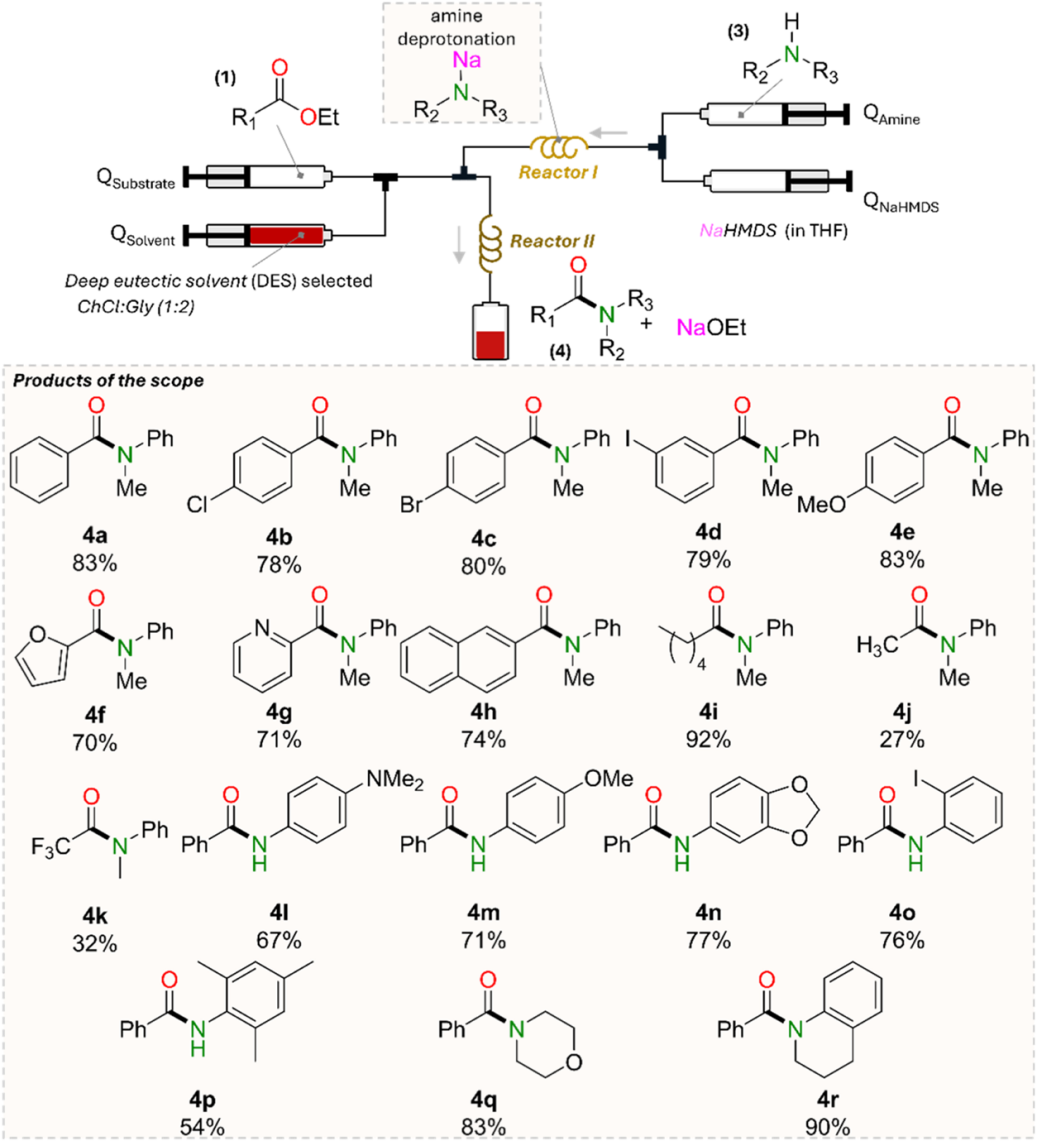
Substrate screening for the amidation reaction in flow with in situ-synthesized sodium amide. Reaction conditions: 1.5 equiv of NaNR_2_ with a *Q*_DES_/*Q*_Sub_ ratio of 15:5, Q_DES_ = 0.428 mL/min, *Q*_sub_ = 0.143 mL/min (1.05 M), *Q*_amine_ = 0.316 mL/min (0.71 M), and *Q*_NaHMDS_= 0.112 mL/min (2 M). Hexamethylbenzene was added to the ester stock solution to quantify yield by ^1^H NMR. The total flow rate at the outlet (or reactor II) was kept at 1 mL/min.

Next, to explore the stability of the flow system, the ethyl benzoate amidation reaction with *N*-methylaniline was scaled up to 10 mmol. Using the same conditions as shown above for a total run time of 45 min, no signs of clogging were observed during the reaction, showing the stability of the reactor even over longer reaction times. The product **4a** was isolated in a yield of 69% after recrystallization. Regarding the sustainability of this process, its atom economy is 64% and for the scale-up reaction, its calculated *E*-factor is 82.88 (see SI for details), which is significantly lower than that calculated when using LiHMDS in toluene under batch reaction conditions^48^ (307.11 for a 1 gr scale reaction, see SI for details). It should also be noted that while sodium is the fourth most abundant metal in the earth crust, lithium is significantly less abundant (2.3% vs 0.002%).^4^ From an operational perspective, herein, the use of DES in continuous flow spares the use of strict inert atmosphere conditions, and the solvents employed do not require to be dried or distilled prior to their utilization.

Screening a range of amines, it was found that 190 s is sufficient for the deprotonation step in Reactor I. A range of primary amines, such as *N1,N1*-dimethylbenzene-1,4-diamine, *p*-anisidine, 3,4-(methylenedioxy)­aniline, and 2-iodoaniline, gave **4l**–**4n** in yields ranging from 67 to 77%. More bulky amines such as mesityl amine give slightly lower yields with just 54% of **4p**. Interestingly, when using more nucleophilic morpholine and tetrahydroquinoline, amides **4q** and **4r** were obtained in excellent yields (83% and 90% respectively). This is particularly surprising since performing the same reaction in batch conditions furnished **4q** and **4r** in significantly lower yields (49% and 43% respectively). This yield enhancement is associated with the optimum interaction between the DES and organic phases in flow and the beneficial presence of the DES in agreement with the above. In fact, carrying the same reaction in toluene/THF in the absence of DES in batch yields only 25% of **4q** and 64% of **4r**.

Demonstrating the wider applicability of the use of sodium amides in DES under continuous flow conditions, the reactivity of a selection of in situ-prepared amides was assessed for C–F bond amidation reactions via nucleophilic aromatic substitution, using 2,6-difluoropyridine as a model substrate (Figure 6 and SI for details). In such reactions, the byproduct is NaF, which is completely insoluble in organic solvents, making very challenging the upgrade of these reactions to continuous flow. Using NaNMePh as amide in DES, the reaction with 2,6-difluoropyridine, takes place without clogging, furnishing **5a** in high yields (82%). Remarkably, when the reaction is carried out in toluene (without DES), eventual clogging of the reactor is observed, evidencing the additional role of the DES in the transportation of such insoluble byproducts. Attempts to dissolve isolated NaF into ChCl:Gly (1:2) led to no dissolution; however, the formation of a thin dispersion is observed under stirring. In THF, NaF agglomerates and precipitates, similar to NaOH (see Figure S7 in SI). This approach could also be extended to *p*-anisidine, 3,4-(methylenedioxy)­aniline, and tetrahydroquinoline, furnishing **5b**, **5c**, and **5e** in 79%, 75%, and 46% yields, respectively.

**Figure 6:**
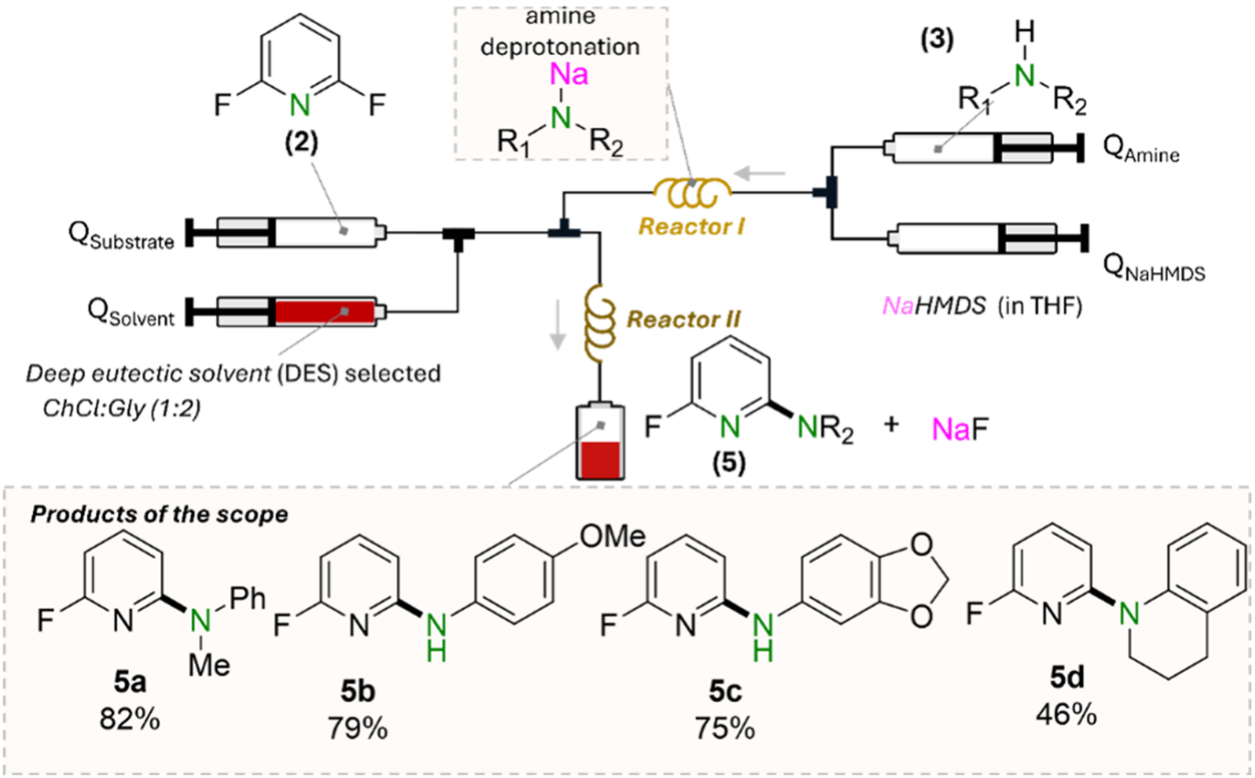
Amide screening for the nucleophilic aromatic substitution of 2,6-difluoropyridine in flow with an in situ-synthesized sodium amide. Reaction conditions: 1.5 equiv of NaNR_2_ with a *Q*_DES_: *Q*_Sub_ ratio of 15:5, *Q*_DES_ = 0.428 mL/min, *Q*_sub_ = 0.143 mL/min (1.05 M), *Q*_amine_ = 0.316 mL/min (0.71 M), and *Q*_NaHMDS_= 0.112 mL/min (2 M). 1.5 equiv of NaNR_2_ was used in the reaction. Hexamethylbenzene was added to the 2,6-difluoropyridine stock solution to quantify yield by ^1^H NMR. The total flow rate at the outlet of Reactor II was kept at 1 mL/min.

While the constitution of sodium amides in solution has not been that well studied,^4^ solvent and donor effects are known to play a key role in reactivity. Due to the protic nature of the DES, the formation of sodium amide was studied in THF, which is the solvent in which the amides are formed in the flow system. For that, tetrahydroquinoline (THQH) and morpholine (MorphH) were selected as amines to assess their constitution in the solid state and in solution. The respective sodium amides were synthesized as solids by reacting ^n^BuNa with equimolar amounts of the relevant amine in hexane. Once formed, these sodium amides were solvated by the addition of THF, with storage at −30 °C furnishing Na­(THQ) (**6**) and Na­(Morph) (**7**) in 72% and 42% isolated crystalline yields, respectively. X-ray crystallographic structures established the centrosymmetric structures of **6** and **7** (Figure 7). In the case of **6**, a discrete dimeric motif was observed, where both Na atoms connect via two amide bridges [Na1–N1, 2.3783(10) Å], and each sodium is solvated by two molecules of THF. A secondary pi-electrostatic interaction between the Na and one of the aromatic carbons of the amide is observed [Na1–C5, 3.1029(11) Å]. The lithium congener of **6** previously reported is also dimeric with bis-THF solvation of each Li atom.^60^ **7** contains a similar unit exhibiting a morphilide bridge to the two sodium atoms Na–N 2.3880(11) Å but with each being solvated by one THF ligand. The sodium is instead coordinatively saturated through solvation by neighboring morphilide groups through coordination of the oxygen [Na–O, 2.3153(8) Å] in the ring, forming a grid-like, two-dimensional polymeric layer.

**Figure 7:**
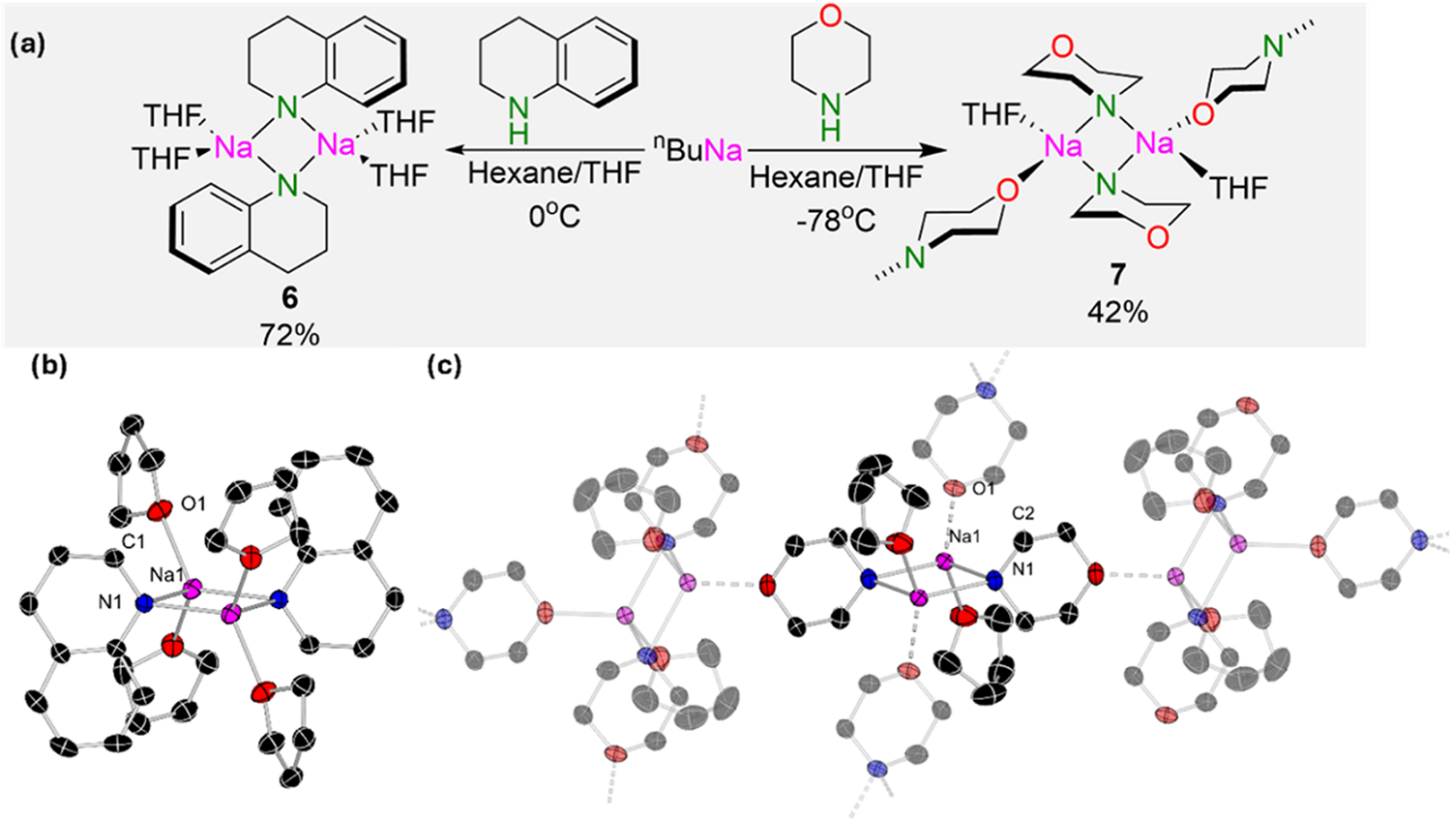
(a) Synthesis of sodium amides **6** and **7** and (b) solid-state structure of **6**. Thermal ellipsoids are shown at 30% probability and hydrogen atoms. Selected bond lengths [Å]: Na1–O1 2.3571(10), Na1 O2 2.3110(10), Na1–N1 2.3783(10), N1–C1 1.4571(15). (c) Solid-state structure of **7**. Thermal ellipsoids are shown at 30% probability and hydrogen atoms. Selected bond lengths [Å]: Na1–O1 2.315(8), Na1–O2 2.3614(10),Na–N1 2.3880(11), N1 C2 1.4424(11).

To better understand the aggregation of the sodium amides in solution, ^1^H DOSY NMR (diffusion ordered spectroscopy), along with Stalke’s ECC method for MW estimation,^61^ was used. In *d*_8_-THF, the results imply that **6** disaggregates to form a trisolvated monomer [Na­(THQ)­(THF)_3_] (estimated MW 358 g mol^–1^, error, 4%, see SI for details), whereas **7** disaggregates its extended polymeric structure to form a discrete disolvated dimer

[{Na­(Morph)­(THF)}_2_] (estimated MW 374 g mol^–1^, error, 3%). In both **6** and **7**, the disaggregation of these structures from their solid state structure aligns similarly to work by Jackman and Willard on progressive solvation of lithium amides,^60,62^ in which the solvation progresses from disolvated dimers AM_2_N_2_S_2_ to trisolvated monomers AMNS_3_ depending on the solvent present as well as the substituents on the amide N atom.

Mechanistic studies by Szostak using density functional theory (DFT) calculations for the LiHMDS-mediated amidation of esters in toluene suggest the formation of mixed-amide aggregate [Li_2_(HMDS)­(NR_2_)] dimers, which can undergo C–N bond formation with the relevant ester.^47^ While our studies involve the use of strongly coordinating THF as a solvent, which can greatly influence the speciation and aggregation of the organometallic intermediates, it should be noted that the low solubility of sodium morpholide **7** in THF under batch conditions precluded a direct comparison of its reactivity under these conditions with the results using the continuous flow approach, in which no clogging or solid formation is observed. These observations are consistent with the possible formation of a related mixed-amide aggregate [(THF)_*x*_Na_2_(HMDS)­(Morph)], which can be envisioned as an equilibrium cocomplex.^6,63^ It is also worth commenting that NaHMDS is sterically too bulky to undergo addition with the ester substrates, which minimizes the opportunities for the formation of competing side products and drives the equilibrium toward the formation of the relevant sodium amide.

## Conclusions

Advancing the applications of polar organometallic reagents in unconventional and environmentally benign solvents, we report the efficient amidation of esters using air- and moisture-sensitive sodium amides in DES using continuous flow. This method is applicable to a wide range of sodium amides, which can be prepared in flow using commercially available NaHMDS. Edging closer to stoichiometric conditions, the final organic products are obtained in good to excellent yields while using only 1.5 equiv of the relevant sodium amide. The *in situ*-generated sodium amides can also promote C–F bond amidation of 2,6-difluoropyridine via nucleophilic aromatic substitution. Key to the success of these approaches has been the development of a flow system operating under Taylor–Dean flow, segmented flow in a coiled reactor, which facilitates the mixing within the phases, optimizing the contact between the organic and DES phases, which ultimately allows for a significant reduction of the amount of organic solvent employed. In addition, the DES media avoids the agglomeration of sodium salts NaOMe or NaF obtained as stoichiometric byproducts in these reactions, allowing the flow reactor to operate efficiently without clogging problems.

We have also expanded the understanding of the constitution of sodium amides in the solid state and in THF solutions, showing that in this donor solvent, these compounds exist as kinetically activated monomeric and dimeric species. Collectively, these findings unlock the potential of highly polar, reactive, and air- and moisture-sensitive sodium amides in using DES, solvents which a priori, one would have expected to be incompatible with these reagents due to their protic nature. Furthermore, the DES not only facilitates these reactions but also allows for the generation of a unique biphasic system when working in continuous flow that can maximize conversion to the final products while operating under a more sustainable regime.

## Supplementary Material





## Notes

Full synthetic, spectroscopic, and crystallographic details (PDF)
Structures of **6** and **7** established by X-ray crystallographic analysis (CIF)

## References

[1] Wietelmann U., Klett J. (2018). 200 Years of Lithium and 100 Years of Organolithium Chemistry. Z. Anorg. Allg. Chem..

[2] Clayden, J. Organolithiums: Selectivity for Synthesis; Elsevier Science/Pergamon: Amsterdam, 2003; pp 365–377.

[3] Mulvey R. E., Robertson S. D. (2013). Synthetically Important Alkali-Metal Utility Amides:
Lithium, Sodium, and Potassium Hexamethyldisilazides, Diisopropylamides,
and Tetramethylpiperidides. Angew. Chem., Int.
Ed..

[4] Anderson D. E., Tortajada A., Hevia E. (2024). New Frontiers in Organosodium Chemistry
as Sustainable Alternatives to Organolithium Reagents. Angew. Chem., Int. Ed..

[5] Hans
Wedepohl K. (1995). The Composition of the Continental Crust. Geochim. Cosmochim. Acta.

[6] Tortajada A., Hevia E. (2022). Perdeuteration of Arenes
via Hydrogen Isotope Exchange Catalyzed
by the Superbasic Sodium Amide Donor Species NaTMP·PMDETA. J. Am. Chem. Soc..

[7] Asako S., Takahashi I., Nakajima H., Ilies L., Takai K. (2021). Halogen–Sodium
Exchange Enables Efficient Access to Organosodium Compounds. Commun. Chem..

[8] Asako S., Kodera M., Nakajima H., Takai K. (2019). Lithium-Free
Synthesis
of Sodium 2,2,6,6-Tetramethylpiperidide and Its Synthetic Applications. Adv. Synth. Catal..

[9] Harenberg J. H., Weidmann N., Wiegand A. J., Hoefer C. A., Annapureddy R. R., Knochel P. (2021). 2-Ethylhexyl)­Sodium: A Hexane-Soluble Reagent for Br/Na-Exchanges
and Directed Metalations in Continuous Flow. Angew. Chem., Int. Ed..

[10] Harenberg J. H., Annapureddy R. R., Karaghiosoff K., Knochel P. (2022). Continuous Flow Preparation
of Benzylic Sodium Organometallics. Angew. Chem.,
Int. Ed..

[11] Natho P., Luisi R. (2023). Flow chemistry as green technology for the genesis and use of organometallic
reagents in the synthesis of key building blocks and APIs –
An update. Tetrahedron Green Chem..

[12] Dilauro G., Luccarelli C., Quivelli A. F., Vitale P., Perna F. M., Capriati V. (2023). Introducing
Water and Deep Eutectic Solvents in Organosodium
Chemistry: Chemoselective Nucleophilic Functionalizations in Air. Angew. Chem., Int. Ed..

[13] Gurkan B., Squire H., Pentzer E. (2019). Metal-Free Deep Eutectic Solvents:
Preparation, Physical Properties, and Significance. J. Phys. Chem. Lett..

[14] Liu Y., Friesen J. B., McAlpine J. B., Lankin D. C., Chen S.-N., Pauli G. F. (2018). Natural Deep Eutectic Solvents: Properties, Applications,
and Perspectives. J. Nat. Prod..

[15] Alonso D. A., Baeza A., Chinchilla R., Guillena G., Pastor I. M., Ramón D. J. (2016). Deep Eutectic
Solvents: The Organic Reaction Medium
of the Century. Eur. J. Org. Chem..

[16] García-Álvarez J. (2015). Deep Eutectic
Mixtures: Promising Sustainable Solvents for Metal-Catalysed and Metal-Mediated
Organic Reactions. Eur. J. Inorg. Chem..

[17] Rodríguez-Álvarez M. J., Vidal C., Díez J., García-Álvarez J. (2014). Introducing
Deep Eutectic Solvents as Biorenewable Media for Au­(i)-Catalysed Cycloisomerisation
of γ-Alkynoic Acids: An Unprecedented Catalytic System. Chem. Commun..

[18] Vidal C., Suárez F. J., García-Álvarez J. (2014). Deep Eutectic
Solvents (DES) as Green Reaction Media for the Redox Isomerization
of Allylic Alcohols into Carbonyl Compounds Catalyzed by the Ruthenium
Complex [Ru­(*η*3:*η*3-C10H16)­Cl2­(Benzimidazole)]. Catal. Commun..

[19] Imperato G., Höger S., Lenoir D., König B. (2006). Low Melting
Sugar–Urea–Salt Mixtures as Solvents for Organic Reactions—Estimation
of Polarity and Use in Catalysis. Green Chem..

[20] Dilauro G., García S. M., Tagarelli D., Vitale P., Perna F. M., Capriati V. (2018). Ligand-Free
Bioinspired Suzuki–Miyaura Coupling
Reactions Using Aryltrifluoroborates as Effective Partners in Deep
Eutectic Solvents. ChemSusChem.

[21] Smith E. L., Abbott A. P., Ryder K. S. (2014). Deep Eutectic
Solvents (DESs) and
Their Applications. Chem. Rev..

[22] Hansen B. B., Spittle S., Chen B., Poe D., Zhang Y., Klein J. M., Horton A., Adhikari Laxmi., Zelovich T., Doherty B. W., Gurkan B., Maginn E. J., Ragauskas A., Dadmun M., Zawodzinski T. A., Baker G. A., Tuckerman M. E., Savinell R. F., Sangoro J. R. (2021). Deep Eutectic
Solvents: A Review
of Fundamentals and Applications. Chem. Rev..

[23] Cicco L., Ríos-Lombardía N., J Rodríguez-Álvarez M., Morís F., M Perna F., Capriati V., García-Álvarez J., González-Sabín J. (2018). Programming Cascade Reactions Interfacing
Biocatalysis with Transition-Metal Catalysis in Deep Eutectic Solvents
as Biorenewable Reaction Media. Green Chem..

[24] Ríos-Lombardía N., Vidal C., Liardo E., Morís F., García-Álvarez J., González-Sabín J. (2016). From a Sequential
to a Concurrent Reaction in Aqueous Medium: Ruthenium-Catalyzed Allylic
Alcohol Isomerization and Asymmetric Bioreduction. Angew. Chem., Int. Ed..

[25] Arnodo D., De Nardi F., Parisotto S., De Nardo E., Cananà S., Salvatico F., De Marchi E., Scarpi D., Blangetti M., Occhiato E. G., Prandi C. (2024). Asymmetric Reduction of Cyclic Imines
by Imine Reductase Enzymes in Non-Conventional Solvents. ChemSusChem.

[26] Vidal C., García-Álvarez J., Hernán-Gómez A., Kennedy A. R., Hevia E. (2014). Introducing Deep Eutectic Solvents
to Polar Organometallic Chemistry: Chemoselective Addition of Organolithium
and Grignard Reagents to Ketones in Air. Angew.
Chem., Int. Ed..

[27] Vidal C., García-Álvarez J., Hernán-Gómez A., Kennedy A. R., Hevia E. (2016). Exploiting
Deep Eutectic Solvents
and Organolithium Reagent Partnerships: Chemoselective Ultrafast Addition
to Imines and Quinolines Under Aerobic Ambient Temperature Conditions. Angew. Chem., Int. Ed..

[28] Ghinato S., Dilauro G., Perna F. M., Capriati V., Blangetti M., Prandi C. (2019). Directed Ortho-Metalation–Nucleophilic Acyl
Substitution Strategies in Deep Eutectic Solvents: The Organolithium
Base Dictates the Chemoselectivity. Chem. Commun..

[29] García-Álvarez J., Hevia E., Capriati V. (2015). Reactivity of Polar Organometallic
Compounds in Unconventional Reaction Media: Challenges and Opportunities. Eur. J. Org. Chem..

[30] García-Álvarez J., Hevia E., Capriati V. (2018). The Future of Polar Organometallic
Chemistry Written in Bio-Based Solvents and Water. Chem.-Eur. J..

[31] Hevia E. (2020). Towards a
Paradigm Shift in Polar Organometallic Chemistry. Chimia.

[32] Gentner T. X., Mulvey R. E. (2021). Alkali-Metal Mediation: Diversity of Applications in
Main-Group Organometallic Chemistry. Angew.
Chem., Int. Ed..

[33] Perna F. M., Vitale P., Capriati V. (2021). Synthetic applications of polar organometallic
and alkali-metal reagents under air and moisture. Curr. Opin. Green Sustainable Chem..

[34] García-Garrido S. E., Presa
Soto A., Hevia E., García-Álvarez J. (2021). Advancing
Air- and Moisture-Compatible s-Block Organometallic Chemistry Using
Sustainable Solvents. Eur. J. Inorg. Chem..

[35] Mulks F. F., Pinho B., Platten A. W. J., Andalibi M. R., Expósito A. J., Edler K. J., Hevia E., Torrente-Murciano L. (2022). Continuous,
Stable, and Safe Organometallic Reactions in Flow at Room Temperature
Assisted by Deep Eutectic Solvents. Chem.

[36] Mulks F. F., Bole L. J., Davin L., Hernán-Gómez A., Kennedy A., García-Álvarez J., Hevia E. (2020). Ambient Moisture Accelerates Hydroamination Reactions of Vinylarenes
with Alkali-Metal Amides under Air. Angew. Chem.,
Int. Ed..

[37] Woltornist R. A., Ma Y., Algera R. F., Zhou Y., Zhang Z., Collum D. B. (2020). Structure,
Reactivity, and Synthetic Applications of Sodium Diisopropylamide. Synthesis.

[38] Ma Y., Lui N. M., Keresztes I., Woltornist R. A., Collum D. B. (2022). Sodium Isopropyl­(Trimethylsilyl)­Amide: A Stable and
Highly Soluble Lithium Diisopropylamide Mimic. J. Org. Chem..

[39] McGrath N. A., Brichacek M., Njardarson J. T. (2010). A Graphical Journey of Innovative
Organic Architectures That Have Improved Our Lives. J. Chem. Educ..

[40] Constable D. J. C., Dunn P. J., Hayler J. D., Humphrey G. R., Leazer J. L., Linderman R. J., Lorenz K., Manley J., Pearlman B. A., Wells A., Zaks A., Zhang T. Y. (2007). Key Green
Chemistry Research Areas—a Perspective from Pharmaceutical
Manufacturers. Green Chem..

[41] Bryan M. C., Dunn P. J., Entwistle D., Gallou F., Koenig S. G., Hayler J. D., Hickey M. R., Hughes S., Kopach M. E., Moine G., Richardson P., Roschangar F., Steven A., Weiberth F. J. (2018). Key Green Chemistry Research Areas
from a Pharmaceutical Manufacturers’ Perspective Revisited. Green Chem..

[42] Charville H., Jackson D. A., Hodges G., Whiting A., Wilson M. R. (2011). The Uncatalyzed
Direct Amide Formation Reaction – Mechanism Studies and the
Key Role of Carboxylic Acid H-Bonding. Eur.
J. Org. Chem..

[43] Romano S., Rescifina A., De Luca G., Nardi M., Oliverio M., Procopio A. (2023). New Insights on Choline Chloride Role in Synthesis:
The Case of Direct Amidation,. ACS Sustainable
Chem. Eng..

[44] Massolo E., Pirola M., Benaglia M. (2020). Amide Bond Formation Strategies:
Latest Advances on a Dateless Transformation. Eur. J. Org. Chem..

[45] Acosta-Guzmán P., Ojeda-Porras A., Gamba-Sánchez D. (2023). Contemporary Approaches
for Amide Bond Formation. Adv. Synth. Catal..

[46] de
Figueiredo R. M., Suppo J.-S., Campagne J.-M. (2016). Nonclassical Routes
for Amide Bond Formation. Chem. Rev..

[47] Li G., Szostak M. (2018). Highly Selective Transition-Metal-Free
Transamidation
of Amides and Amidation of Esters at Room Temperature. Nat. Commun..

[48] Li G., Ji C.-L., Hong X., Szostak M. (2019). Highly Chemoselective,
Transition-Metal-Free Transamidation of Unactivated Amides and Direct
Amidation of Alkyl Esters by N–C/O–C Cleavage. J. Am. Chem. Soc..

[49] Fairley M., Bole L. J., Mulks F. F., Main L., Kennedy A. R., O’Hara C. T., García-Alvarez J., Hevia E. (2020). Ultrafast
Amidation of Esters Using Lithium Amides under Aerobic Ambient Temperature
Conditions in Sustainable Solvents. Chem. Sci..

[50] You Q., Ma Y., Woltornist R. A., Lui N. M., Spivey J. A., Keresztes I., Collum D. B. (2024). Sodium Alkyl­(trimethylsilyl)­amides:
Substituent- and Solvent-dependent Solution Structures and Reactivities. J. Am. Chem. Soc..

[51] Cicco L., Sblendorio S., Mansueto R., Perna F. M., Salomone A., Florio S., Capriati V. (2016). Water Opens the Door to Organolithiums
and Grignard Reagents: Exploring and Comparing the Reactivity of Highly
Polar Organometallic Compounds in Unconventional Reaction Media towards
the Synthesis of Tetrahydrofurans. Chem. Sci..

[52] Perna F. M., Vitale P., Capriati V. (2020). Deep Eutectic
Solvents and Their
Applications as Green Solvents. Curr. Opin.
Green Sustainable Chem..

[53] Tortajada A., Anderson D. E., Hevia E. (2022). Gram-Scale Synthesis,
Isolation and
Characterisation of Sodium Organometallics: nBuNa and NaTMP. Helv. Chim. Acta.

[54] Rodríguez-Álvarez M. J., García-Álvarez J., Uzelac M., Fairley M., O’Hara C. T., Hevia E. (2018). Introducing Glycerol as a Sustainable
Solvent to Organolithium Chemistry: Ultrafast Chemoselective Addition
of Aryllithium Reagents to Nitriles under Air and at Ambient Temperature. Chem.-Eur. J..

[55] Platten A. W. J., Manasi I., Campana M., Edler K. J., Hevia E. (2025). Harnessing
Deep Eutectic Solvents for Regioselective Polar Additions to α,
β Unsaturated Ketones and Aldehydes. ChemSusChem.

[56] Manasi I., Bortoli M., Bowron D. T., Campana M., Hammond O. S., Headen T. F., Hooton J., Hevia E., Cascella M., Eisenstein O., Edler K. J. (2025). Are Grignard Reactions in Deep Eutectic
Solvents Interface-Driven?. Angew. Chem., Int.
Ed..

[57] Pinho B., Williams L. M., Mahin J., Gao Y., Torrente-Murciano L. (2023). Enhancing
Mixing Efficiency in Curved Channels: A 3D Study of Bi-Phasic Dean-Taylor
Flow with High Spatial and Temporal Resolution. Chem. Eng. J..

[58] Machida K., Kawachi H., Iwasaki R., Sakaguchi T., Murakami A., Nishiyama A. (2024). Efficient Cleaning Method for Flow
Reactors in Flow Lithiation Reactions Under Water-Free Conditions. Org. Process Res. Dev..

[59] Wu K.-J., De Varine Bohan G. M., Torrente-Murciano L. (2017). Synthesis
of Narrow Sized Silver
Nanoparticles in the Absence of Capping Ligands in Helical Microreactors. React. Chem. Eng..

[60] Su C., Guang J., Williard P. G. (2014). Structures of Lithium N-Monosubstituted
Anilides: Trisolvated Monomer to Tetrasolvated Dimer. J. Org. Chem..

[61] Neufeld R., Stalke D. (2015). Accurate Molecular Weight Determination of Small Molecules
via DOSY-NMR by Using External Calibration Curves with Normalized
Diffusion Coefficients. Chem. Sci..

[62] Jackman L. M., Scarmoutzos L. M. (1987). Structures
of the Lithium Salts of Aromatic Secondary
Amines in Weakly Polar Aprotic Solvents. J.
Am. Chem. Soc..

[63] Tschopp M. S., Platten A. W. J., Hevia E., Tortajada A. (2024). Development
of Sterically Hindered and Basic Sodium Amides for Catalytic Hydrogen
Isotope Exchange. Eur. J. Inorg. Chem..

