# Supplementary material for: Assessing Sodium Amide Reagents for Ester Amidations in Deep Eutectic Solvents in Continuous Flow
Source: ACS Sustain Chem Eng. 2025 Oct 31;13(44):19351–61. doi: 10.1021/acssuschemeng.5c08775 (PMC12606785; doi:10.1021/acssuschemeng.5c08775)
Supplement: Supplementary file 1 [file sc5c08775_si_001.pdf]

Supporting Information

Assessing Sodium Amide Reagents for Ester Amidations in Deep Eutectic Solvents in Continuous Flow

Andrew W. J. Platten[a‡] Bruno Pinho,[b‡] Laura Torrente-Murciano[b*] and Eva Hevia[a*]

[a]Department für Chemie Biochemie und Pharmazie, Universität Bern, Freiestrasse 3, 3012 Bern (Switzerland)

[b]Department of Chemical Engineering and Biotechnology, University of Cambridge, Philippa Fawcett Drive, Cam-bridge CB3 0AS, (UK)

Number of Pages: 50

Number of Figures (including spectra): 48

Number of Tables: 14

Table of Contents

General Considerations 4
Reagents 4
Batch reactions 4
Alkali metal Amide formation 4
Conditions screening 5
Alkali metal effect 5
Reactions in flow 6
Microreactor characteristics 6
Preparation of DES 7
Preparation of reagent solutions 7
Preparation of NaHMDS syringe 7
Product collection and analysis 7
General flow conditions for optimisation process 8
Flow visualization 10
Optimisation of the two phase flow 11
Optimisation of the total flow rate 12
Optimisation of DES 12
Effect of water content on reactivity. 13
Optimisation of the residence time 13
Quantification of the amine in each phase after reaction 13
Clogging visualisation experiments 15
General flow conditions for scope 16
Ethyl acetate effect of changing Q_{Des}/Q_{Sub} 16
Scale up reaction (10 mmol) 16
E-factor and atom economy for scale up of reaction 17
Reported spectroscopic data 18
N-methyl-N-phenylbenzamide (4a) 18
4-Chloro-N-methyl-N-phenylbenzamide (4b) 18
4-bromo-N-methyl-N-phenylbenzamide (4c) 18
3-iodo-N-methyl-N-phenylbenzamide (4d) 18
4-methoxy-N-methyl-N-phenylbenzamide (4e) 19
N-methyl-N-phenylfuran-2-carboxamide (4f) 19
N-methyl-N-phenylnicotinamide (4g) 19
N-methyl-N-phenyl-2-naphthamide (4h) 19
N-methyl-N-phenylpentanamide (4i) 20
N-methyl-N-phenylacetamide (4j) 20

2,2,2-trifluoro-N-methyl-N-phenylacetamide (4k) 20
N-(4-(dimethylamino)phenyl)benzamide (4l) 21
N-(4-methoxyphenyl)benzamide (4m) 21
N-(benzo[d][1,3]dioxol-5-yl)benzamide (4n) 21
N-(2-iodophenyl)benzamide (4o) 21
N-mesitylbenzamide (4p) 22
morpholino(phenyl)methanone (4q) 22
(3,4-dihydroquinolin-1(2H)-yl)(phenyl)methanone (4r) 22
6-fluoro-N-methyl-N-phenylpyridin-2-amine (5a) 22
6-fluoro-N-(4-methoxyphenyl)pyridin-2-amine (5b) 23
N-(benzo[d][1,3]dioxol-5-yl)-6-fluoropyridin-2-amine (5c) 23
1-(6-fluoropyridin-2-yl)-1,2,3,4-tetrahydroquinoline (5d) 24
NMR Spectra 26
X-Ray Crystallography 46
References 49

General Considerations

Caution should be taken when using organosodium reagents in air due to their high pyrophoricity. All amides formed in batch were synthesised in a Schlenk flask under argon prior to reaction using standard Schlenk line techniques.Sodium amides under flow conditions were synthesised *in situ* in the reactor.

Reagents

NaHMDS was bought from Acros and was used directly without further purification. All amides were synthesized from the commercially available amine with no further purification Ethyl benzoate was purchased from sigma Aldrich. Any reactions done under an inert atmosphere of argon were done using standard Schlenk line[1] or glove-box techniques (MBraun UNILab Pro ECO, <0.5 ppm H_2O and O_2). Dried solvents were dried using a MBraun MBSPS 5 and stored over 4 Å molecular sieves. THF-d_8 was dried and distilled over NaK alloy and stored over 4 Å molecular sieves. $CDCl_3$ was stored over 4 Å molecular sieves. NMR spectra were recorded on Bruker Avance III HD 300 or 400 MHz spectrometers. ^1H and $^{13}C\{^1H\}$ NMR spectra were referenced internally to residual solvent peaks. Hexamethylbenzene used as internal standard was purchased from sigma aldrich.

The following substrates were used in this study: **1a** Ethyl benzoate, **1b** ethyl 4-chlorobenzoate, **1c** ethyl 4-bromobenzoate, **1d** ethyl 3-iodobenzoate **1e** ethyl 4-methoxybenzoate, **1f** ethyl furan-2-carboxylate, **1g** ethyl picolinate,**1h** ethyl 2-naphthoate**,1i** ethyl hexanoate**,1j** ethyl acetate**, 1k** ethyl trifluoroacetate **2** difluoropyridine **3a** N-methylaniline**, 3b** N1,N1-dimethylbenzene-1,4-diamine **3c** 4-methoxyaniline **3d** 3,4-(Methylenedioxy)aniline **3e** 2-iodoaniline **3f** trimethylaniline **3g** morpholine **, 3h** tetrahydroquinoline

Batch reactions

Alkali metal Amide formation

In a Schlenk flask ^{n}BuLi (1.25 ml, 1.6 M, 2 mmol) ^{n}BuNa(160 mg,2 mmol), or KCH_2SiMe_3 (252 mg, 2 mmol) was suspended in hexane (5 ml) to which N-methyl aniline (0.22 ml, 2 mmol) was added at 0 °C. Forming a white suspension. After 1 h the volatiles were removed under vacumn and the colourless solid redissolved in THF (2 ml).The Alkali metal amide was then syringed out and used without further purification.

Alkali metal mediated Amidation procedure

To a 2-dram vial ethylbenzoate (1 mmol) was suspended in DES (1 g) and stirred at 1000 rpm. The relevant sodium amide was then added and left to stir for 20 seconds. After which the reaction was quenched water (1 ml). The product was extracted using EtOAc (3 x 5 ml) and washed with NH_4Cl the organic phase was dried over $MgSO_4$ and solvent removed. Spectroscopic yields were measured using hexamethylbenzene (27 mg, 0.166 mmol) as an internal standard.

Conditions screening

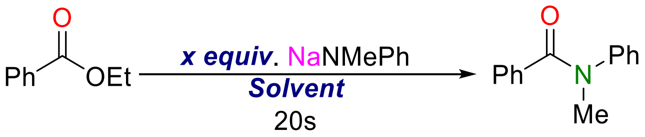

Solvent	Equivalence of alkali metal amide	% Yield of 4a
ChCl:Gly (1:2)	2	90
ChCl:EtGly (1:2)	2	65
ChCl:Urea (1:2)	2	63
Water	2	10
Glycerol	2	83
ChCl:Gly (1:2)	1	55
ChCl:Gly (1:2)	1.5	81

Table S1: Optimisation of solvent in batch

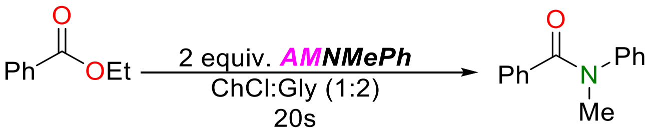

Alkali metal amide	Concentration of alkali metal amide	% yield of 4a
LiNMePh	2M	60
NaNMePh	2M	77
KNMePh	2M	51
NaNMePh	1M	90

Table S2: effect of changing concentration and alkali metal on yield of 4a

Alkali metal effect

Comparison of AMNMePh (Li,Na,K)was performed according to batch procedure but reversing the order of addition

To a 2 dram vial DES (ChCl:Gly (1:2)) (1 g) was added and stirred at 1000 rpm. AMNMePh (2 ml,1 M in THF) was spread onto the DES and left for the relevant time (0 s,15 s,30 s,45 s,60 s) at which point ethyl benzoate (0.142 ml, 1 mmol) was added and the reaction left to stir for 20 seconds. After which the reaction was quenched water (1ml). The product was extracted using EtOAc (3x5ml) and washed with NH_4Cl the organic phase was dried over $MgSO_4$ and solvent removed. Spectroscopic yields were measured using hexamethylbenzene (27.0 mg, 0.166 mmol) as an internal standard. Reported yields based on average yields of two repetitions

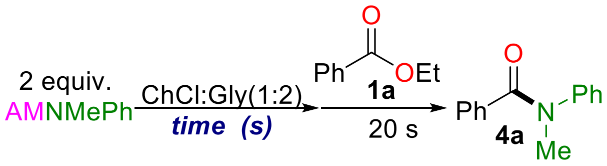

Time on surface (s)	% yield of 4a Li	Na	K
0	90	90	90
15	89	88	71
30	0	65	41
45	0	46	0
60	0	22	0

Table S3: comparison of stability of alkali metal on DES

Reactions in flow

Microreactor characteristics

The device is made of perfluoroalkoxy alkane (PFA) tubing (D = 1.01 mm for reactor 1 D = 0.76 mm for reactor 2 internal diameter), ethylene tetrafluoroethylene (ETFE)/PFA fittings, and four syringe pumps for delivering different fluids. A solution of the reactant in toluene is introduced via a T-mixer into a stream of the carrier phase (DES) to give a segmented flow regime with the droplets of the organic phase enveloped by the DES. Down the stream, a commercial solution of NaHMDS (2 M) in THF is added using a second T-mixer. Subsequently, the addition reaction takes place in a tubular microreactor (0.23 mL). The microreactor length (50 cm) allows for an excess of residencetime (tres; in the order of 40 s) to ensure the full conversion of the amide. The outlet is led into a collection vial containing water to ensure the full hydrolysis of the hazardous unreacted organo-sodium species and to quench the reaction. After reaction is complete the reactor was flushed with water followed by toluene prior to further use.

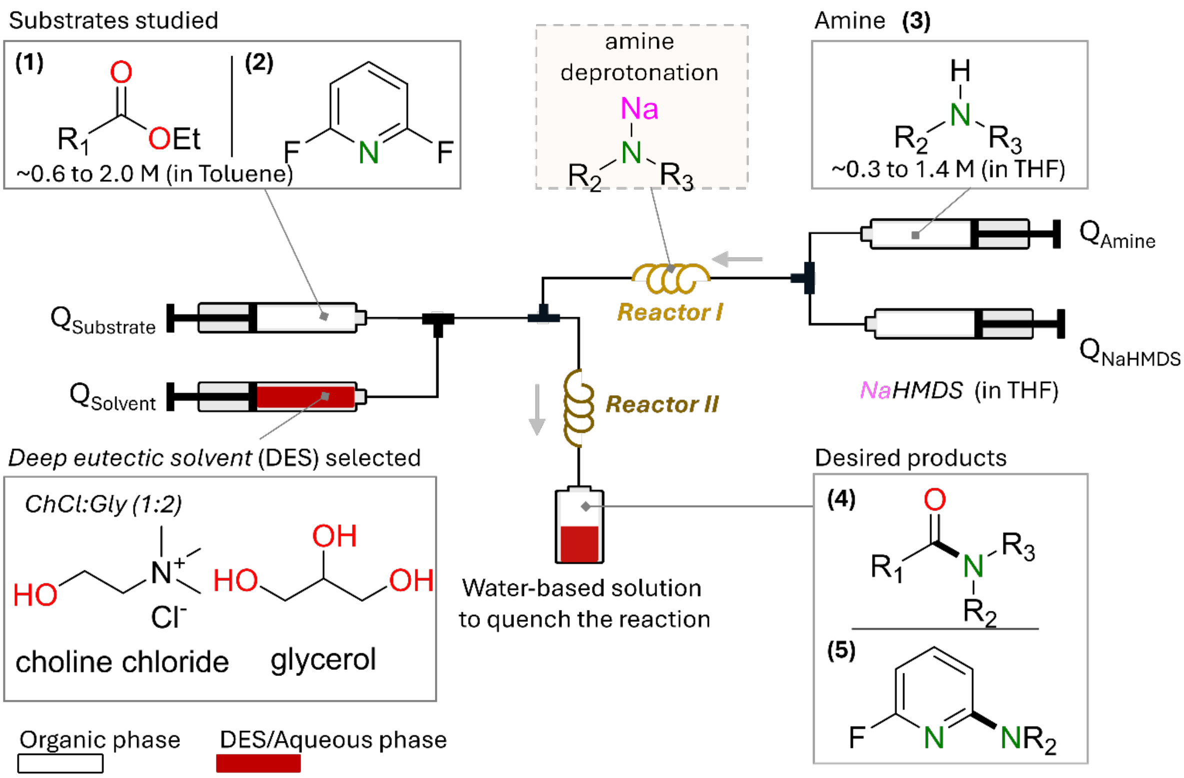

Figure S1: Flow setup.

Preparation of DES

All DES were synthesised according to literature precedent by taking the constituent components and heating in a vacuum oven storage of the DES in vacuum oven was used to prevent high water content of the DES.

Preparation of reagent solutions

Amines were measured out and dissolved in commercially available undried THF. Esters and difluoropyridine were dissolved in toluene along with Hexamethylbenzene Each of these solutions were added to 5 ml leur lock syringe to be mounted on the employed syringe pumps.

Preparation of NaHMDS syringe

NaHMDS was used as a commercially available 2M solution in THF. A positive stream of nitrogen was applied to the sure seal via tubing connected to a needle. A 5 ml Luer lock syringe was then flushed three times with nitrogen before filling with the appropriate amount of the solution. The syringe was quickly attached to the Luer lock male connector of the microreactor.

Product collection and analysis

The reactor outlet was collected in a 10 ml glass vial as waste until the reactor was flushed 2.5 times based on its volume and the involved flow rates. Then, a 10 mL glass vial was used for collecting the solution from the reactor outlet.hexamethyl benzene dissolved in the ester

stock solution was used for yield determination the reactor output. selectivity was determined based on the integral of remaining reactant, product, and (if any) side-products.

The majority of compounds prepared in this manuscript have been previously reported in the literature and their spectroscopic data have been carefully compared to those already known Any new products have been isolated and fully characterised

General flow conditions for optimisation process

1ml/min conditions					
**DES/Sub**	**Chemical**	**Concentration of stock solution (M)**	**Concentration (M)**	**Flow rate μL/min**	**Residence time reactor 1 (s)**
18/2	Ethyl benzoate	1.95	0.1950	76.92	237
	Glyceline		-	692.31	
	N-Methyl aniline	1.37	0.8125	137.02	
	NaHMDS	2	0.8125	93.75	
15/5	Ethyl benzoate	1.05	0.2625	142.86	128
	Glyceline		-	428.57	
	Amine	0.56	0.5250	334.82	
	NaHMDS	2	0.5250	93.75	
10/10	Ethyl benzoate	0.75	0.3750	200	91
	Glyceline			200	
	N-Methyl aniline	0.37	0.3125	506.25	
	NaHMDS	2	0.3125	93.75	
5/15	Ethyl benzoate	0.65	0.4875	230.77	79
	Glyceline			76.92	
	N-Methyl aniline	0.31	0.2788	598.56	
	NaHMDS	2	0.2788	93.75	
2/18	Ethyl benzoate	0.62	0.5550	243.24	74
	Glyceline			27.03	
	N-Methyl aniline	0.29	0.2569	635.98	
	NaHMDS	2	0.2569	93.75	

Table S4: *Conditions for flow reactor 1ml/min flow rate*

0.5 ml/min conditions						
**DES/Sub**	**Chemical**	**Concentration of stock solution (M)**	**Concentration (M)**	**Flow rate μL/min**	**Residence time reactor 1 (s)**	**Residence time reactor 2 (s)**
18/2	Ethyl benzoate	1.95	0.1950	46.88	474	82
	Glyceline		-	346.15		
	N-Methyl aniline	1.37	0.8125	68.51		
	NaHMDS	2	0.8125	46.87		
15/5	Ethyl benzoate	1.05	0.2625	71.43	255	82
	Glyceline		-	214.29		
	Amine	0.56	0.4375	167.41		
	NaHMDS	2	0.4375	46.87		
10/10	Ethyl benzoate	0.75	0.3750	100	182	82
	Glyceline			100		
	N-Methyl aniline	0.37	0.3125	253.13		
	NaHMDS	2	0.3125	46.88		
5/15	Ethyl benzoate	0.65	0.4875	115.38	158	82
	Glyceline			38.46		
	N-Methyl aniline	0.31	0.2788	299.28		
	NaHMDS	2	0.2788	46.88		
2/18	Ethyl benzoate	0.62	0.5550	121.62	74	82
	Glyceline			13.51		
	N-Methyl aniline	0.29	0.2569	317.99		
	NaHMDS	2	0.2569	46.88		

Table S5: Conditions for flow reactor 0.5 ml/min flow rate

Flow visualization

Visualisations of the reactor were performed in the PFA tubing with the DES dyed with acridine orange using the footage of the flow reactor in opporation the periodicidity of the reactor can be ascertained

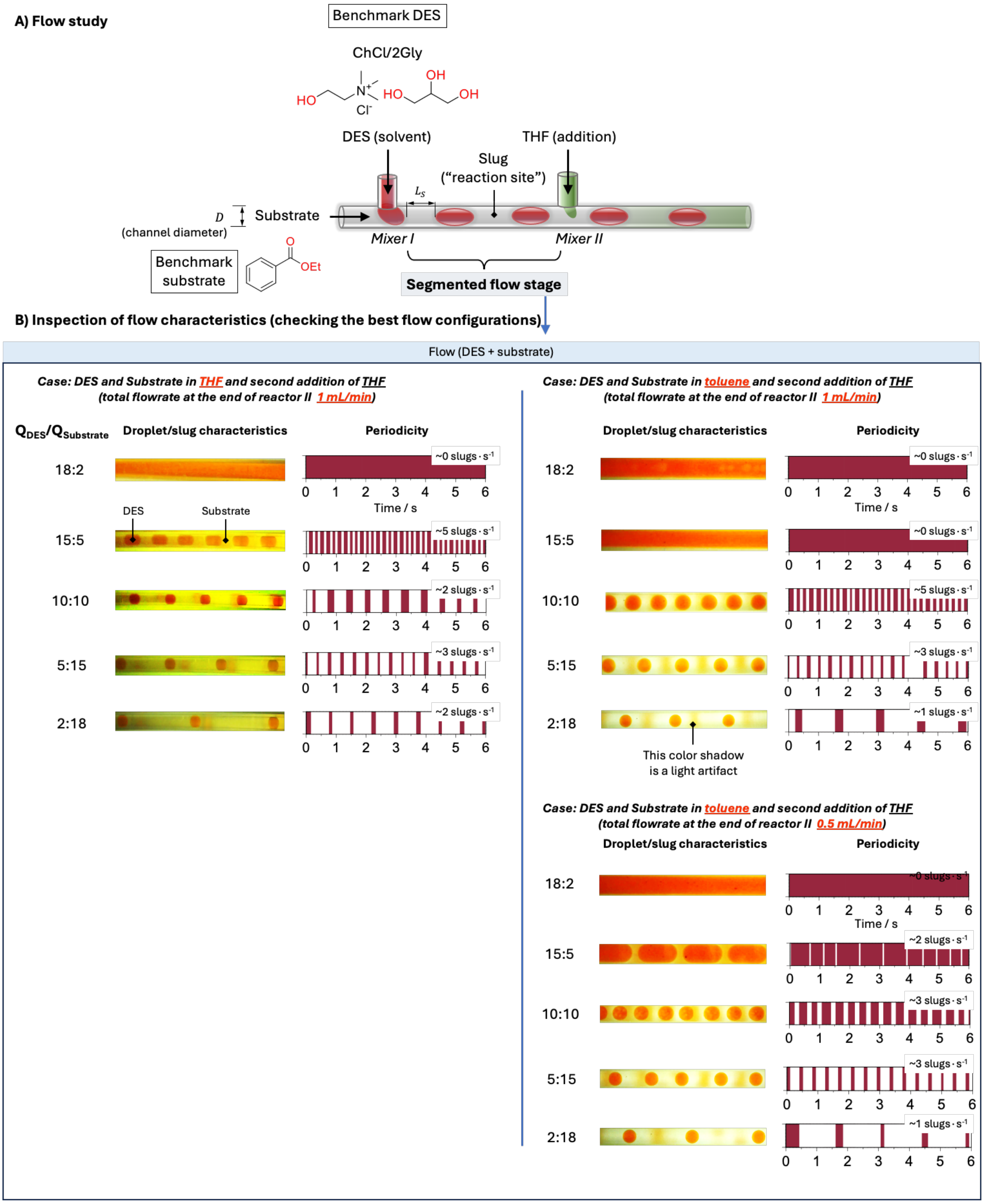

Figure S2: Visualization of the reactor at total flow rates of 0.5 and 1.0 ml/min without any reaction. Two different solvent sets were utilized: one containing the substrate in THF and the other with the substrate in toluene.

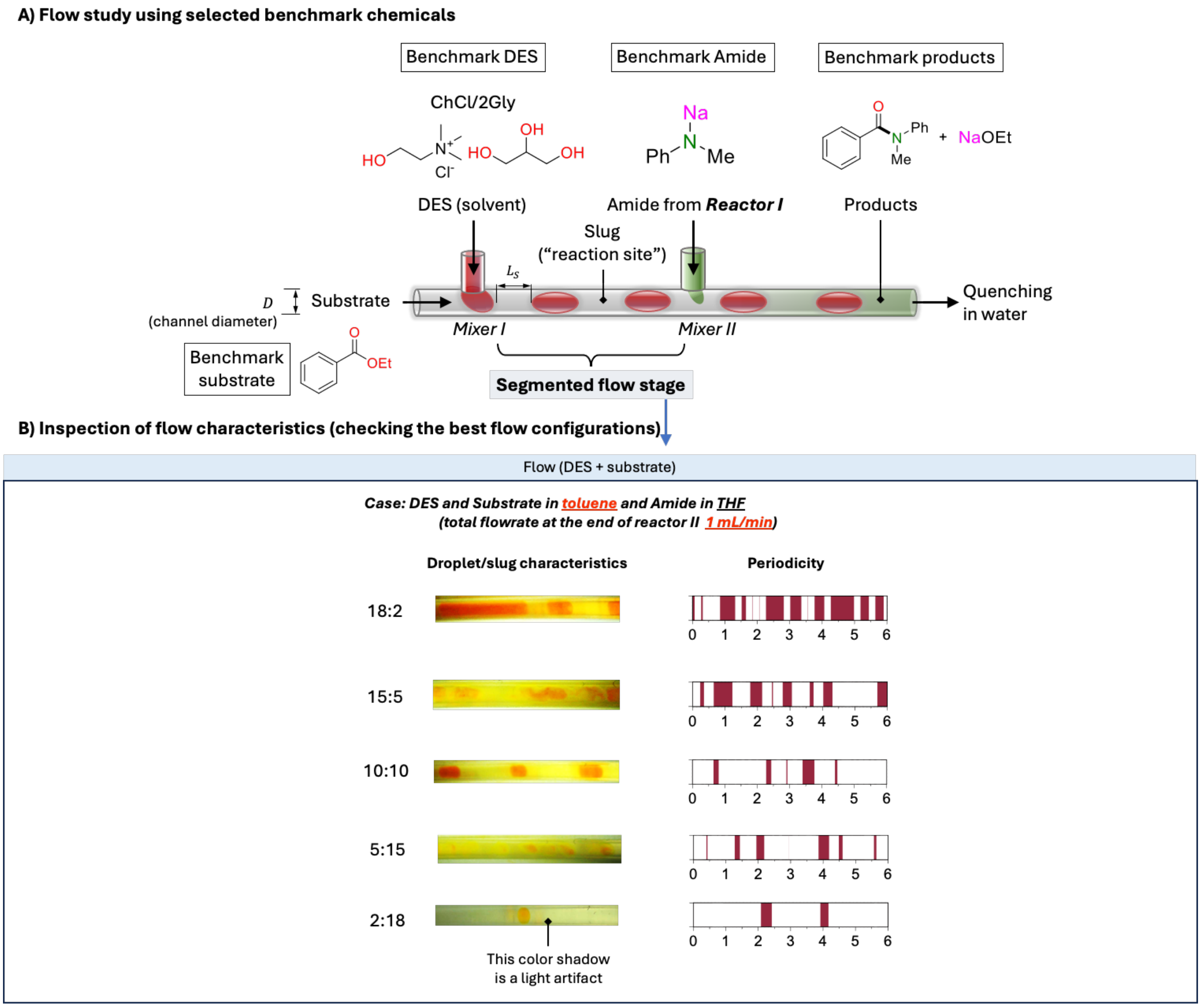

Figure S3: Visualization of the reactor at total flow rates of 1.0 ml/min with reaction (substrate in toluene and Amide in THF, coming from Reactor I).

Optimisation of the two phase flow

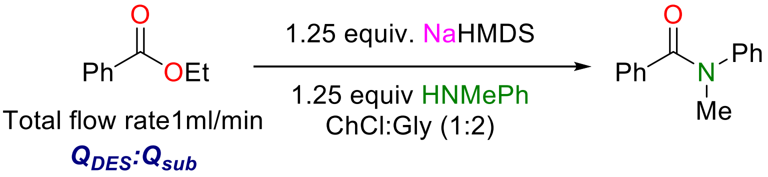

$Q_{sub}:Q_{DES}$	%Yield of **4a**
2:18	45
5:15	68
10:10	86
15:5	76
18:2	56

Table S6: effect of changing QDES/QSub on yield of 4a at total 1ml/min total flow rate

Optimisation of the total flow rate

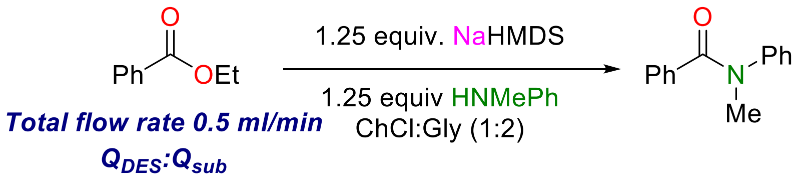

Q_{sub}:Q_{DES}	%Yield of 4a
2:18	54
5:15	34
10:10	64
15:5	13
18:2	9

Table S7: effect of changing QDES/QSub on yield of 4a at total 0.5ml/min total flow rate

Optimisation of DES

Studies were undertaken to evaluate the best DES for use in flow and to see if this differed from batch. As can be seen at high DES content ChC:Gly (1:2) performs best. It is also observed that in the case ChCl:H_2O low DES content appears to offer improved yields.

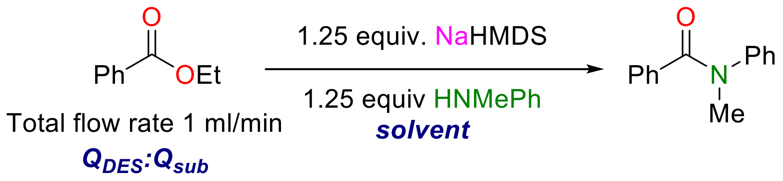

	Yield of 4a		
Q_{sub}:Q_{DES}	ChCl:EtGly (1:2)	ChCl:Gly (1:2)	ChCl:H_2O (1:2)
2:18	10	45	83
5:15	45	68	69
10:10	54	86	11
15:5	33	76	51
18:2	24	54	42

Table S8: effect of changing DES on yield of 4a at total 1ml/min total flow rate

Effect of water content on reactivity.

Previous reports have demonstrated that organolithium reagents are compatible with water under flow conditions . We have also observed that DES can interact with water to form a ternary system allowing large water content within reactor without clogging. We can observe that whilst no clogging occurs yields are significantly reduced by the prescence of water.

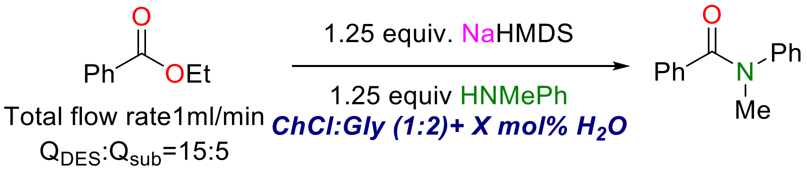

X mol% Water content	% Yield of 4a
10	53
25	36
50	29
100	clogging

Table S9: effect of water content on yield of 4a at total 1ml/min total flow rate

Optimisation of the residence time

To observe any effect that the DES might have in accelerating the reaction the residence time was reduced. This showed a significantly higher rate when using DES as opposed to using just toluene as a solvent. Whilst this may hint to DES playing a role in accelerating the reaction it cannot be discounted the effect that a biphasic system may have improved mixing.

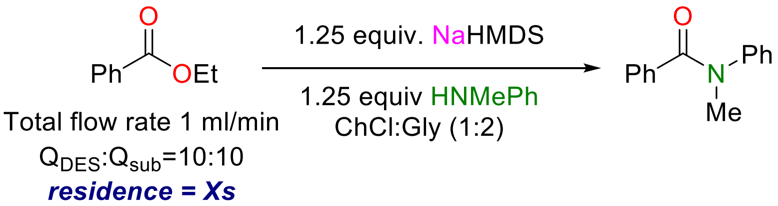

	%Yield 4a	
Residence (s)	10:10	toluene
1	63	37
2	71	44
3	80	57
4	84	63

Table S10: effect of changing residence time on yield of 4a

Quantification of the amine in each phase after reaction

To investigate the phase distribution of sodium amide during the reaction and its interaction with the deep eutectic solvent (DES) phase, the experiment was conducted without a substrate. Instead, toluene was injected into the DES mixture in place of the substrate. Following this, 2-iodoaniline, sourced from Reactor I, was introduced into the system. This amine was selected for its tracer properties (iodine) and was metalated in situ under consistent general conditions, varying the ratios of Q_{DES} to Q_{Sub}. The resulting product was collected in a

vial, allowing for the separation of the DES and organic phases. Both phases were then digested and diluted, and the iodide concentration required for ICP-MS analysis (in parts per billion) was monitored.

An increase in the amount of DES in the system (higher Q_{DES}/Q_{Sub} ratio) significantly enhances mass transfer between the organic phase and the DES phase, leading to a greater concentration of amine in the DES phase. This effect is primarily due to the heightened fluid recirculation frequency within the organic phase, which directly contributes to improved mass transfer dynamics. This conclusion is further substantiated by the accompanying table and figure. As the mass of amine/amide in the DES phase rises, the quantity available for reaction diminishes. Consequently, if a reaction were to occur under these conditions, the reactive substrate content of amine/amide would be lower with an increased amount of DES.

Q_{DES}:Q_{sub}	Molar fraction of amine in DES
18:2	0.142
10:10	0.026
5:15	0.013
2:18	0.064

Table S11: Effect of varying mass transfer on the molar fraction of amine (based in the elemental iodine content) present within the DES phase. The molar fraction represents the total proportion of amine in the entire system, encompassing both the organic and aqueous phases.

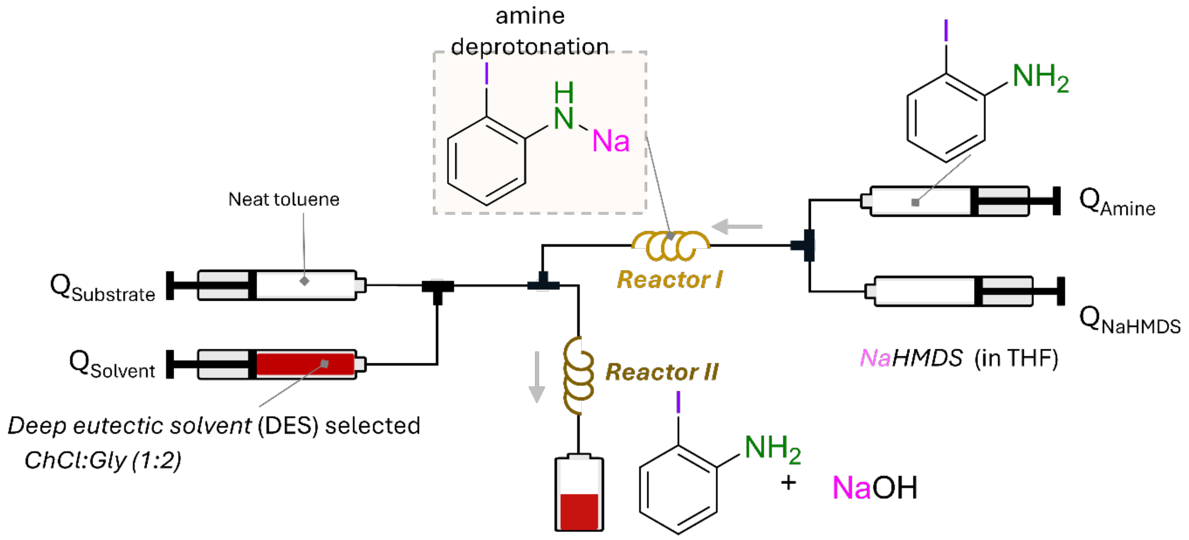

Figure S4: Schematic representation of the flow system utilized to investigate the impact of mass transfer. This example illustrates a Q_{DES} to Q_{Sub} ratio of 15:5.

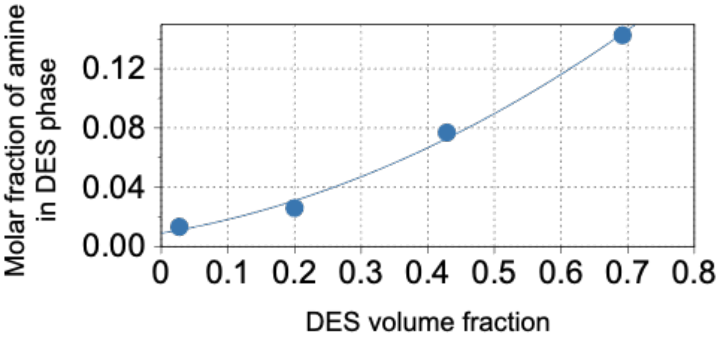

Figure S5: Representation of the quantity of amine (as indicated by the tracer element iodine) present in the DES phase, alongside the total amount of DES in the system. The molar fraction of amine in the DES phase reflects the proportion of the total amine present in the entire system, including both the aqueous and DES phases.

Clogging visualisation experiments

In a 4 dram vial commercial NaOH was added to ChCl:Gly (2 g) (1a) THF (2 ml) (1b). What can be seen is the formation of a suspension in DES forming small particles of NaOH. In THF we can see the product clumps to the bottom of the vial coating the bottom of the vial.

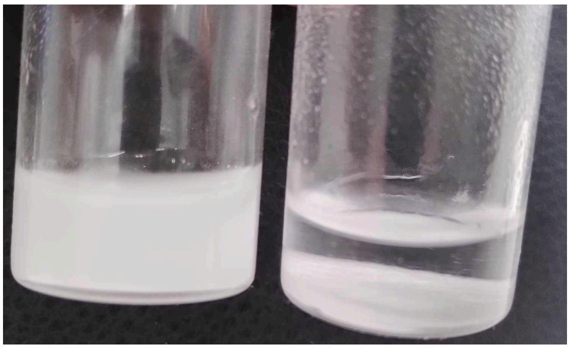

Figure S6: Comparison of NaOH in ChCl:Gly(1:2) (Left) and in THF (right)

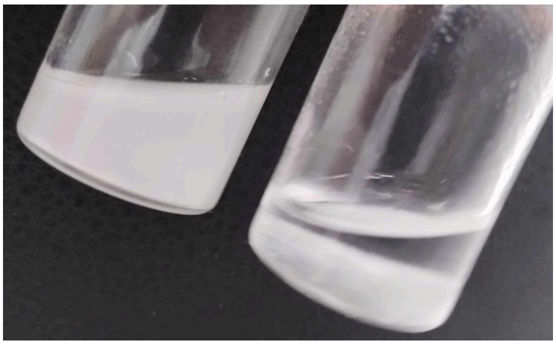

Figure S7: Comparison of NaF in ChCl:Gly(1:2) (Left) and in THF (right)

General flow conditions for scope

Chemical	Concentration of stock solutions (M)	Concentration (M)	Flow rate µL/min	Residence time reactor 1 (s)	Residence time reactor 2 (s)
Substrate	1.05	0.2625	142.86	128	41
Glyceline	-	-	428.57		
Amine	0.71	0.5250	334.82		
NaHMDS	2	2	93.75		

Table S12: General conditions used for the scope of the reaction

Ethyl acetate effect of changing Q_{Des}/Q_{Sub}

Low yields were observed when using ethyl acetate and ethyl trifluoroacetate. To understand this trend we changed the ratio of QDES/Qethylacetate this led to high yields at low DES flow rates and low yields at high DES flow rates. We believe this occurs due to greater solubility in the DES phase compared to other substrates.

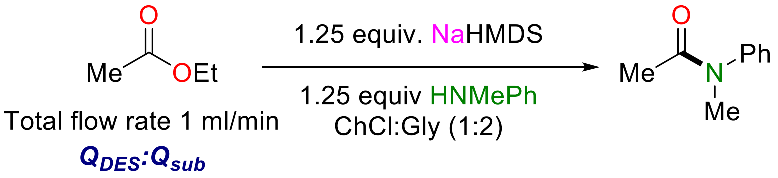

Flowrate Q_{DES}:Q_{Sub}	% Yield 4j
2:18	72
5:15	42
10:10	34
15:5	27
18:2	9

Table S13: effect of changing ratio of QDES/QSub on yield of 4j

Scale up reaction (10 mmol)

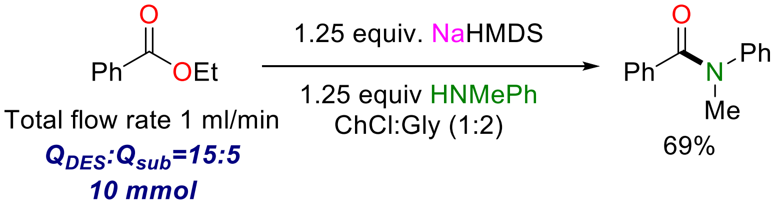

Reaction was performed according to the conditions in Tables S13 with larger 20 ml leur lock syringes used. The reactor was run for 67 minutes with no signs of clogging. Product was collected in a beaker filled with water (10 ml). The product was extracted with EtOAc (3x10 ml) and washed with 1M HCl (10 ml). The solution was dried using $MgSO_4$ and the residual solvent removed under vacuum leaving the crude product. Recrystalisation in hot hexane and storage at 0°C led to the isolation of **4a** as a white crystalline solid (1.46 g, 69%).

E-factor and atom economy for scale up of reaction

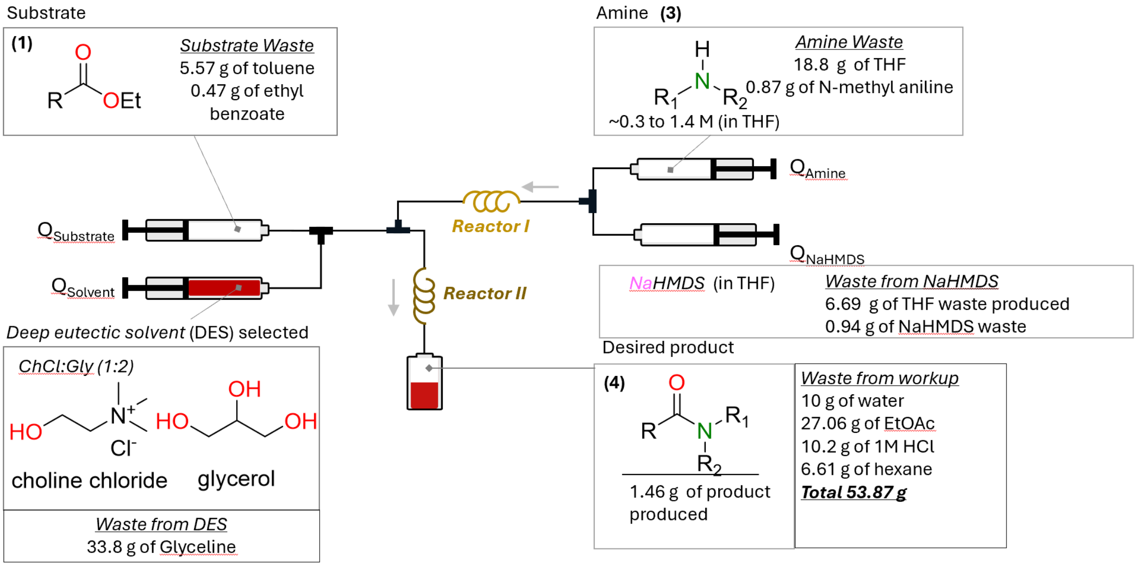

Total waste = 121.01 g
E -factor = 121.01/1.46 = 82.88

reagent waste = 2.28
Atom economy =1.46/ 2.28 x100 = 64.0%

Comparison with Szostak method for forming amides[7] (1g scale)

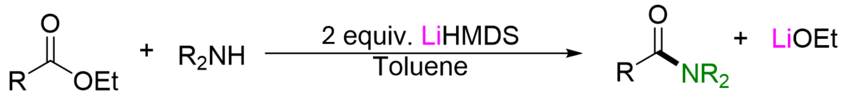

<u>Reaction waste</u>

Waste ester = 0.07 g

Waste amine = 0.198 g

Waste LiHMDS = 1.32 g

29.4 ml toluene = 25.48 g waste

14.7 ml THF = 13.05 g waste

<u>Work up waste</u>

40 ml NH_4Cl = 40 g waste

3x50 ml ethyl acetate = 135.3 g waste

100 ml HCl = 100 g waste

50 ml water = 50 g

50 ml brine = 50 g

Total waste = 414.41 g

Product formed 1.35 g

E-factor = 415.41/1.35 = 307.71

Reported spectroscopic data

N-methyl-N-phenylbenzamide (4a)

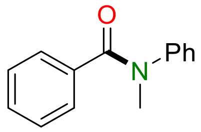

^{1}H NMR (500 MHz, $CDCl_3$): δ 7.31 (d, *J* = 7.8 Hz, 2H, Ar), 7.25-7.22 (m, 3H, Ar), 7.19-7.15 (m, 3H, Ar),7.06 (d, *J* = 7.8 Hz, 2H, Ar), 3.51 (s, 3H, CH_3).

$^{13}C\{^1H\}$ NMR (100 MHz, $CDCl_3$):δ 170.7 (CO),144.9 (Ar), 135.9 (Ar), 129.6 (Ar), 129.2 (Ar), 128.7 (Ar), 127.7 (Ar), 126.9 (Ar), 126.5 (Ar), 38.4(CH_3).

Spectroscopic data in agreement with literature [2]

Spectroscopic yield of 83%

4-Chloro-N-methyl-N-phenylbenzamide (4b)

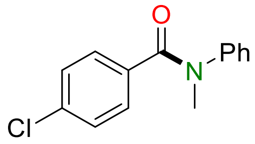

^{1}H NMR (500 MHz, $CDCl_3$): δ 7.33-7.23 (m, 3H, Ar), 7.23-7.15 (m, 4H, Ar), 7.01-6.93 (m, 2H, Ar), 3.47 (s, 3H, CH_3).

Spectroscopic data in agreement with literature [2]

Spectroscopic yield of 78%

4-bromo-N-methyl-N-phenylbenzamide (4c)

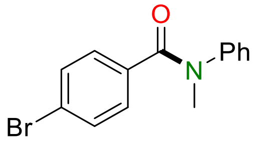

H NMR (500 MHz, $CDCl_3$): δ 7.33-7.22 (m, 4H, Ar), 7.19-7.15 (m, 3H, Ar), 7.03-7.01 (m, 2H, Ar), 3.49 (s, 3H, CH_3).

Spectroscopic yield of 80%

Spectroscopic data in agreement with literature [3]

3-iodo-N-methyl-N-phenylbenzamide (4d)

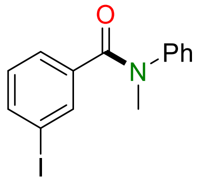

H NMR (500 MHz, $CDCl_3$): δ 7.72 (t, *J* = 1.7 Hz, 1H, Ar), 7.56 (d, *J* = 7.9 Hz, 1H, Ar), 7.33-7.12 (m, 4H, Ar), 7.09-6.99 (m, 2H, Ar), 6.87 (t, *J* = 7.8 Hz, 1H, Ar), 3.81 (s, 3H, CH_3).

$^{13}C\{^1H\}$ NMR (75.5 MHz, $CDCl_3$): δ168.7 (CO), 144.3 (Ar) ,138.4 (Ar),137.7(Ar), 137.6(Ar), 129.3(Ar),129.2(Ar), 127.6(Ar), 126.8 (Ar), 126.9 (Ar),93.3 (Ar), 38.3 (CH_3).

HRMS (ESI) m/z: $[M + H]^+$ calcd for $C_{14}H_{13}INO$ 338.0036 found 338.0039

Spectroscopic yield of 79%

4-methoxy-N-methyl-N-phenylbenzamide (4e)

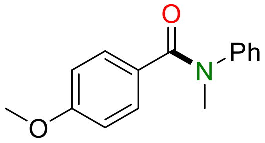

^1H NMR (500.1 MHz, $CDCl_3$): δ 7.28-7.21 (m, 4H, Ar),7.14 (t, J = 7.4 Hz, 1H, Ar), 7.07 (d, J = 7.4 Hz, 2H, Ar), 6.68 (d, J = 8.6 Hz, 2H Ar), 3.76 (s, 3H, OCH_3), 3.51(s,3H CH_3).

Spectroscopic yield of 83%

Spectroscopic data in agreement with literature [2]

N-methyl-N-phenylfuran-2-carboxamide (4f)

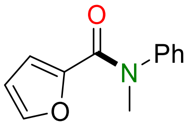

^1H NMR (500 MHz, $CDCl_3$): δ 7.45-7.35 (m, 3H, (CH/Ar), 7.34 (s, 1H, Ar), 7.25-7.22 (m, 2H, Ar), 6.23-6.20 (m, 1H, CH), 5.87-5.83 (m,1H, CH), 3.46 (s, 3H, CH_3).

Spectroscopic yield of 70%

Spectroscopic data in agreement with literature [3]

N-methyl-N-phenylnicotinamide (4g)

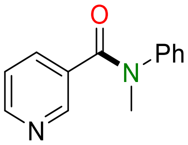

^1H NMR (500 MHz, $CDCl_3$): δ 8.50 (1H, d, J = 1.7 Hz, Ar), 8.46 (dd, J = 4.9, 1.6 Hz, 1H, Ar), 7.61 (dt, J = 7.9, 2.1 Hz, 1H, Ar), 7.24-7.05 (m, 3H, Ar), 7.10 (tt, J = 7.3, 2.0 Hz, 2H, Ar), 7.03 (dd, J = 8.0 Hz, 4.8 Hz, 1H, Ar), 6.62 (d, J = 7.6 Hz, 1H, Ar), 3.52 (s, 3H, CH_3).

Spectroscopic yield of 71%

Spectroscopic data in agreement with literature [2]

N-methyl-N-phenyl-2-naphthamide (4h)

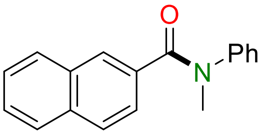

^{1}H NMR (500 MHz, $CDCl_3$): δ 7.89 (s, 1H, Ar), 7.71 (t, *J* = 6.1 Hz, 2H, Ar), 7.58 (d, *J* = 8.6 Hz, 1H, Ar), 7.47-7.40 (m, 2H), 7.32 (dd, *J* = 8.6, 1.7 Hz, 1H, Ar), 7.19 (t, *J* = 7.4 Hz, 2H, Ar), 7.12-7.07 (m,3H, Ar), 3.55 (s, 3H, CH_3).

Spectroscopic yield of 74%

Spectroscopic data in agreement with literature [3]

N-methyl-N-phenylpentanamide (4i)

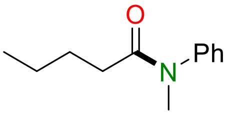

^{1}H NMR (500 MHz, $CDCl_3$): δ 7.41 (t, *J* = 7.6 Hz, 2H, Ar) 7.33 (t, *J* = 7.4 Hz, 1H, Ar) 7.17 (d, *J* = 7.7 Hz, 2H, Ar) 3.26 (s, 3H, CH_3) 2.06 (t, *J* = 7.6 Hz, 2H, CH_2) 1.61-1.52 (m, 2H, CH_2) 1.25-1.11 (m, 2H, CH_2) 0.82 (t, *J* = 7.0 Hz, 3H, CH_3).

Spectroscopic yield of 92%

Spectroscopic data in agreement with literature[4]

N-methyl-N-phenylacetamide (4j)

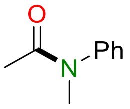

^{1}H NMR (500 MHz, $CDCl_3$): δ 7.42 (t, *J* = 7.3 Hz, 2H, Ar), 7.34 (t, *J* = 7.3 Hz, 1H, Ar), 7.19 (d, *J* = 7.4Hz, 2H, Ar), 3.27 (s, 3H, CH_3), 1.8 (s, 3H, CH_3).

Spectroscopic yield of 27%

Spectroscopic data in agreement with literature [5]

2,2,2-trifluoro-N-methyl-N-phenylacetamide (4k)

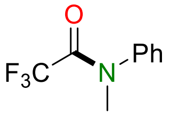

^{1}H NMR (500 MHz, $CDCl_3$): δ 7.39-7.18 (5H, m, Ar),3.28 (3H, s, CH_3).

^{19}F NMR (282.4 MHz, $CDCl_3$): δ -66.9 (s,CF_3)

Spectroscopic yield of 32%

Spectroscopic data in agreement with literature [6]

N-(4-(dimethylamino)phenyl)benzamide (4l)

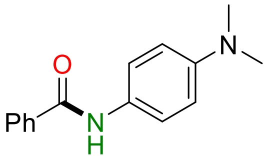

^{1}H NMR (500 MHz, $CDCl_3$): δ 7.87 (d, *J*=8.1 Hz, 2H,Ar), 7.42-7.57 (m, 5H, Ar), 7.75 (*J* = 9.04 Hz 2H, Ar) 2.95 (s, 6H, CH_3).

Spectroscopic yield of 67%

Spectroscopic data in agreement with literature [7]

N-(4-methoxyphenyl)benzamide (4m)

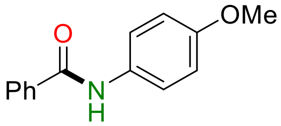

^{1}H NMR (500 MHz, $CDCl_3$): δ 7.90-7.85 (m, 3H, Ar), 7.76 (s, 1H, NH) 7.53-7.51 (m, 3H, Ar), 7.45 (t, J = 7.6 Hz,2H, Ar), 6.91 (d, J = 8.8 Hz, 2H, Ar), 3.79 (s, 3H, CH_3).

Spectroscopic yield of 71%

Spectroscopic data in agreement with literature [7]

N-(benzo[d][1,3]dioxol-5-yl)benzamide (4n)

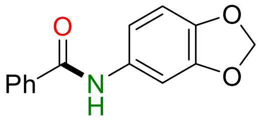

^{1}H NMR (500 MHz, $CDCl_3$): δ 7.86-7.81 (m, 2H, Ar), 7.77 (br, 1H, NH), 7.57-7.50 (m, 1H, Ar), 7.49-7.43 (m, 2H, Ar), 7.37-7.32 (m, 1H, Ar), 6.92-6.87 (m, 1H, Ar), 6.80-6.74 (m,1H, Ar), 5.96 (s, 2H, CH_2)

Spectroscopic yield of 77%

Spectroscopic data in agreement with literature [4]

N-(2-iodophenyl)benzamide (4o)

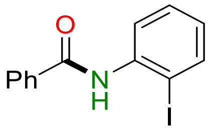

^{1}H NMR (500 MHz, $CDCl_3$): δ 8.48 (dd, *J*=8.2, 1.6, 1H, Ar) 8.32 (s, 1H, NH) 8.01-7.96 (m, 2H, Ar) 7.84 (dd, *J*=8.0, 1.5, 1H,Ar) 7.66-7.52 (m, 3H, Ar) 7.48-7.32 (m, 1H, Ar) 6.89 (t, *J* =, 7.5, 1H, Ar)

Spectroscopic yield of 76%

Spectroscopic data in agreement with literature [9]

N-mesitylbenzamide (4p)

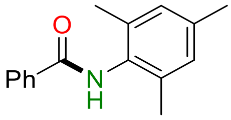

^{1}H NMR (500 MHz, $CDCl_3$) δ 7.95-7.87 (m, 2H, Ar), 7.59-7.53 (m, 1H, Ar), 7.52-7.46 (m,2H, Ar), 7.28 (br s, 1H, NH), 6.96 (s, 2H, Ar), 2.32 (s, 3H, CH_3), 2.27 (s, 6H, CH_3).

Spectroscopic yield of 54%

Spectroscopic data in agreement with literature [10]

morpholino(phenyl)methanone (4q)

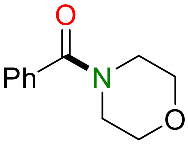

^{1}H NMR (500 MHz, $CDCl_3$) 7.45-7.32 (5H, m, Ar), 3.90-3.36 (m,8H CH_2)ppm

Spectroscopic yield of 83%

Spectroscopic data in agreement with literature [2]

(3,4-dihydroquinolin-1(2H)-yl)(phenyl)methanone (4r)

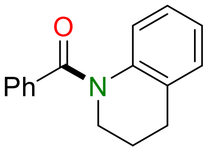

^{1}H NMR (500 MHz, $CDCl_3$): δ 7.39-7.31 (m, 3H, Ar) 7.27 (t, J = 7.5 Hz, 2H, Ar) 7.15 (d, J = 7.5 Hz, 1H, Ar) 6.99 (t, J = 7.4 Hz, 1H, Ar) 6.86 (t, J = 7.7 Hz, 1H, Ar) 6.73 (s, 1H,Ar) 3.91 (t, J = 6.5 Hz, 2H, CH_2) 2.85 (t, J = 6.6 Hz, 2H, CH_2) 2.05 (p, J = 6.6 Hz, 2H, CH_2)

Spectroscopic yield of 90%

Spectroscopic data in agreement with literature [7]

6-fluoro-N-methyl-N-phenylpyridin-2-amine (5a)

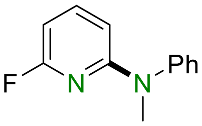

^{1}H NMR (300 MHz, $CDCl_3$): δ 7.48 (q, J = 8.1 Hz, 1H, Ar) 7.23 (d, J = 8.9 Hz, 2H, Ar) 6.90 (d, J = 8.9 Hz, 2H, Ar) 6.42 (s,1H, Ar) 6.46 (dd, J = 8.1, 2.4 Hz, 1H, Ar) 6.24 (dd, J = 7.8, 2.4 Hz, 1H, Ar) 3.81 (s 3H, CH_3)

$^{13}C\{^{1}H\}$ NMR (75.5 MHz, $CDCl_3$): δ 163.1(d, J_{C-F} = 237.8 Hz, (CF) ,156.8 (Ar),142.0 (d J_{C-F} = 8.3, Ar),132.3 (Ar), 124.6 (Ar), 114.7(Ar),102.9 (Ar), 97.4 (d, J_{C-F} = 36.5 Hz,Ar),55.5 (CH_3)

^{19}**F NMR** (282.4 MHz, $CDCl_3$): δ -69.3 (s, CF)

HRMS (ESI) m/z: $[M + H]^+$ calcd for $C_{12}H_{12}FN_2$ 203.0979 found 203.0980

Spectroscopic yield of 82%

6-fluoro-N-(4-methoxyphenyl)pyridin-2-amine (5b)

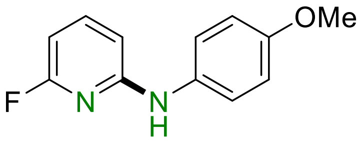

^{1}**H NMR** (300 MHz, $CDCl_3$): δ 7.50 (q, J = 8.1 Hz, 1H, Ar), 6.89 (d, J = 2.1 Hz, 1H, Ar) 6.79 (d, J = 8.2 Hz, 1H, Ar) 6.71 (dd, J = 8.3, 2.1 Hz, 1H, Ar) 6.49 (dd, J = 8.0, 2.3 Hz, 1H, Ar) 6.36 (s, 1H, NH) 6.27 (dd, J = 7.7, 2.4 Hz, 1H, Ar) 3.84 (s, 3H, CH_3)

^{13}**C{^{1}H} NMR** (75.5 MHz, $CDCl_3$): δ 163.1 (d $J_{C\text{-}F}$ 237.6 Hz, CF), 161.5 (Ar), 156.7 (Ar),142.0 (d $J_{C\text{-}F}$ 8.71 Hz, Ar), 132.3(Ar), 124.6 (Ar),114.6 (Ar),102.9 (d J=3.73 Hz, Ar), 97.3 (d $J_{C\text{-}F}$ =36.1 Hz, Ar), 55.5 (Ar)

^{19}**F NMR** (282.4 MHz, $CDCl_3$): δ -69.4 (s, CF)

HRMS (ESI) m/z: $[M + H]^+$ Calcd for $C_{12}H_{12}FN_2O$ 219.0928 found 219.0927

Spectroscopic yield of 79%

N-(benzo[d][1,3]dioxol-5-yl)-6-fluoropyridin-2-amine (5c)

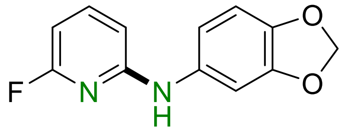

^{1}**H NMR** (300 MHz, $CDCl_3$): δ 7.50 (q, J = 8.1,Hz, 2H, Ar), 6.89 (d, J = 2.1 Hz, 1H, Ar), 6.79 (d, J=8.2, 1H, Ar), 6.71 (dd, J=8.3, 2.1, 1H, Ar) 6.49 (dd, J=8.1, 2.3, 1H, Ar),6.35 (s, 1H,NH) 6.26 (dd, J=7.7, 2.4, 1H, Ar), 5.98 (s, 2H, CH_2)

^{13}**C{^{1}H} NMR** (75.5 MHz, $CDCl_3$,): δ 164.6, (d, $J_{C\text{-}F}$ = 237 Hz, CF), 156.1 (Ar),148.2 (Ar),144.6 (Ar), 142.0 (d, $J_{C\text{-}F}$ =8.3 Hz, Ar),133.5(Ar),115.9 (Ar),108.5 (Ar) ,104.9 (Ar),103.3 (Ar),101.4 (CH_2), 97.7(d, $J_{C\text{-}F}$ = 37.0 Hz,Ar).

^{19}**F NMR** (282.4 MHz, $CDCl_3$): δ -69.3 (s, CF).

HRMS (ESI) m/z: $[M + H]^+$ Calcd for $C_{12}H_{10}FN_2O_2$ 233.0721 found 233.0723

Spectroscopic yield of 75%

1-(6-fluoropyridin-2-yl)-1,2,3,4-tetrahydroquinoline (5d)

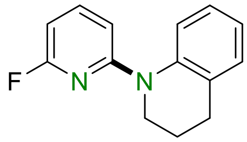

^{1}H NMR (300 MHz, $CDCl_3$) δ 7.48 (q, J = 8.1,Hz, 2H, Ar), 7.38 (d, J = 8.2 Hz, 1H, Ar),7.18-7.10 (m, 2H, Ar),7.01-6.96(m, 2H, Ar),(dd) 3.92(t,J=6.1 Hz 2H,CH_2)2.78 (t,J=6.5 Hz 2H, CH_2) 1.99 (q, 6.4Hz, 2H CH_2)

^{13}C{^{1}H} NMR (75.5 MHz, $CDCl_3$,):δ 162.9,(d J_{C-F} = 236 Hz, CF) 161.4 (Ar), 157.0 (Ar),141.1(d,J=8.3 Hz, Ar) 140.6 (Ar), 131.0 (Ar),129.3 (Ar),126.1(Ar),122.9 (Ar),121.4 (Ar) 107.1(Ar), 98.0(d,J=37.0 Hz, Ar) 45.9 (CH_2), 27.4 (CH_2), 23.8 (CH_2).

^{19}F NMR (282.4 MHz, $CDCl_3$): δ -68.42 (s, CF)

HRMS (ESI) m/z: $[M + H]^+$ Calcd $C_{14}H_{14}FN_2$ 229.1136 found 229.1134

Spectroscopic yield of 46%

Sodium tetrahydroquinolide-$[Na(THQ)THF]_2$ (6)

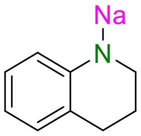

To a Schlenk ^{n}BuNa (160 mg 2 mmol) was suspended in hexane (15 ml) the reaction was cooled to 0°C and Tetrahydroquiniline (0.260 ml 2 mmol) was added dropwise the reaction was stirred for 1h before after which THF (3 ml) was added and they yellow solution stored at -15°c overnight leading to formation yellow blocks (430 mg,72%).

H NMR (300 MHz, THF-d^8): δ 6.45 (td, *J* = 5.5, 1.9 Hz, 1H, Ar) 6.39-6.34 (m, 1H, Ar) 5.92 (dd, *J* = 8.1, 1.2 Hz, 1H, Ar) 5.60 (td, *J* = 7.0, 1.3 Hz, 1H, Ar) 3.65-3.59 (m, 2H, THF) 3.49 (t, *J* = 4.8 Hz, 2H, CH_2) 2.63 (t, *J* = 6.3 Hz, 2H, CH_2) 1.83-1.74 (m, 2H, THF) 1.74-1.63 (m, 2H, CH_2)

^{13}C{^{1}H} NMR (75.5 MHz, THF-d^8,): δ 161.5 (Ar_{ipso}), 130.0 (Ar), 127.5 (Ar), 199.1 (Ar), 114.0 (Ar), 105.6 (Ar), 68.3 (THF) ,50.9 (CH2), 31.3 (CH_2) ,26.5 (THF) ,25.74 (CH2)

CHN calculated C_{26} H_{36} N_2 O_2 Na_2 68.7 C, 7.98 H,6.16N found C 68.42, 7.63H, 6.59N

Sodium morphilide $[Na(Morph)(THF)]_\infty$ (7)

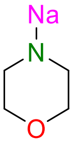

To a Schlenk ^{n}BuNa (160 mg, 2 mmol) was suspended in pentane (15 ml) the reaction was cooled to -78°C and Morpholine (0.180 ml 2 mmol) was added dropwise the reaction was stirred for 1h before stirring at room temperature for an additional hour. THF (10 ml) was

added, and the solvent reduced by half leading to the formation of a yellow solution. Storage at -15°c overnight led to the formation of colourless crystals (130 mg, 36%)

^{1}H NMR (300 MHz, THF-d^{8}) : δ 3.69-3.59 (m, 4H, THF) 3.27 (t, *J* = 3.7 Hz, 4H,CH_2N) 3.09 (t, *J* =4.8 Hz, 4H, CH_2O) 1.84-1.78 (THF) (m 4H)

^{13}C{^{1}H} NMR (75.5 MHz, THF-d^{8},): δ 71.3(CH_2N)**,** 68.3(THF), 53.8, (CH_2O) 26.4 (THF)**.**

Despite several attempts, no meaningful elemental analysis could be carried out on the crystals due to extremely high temperature sensitivity.

NMR Spectra

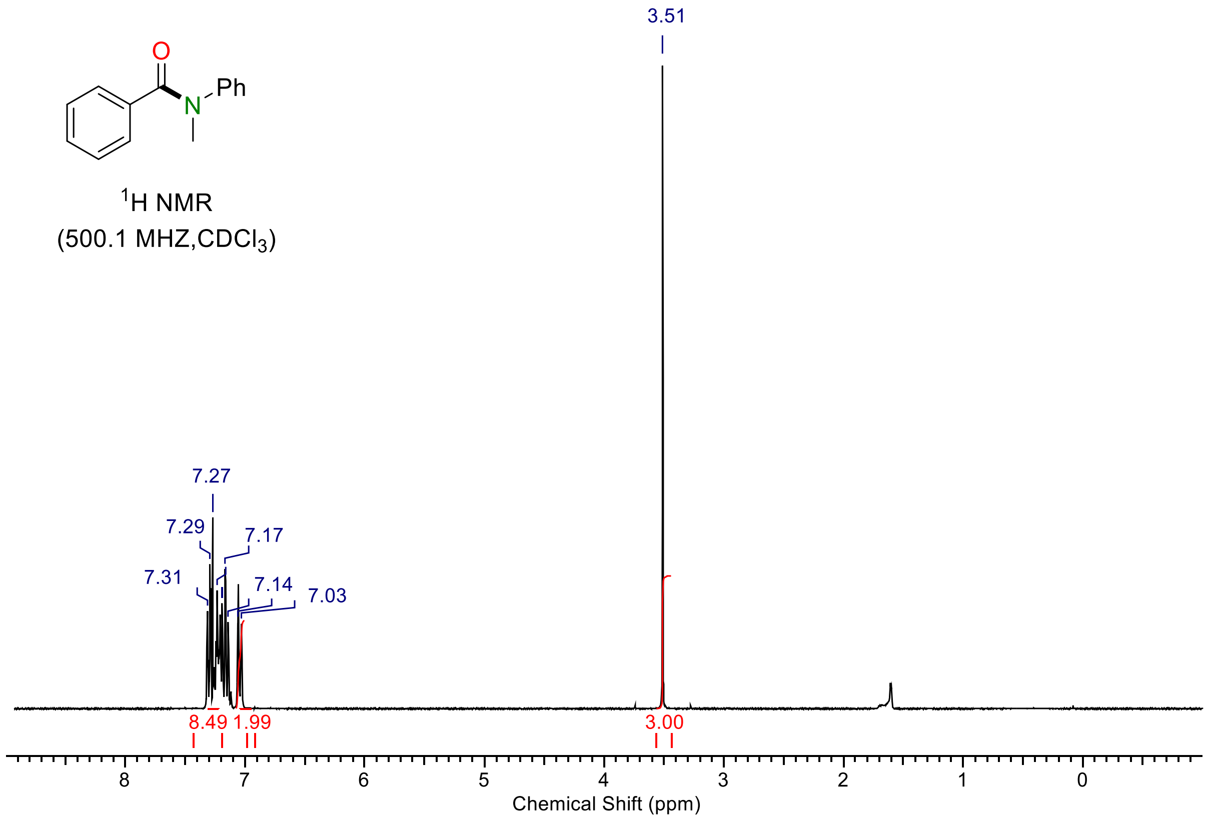

Spectra S1: 1H NMR spectrum of isolated 4a

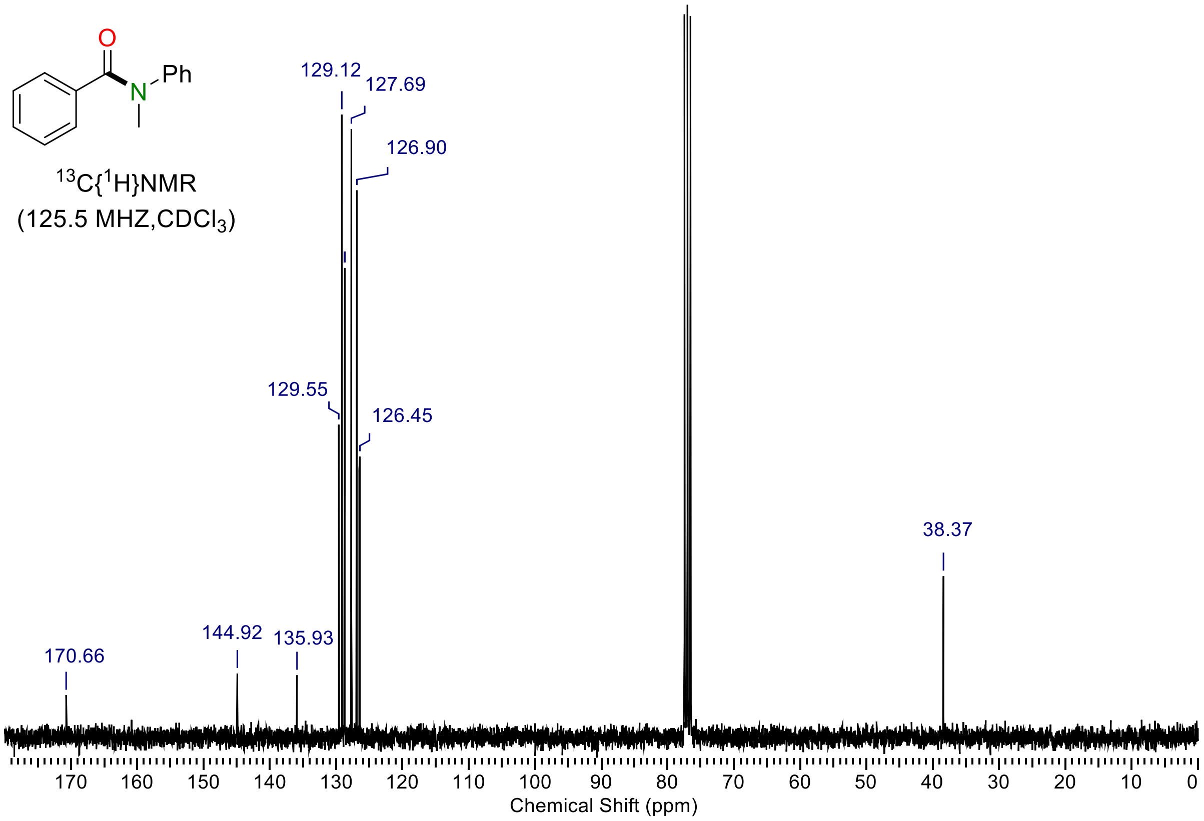

Spectra S2: 13C(1H) NMR spectrum of isolated 4a

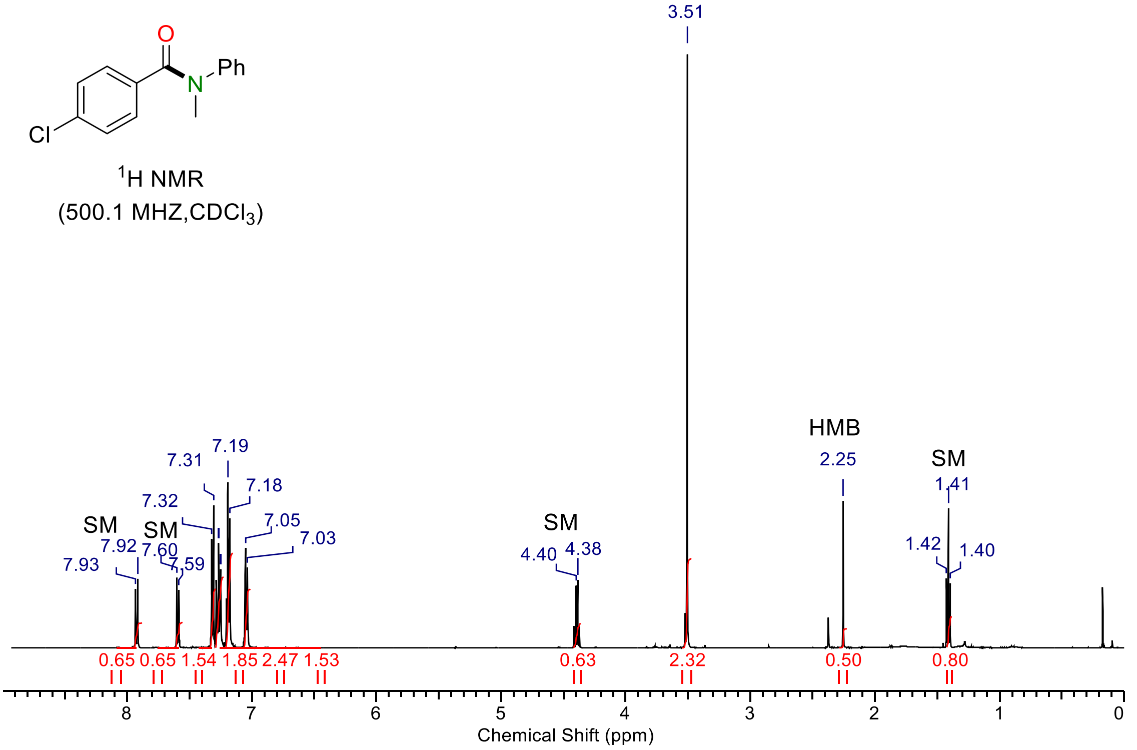

Spectra S3: ^{1}H NMR spectrum of synthesised 4b with Hexamethylbenzene as an internal standard

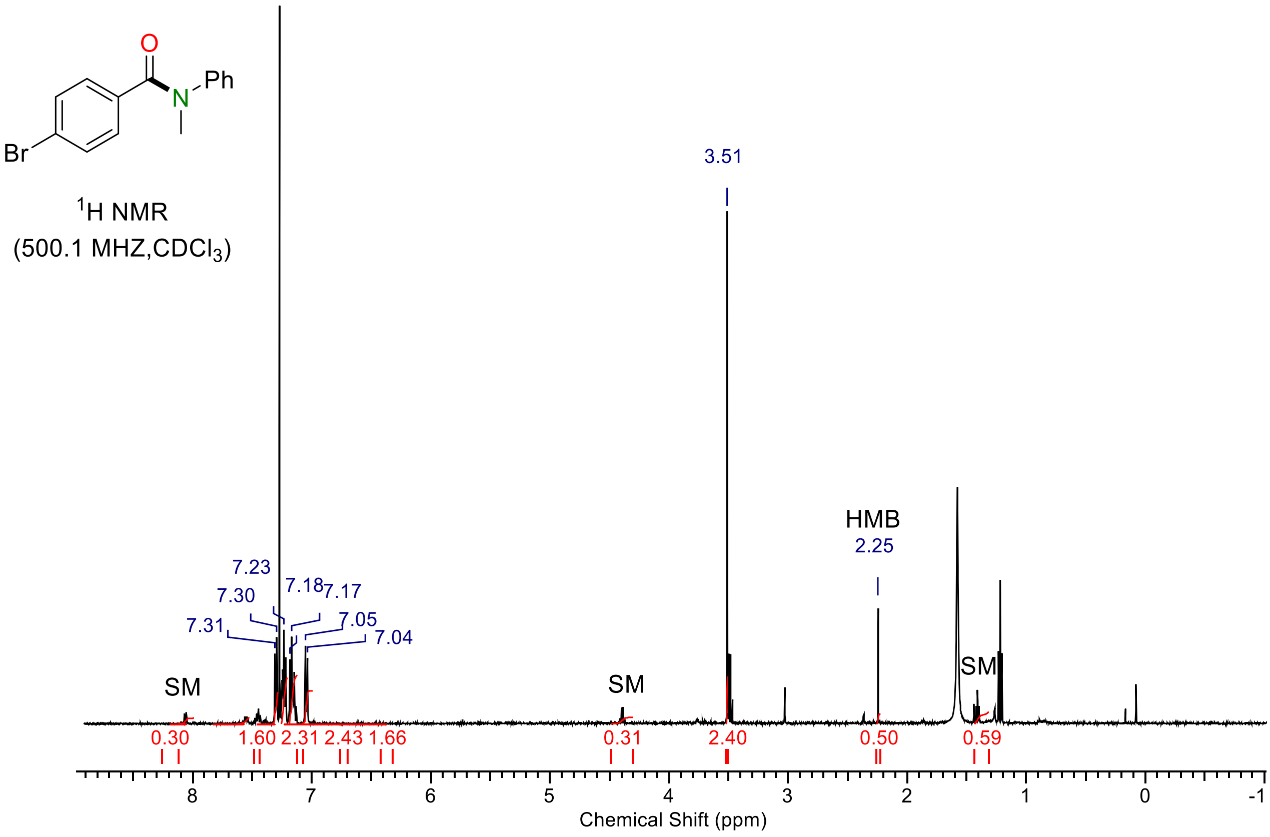

Spectra S4: ^{1}H NMR spectrum of synthesised 4c with Hexamethylbenzene as an internal standard

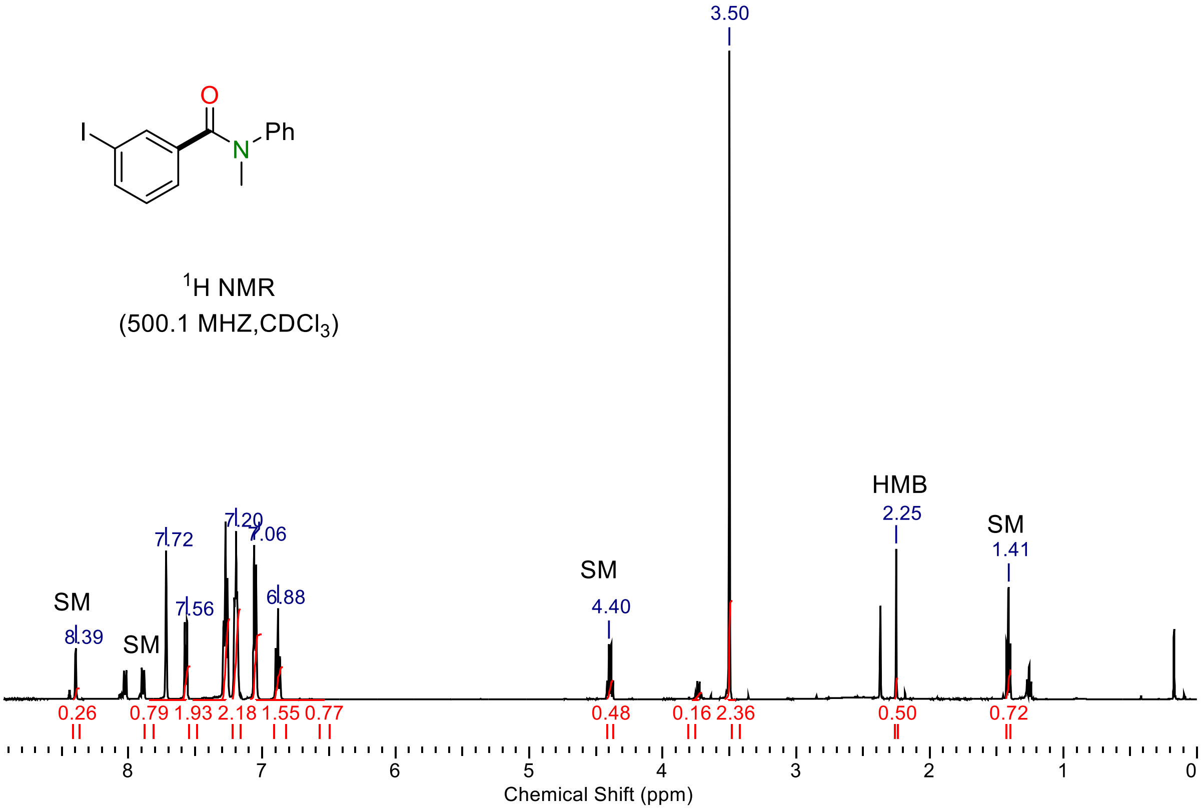

Spectra S5: ^{1}H NMR spectrum of synthesised 4d with Hexamethylbenzene as an internal standard

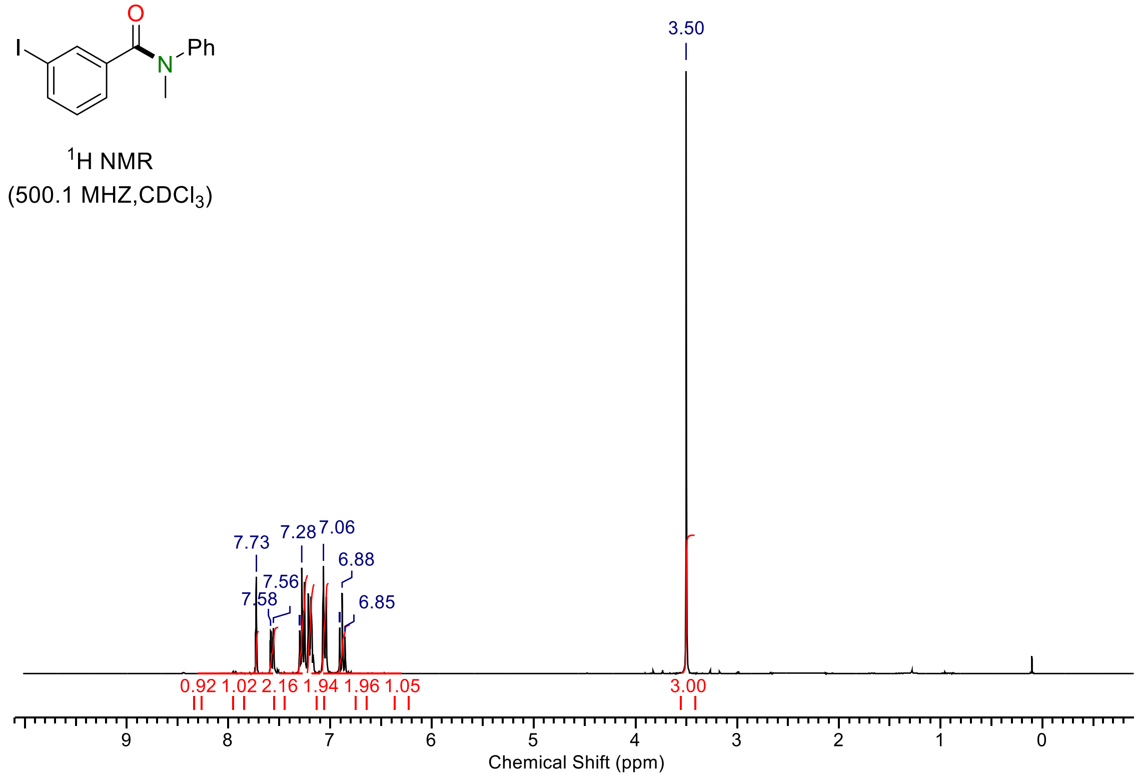

Spectra S6: ^{1}H NMR spectrum of isolated 4d

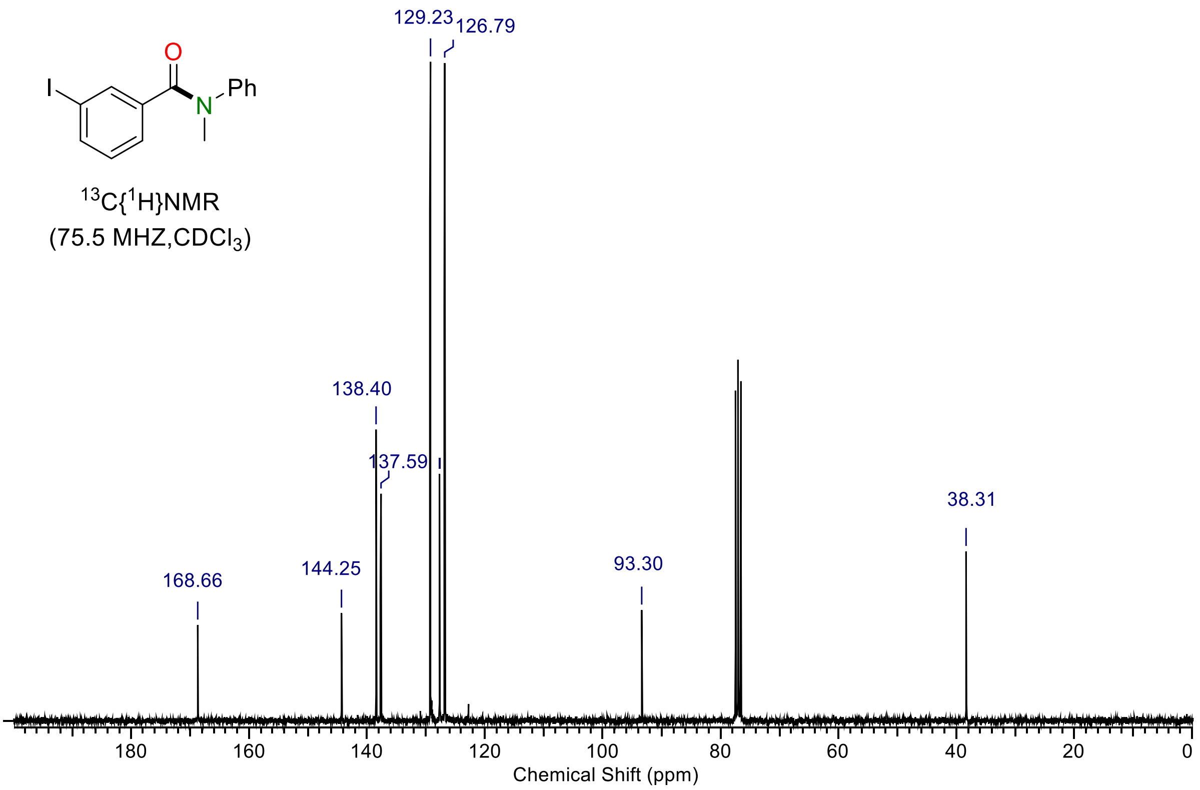

Spectra S7: $^{13}C(^1H)$ NMR spectrum of isolated 4d

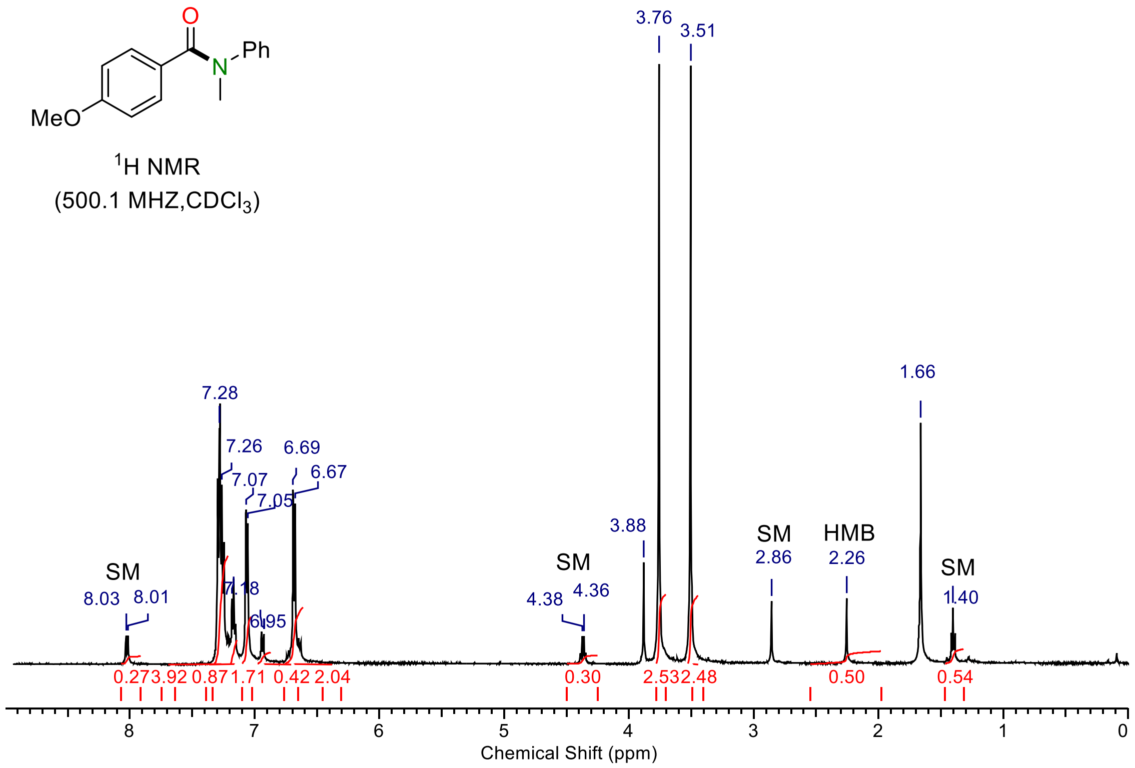

Spectra S8: ^1H NMR spectrum of synthesised 4e with Hexamethylbenzene as an internal standard

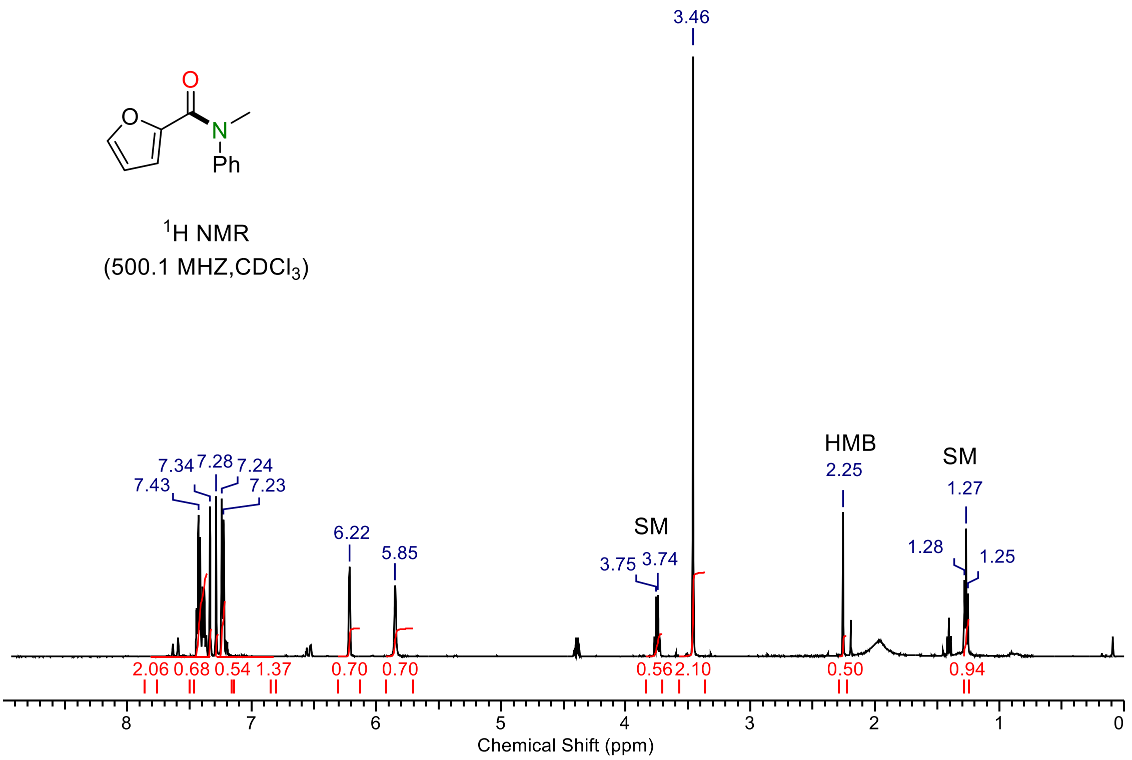

Spectra S9: ^{1}H NMR spectrum of synthesised 4f with Hexamethylbenzene as an internal standard

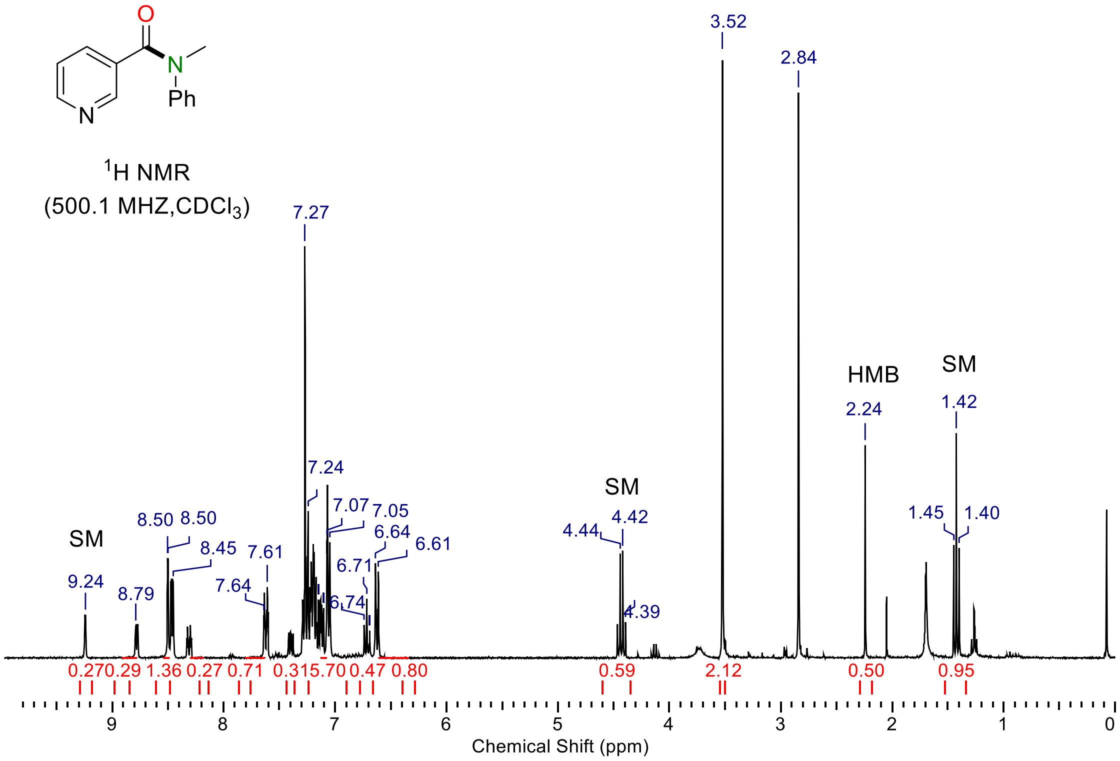

Spectra S10: ^{1}H NMR spectrum of synthesised 4g with Hexamethylbenzene as an internal standard

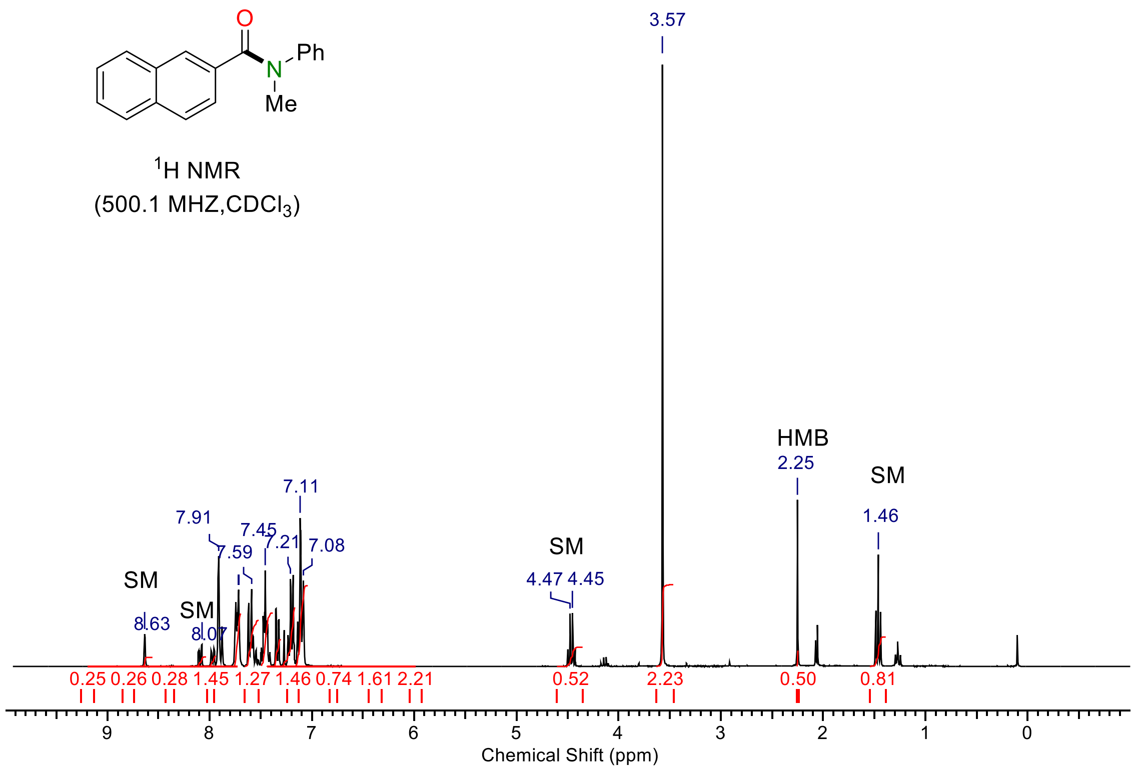

Spectra S11: ¹H NMR spectrum of synthesised 4h with Hexamethylbenzene as an internal standard

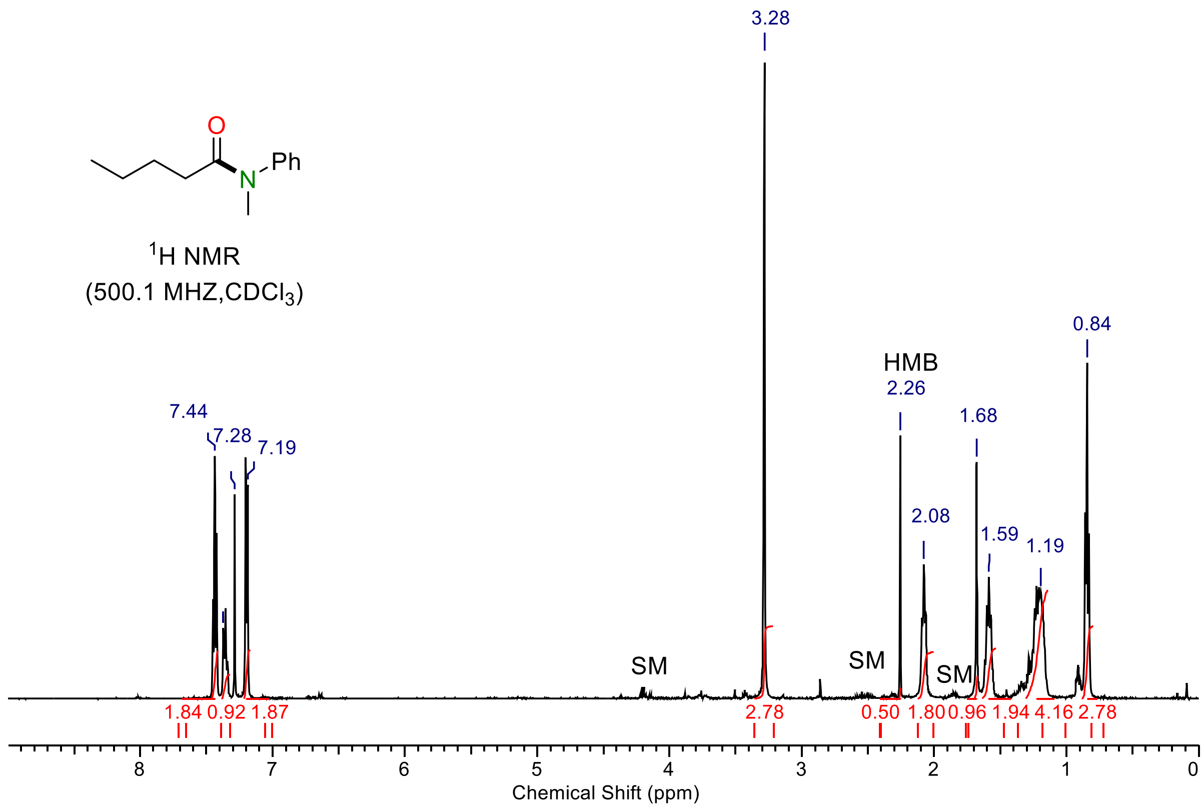

Spectra S12: ¹H NMR spectrum of synthesised 4i with Hexamethylbenzene as an internal standard

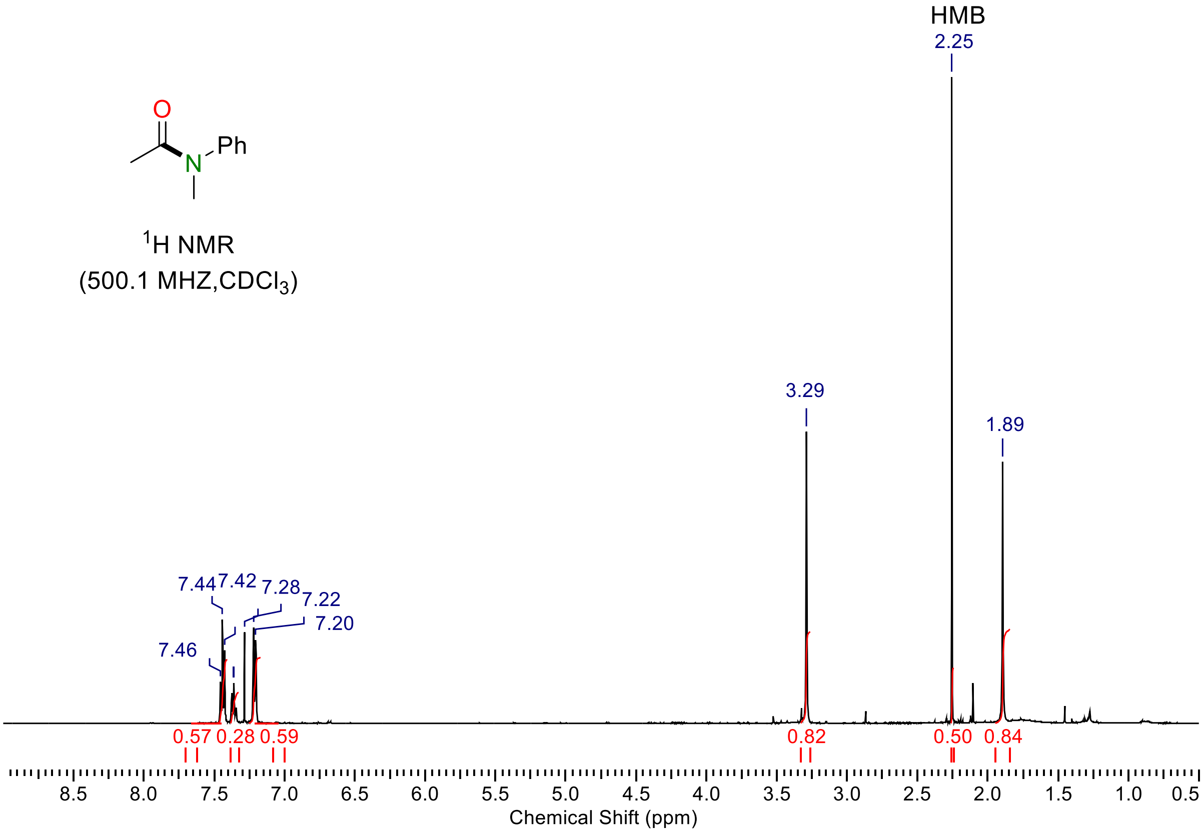

Spectra S13: ^{1}H NMR spectrum of synthesised 4j with Hexamethylbenzene as an internal standard

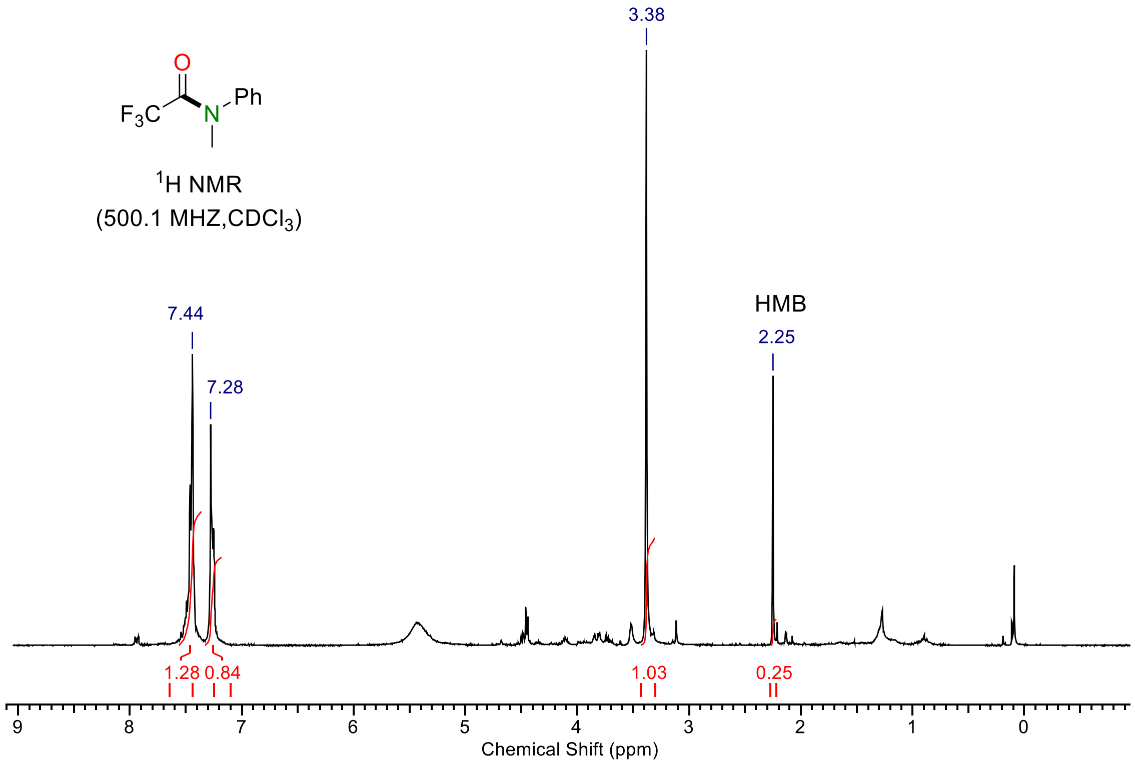

Spectra S14: ^{1}H NMR spectrum of synthesised 4k with Hexamethylbenzene as an internal standard

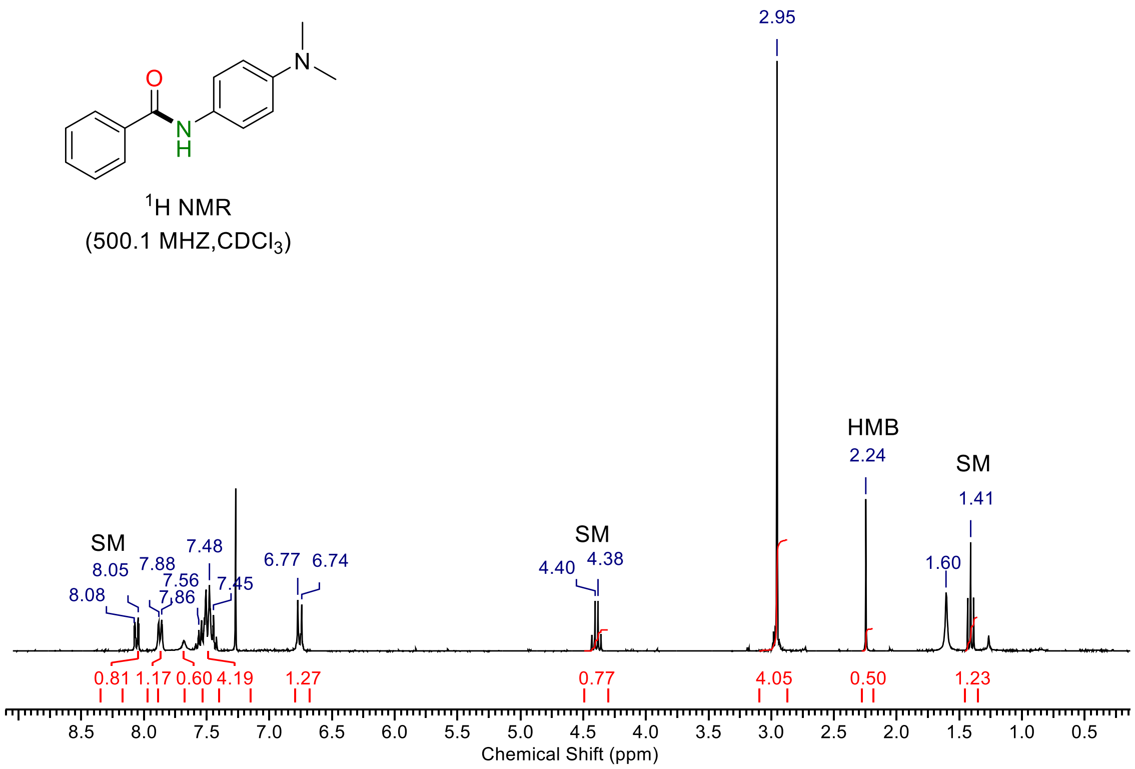

Spectra S15: ^{1}H NMR spectrum of synthesised 4l with Hexamethylbenzene as an internal standard

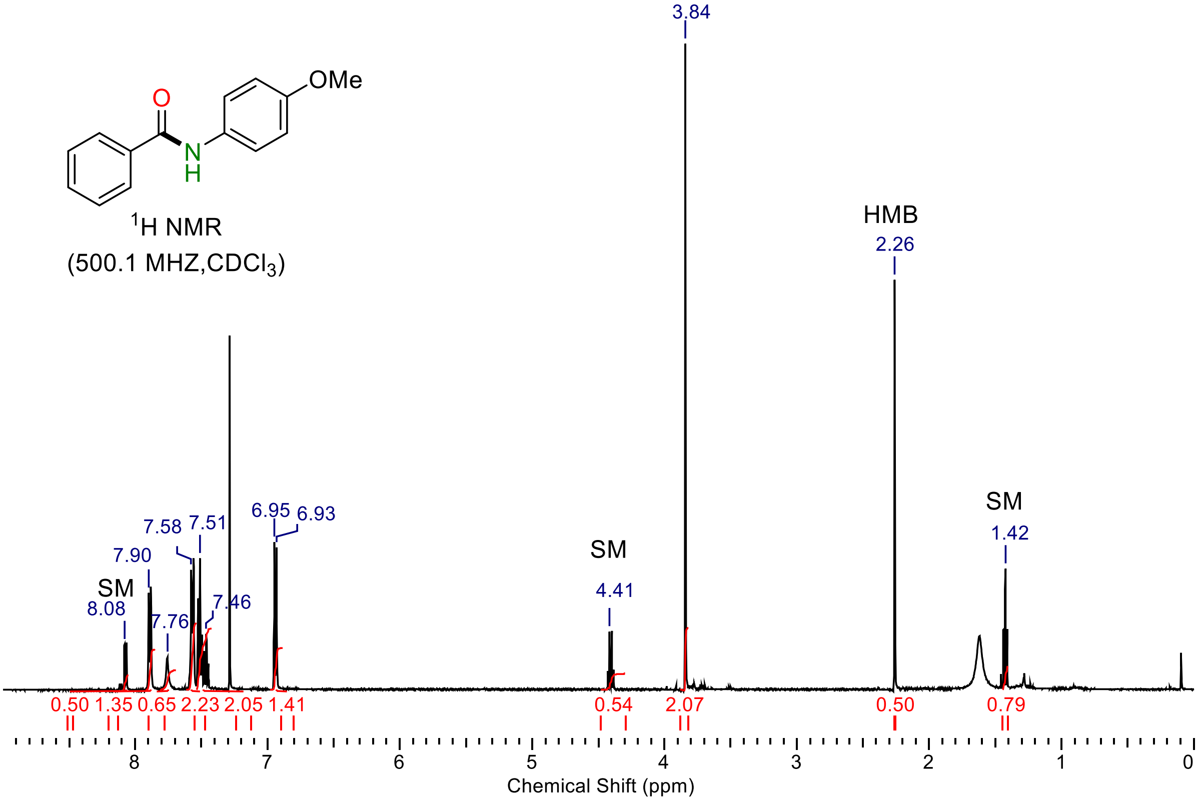

Spectra S16: ^{1}H NMR spectrum of synthesised 4m with Hexamethylbenzene as an internal standard

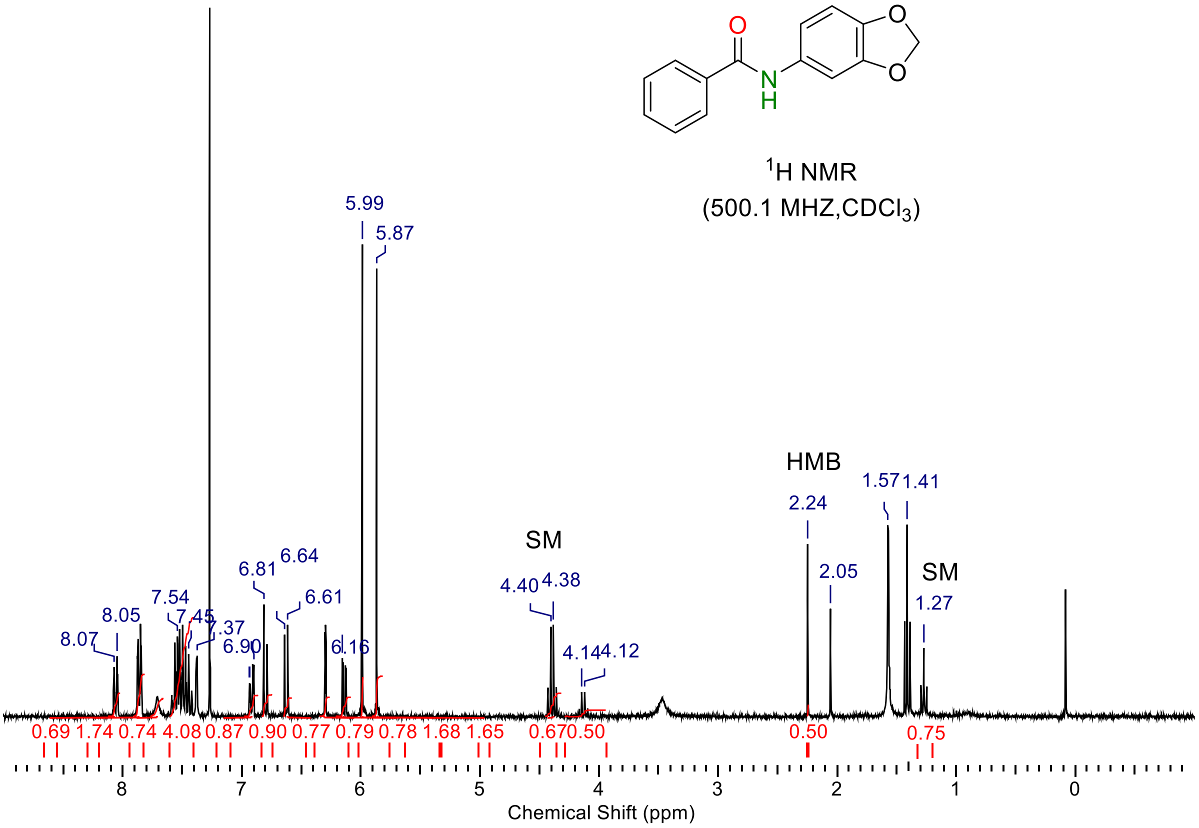

Spectra S17: ¹H NMR spectrum of synthesised 4n with Hexamethylbenzene as an internal standard

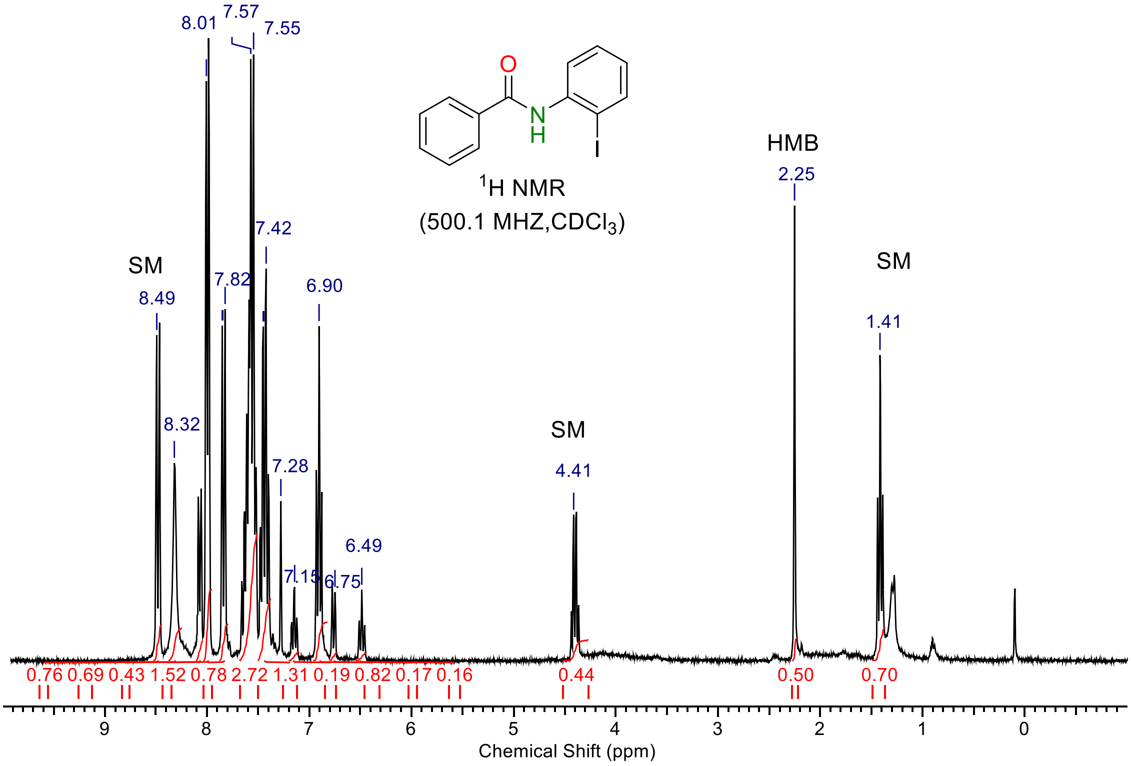

Spectra S18: ¹H NMR spectrum of synthesised 4o with Hexamethylbenzene as an internal standard

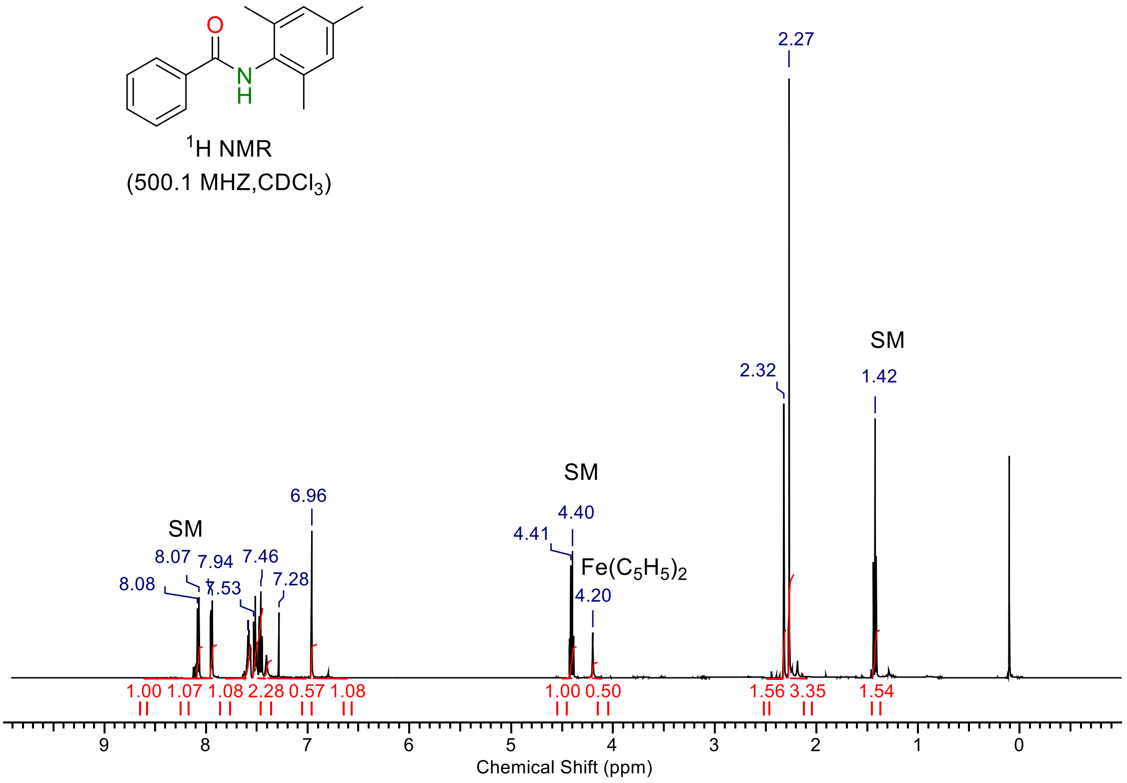

Spectra S19: ¹H NMR spectrum of synthesised 4p with Ferrocene as an internal standard

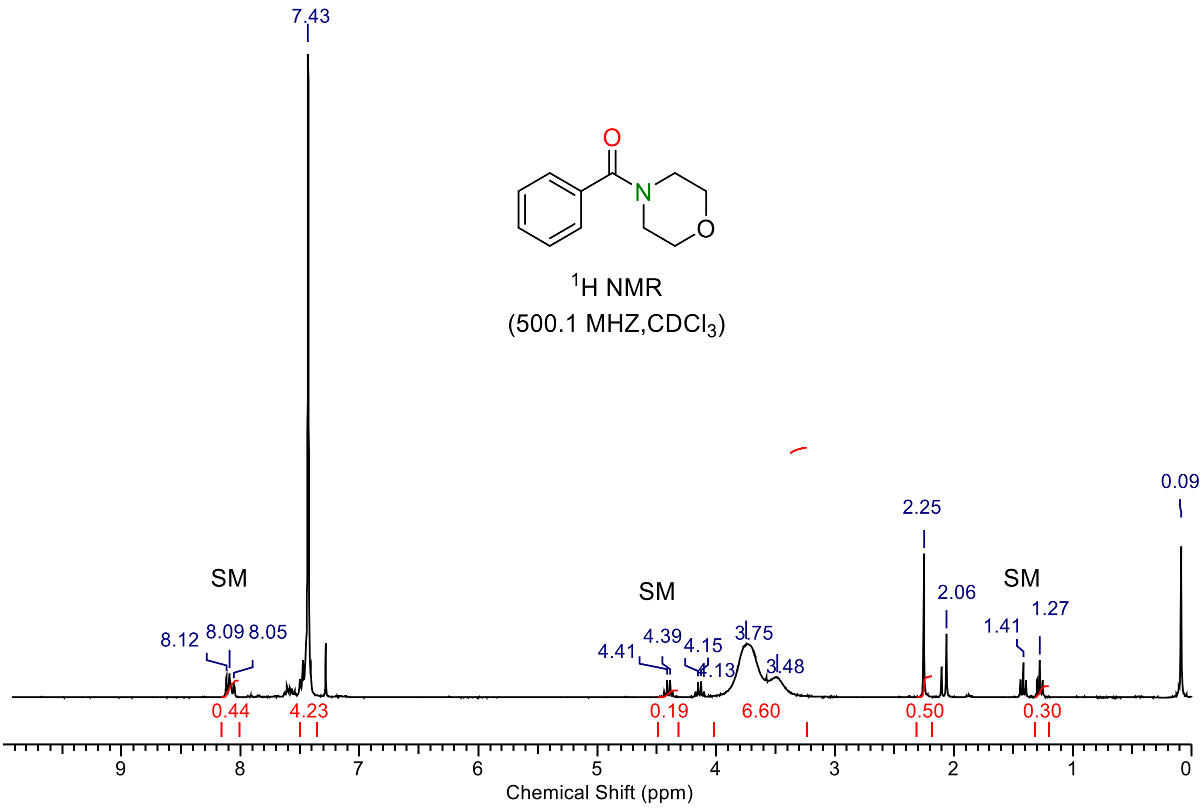

Spectra S20: ¹H NMR spectrum of synthesised 4q with Hexamethylbenzene as an internal standard

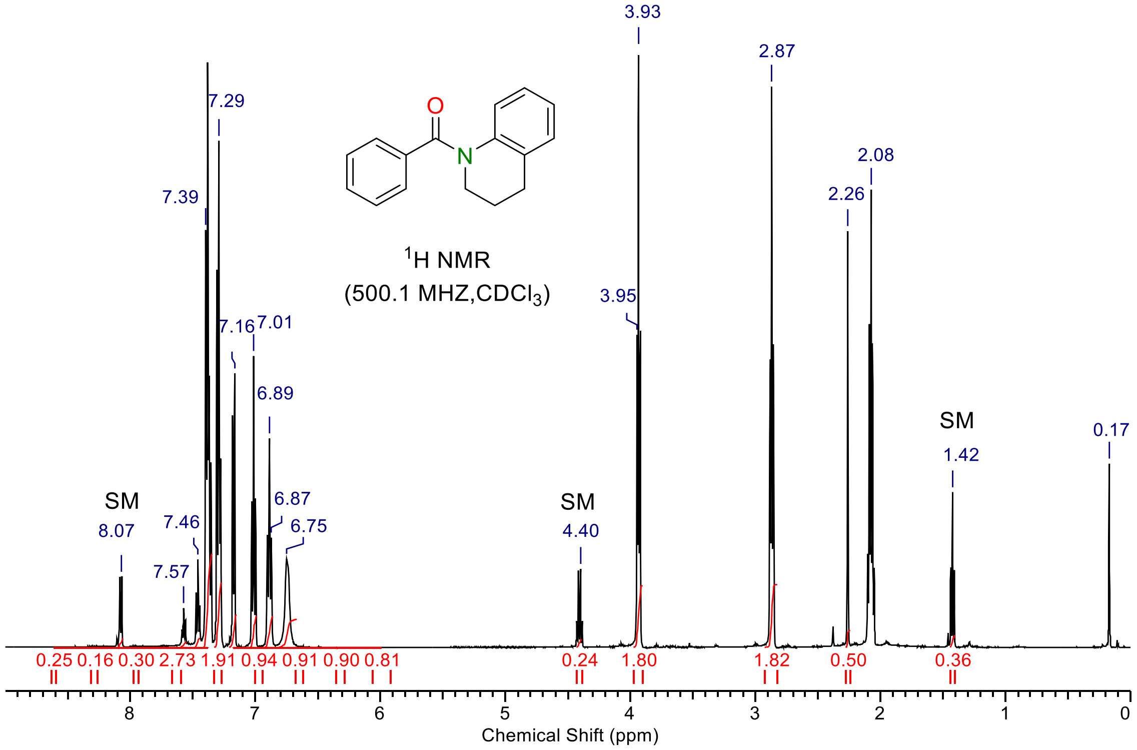

Spectra S21: ¹H NMR spectrum of synthesised 4r with Hexamethylbenzene as an internal standard

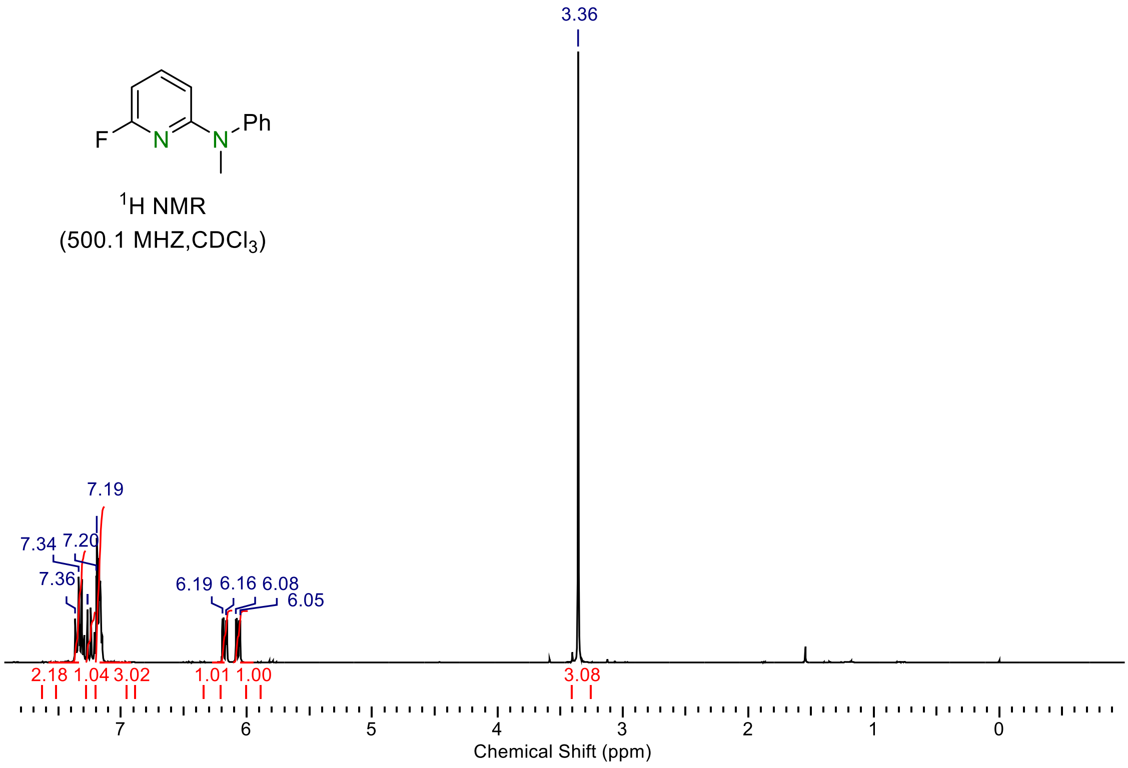

Spectra S22: ¹H NMR spectrum of isolated 5a

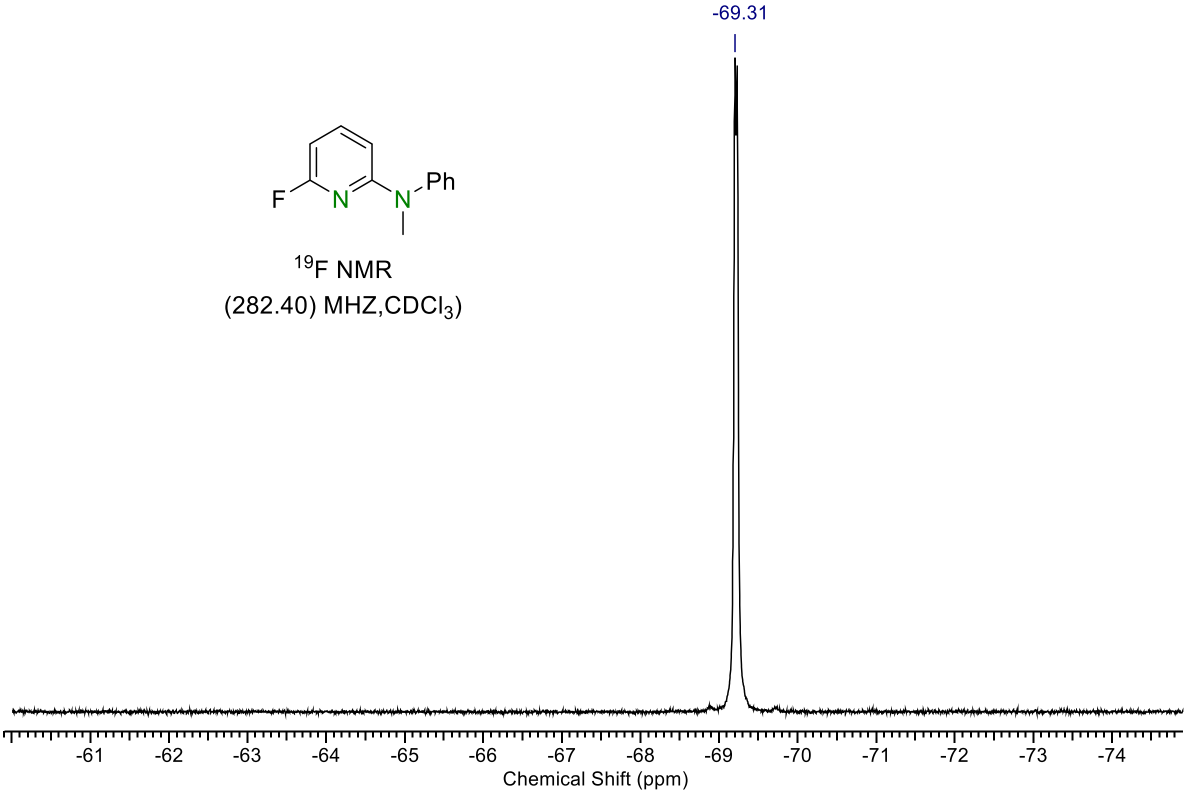

Spectra S23: ^{19}F NMR spectrum of isolated 5a

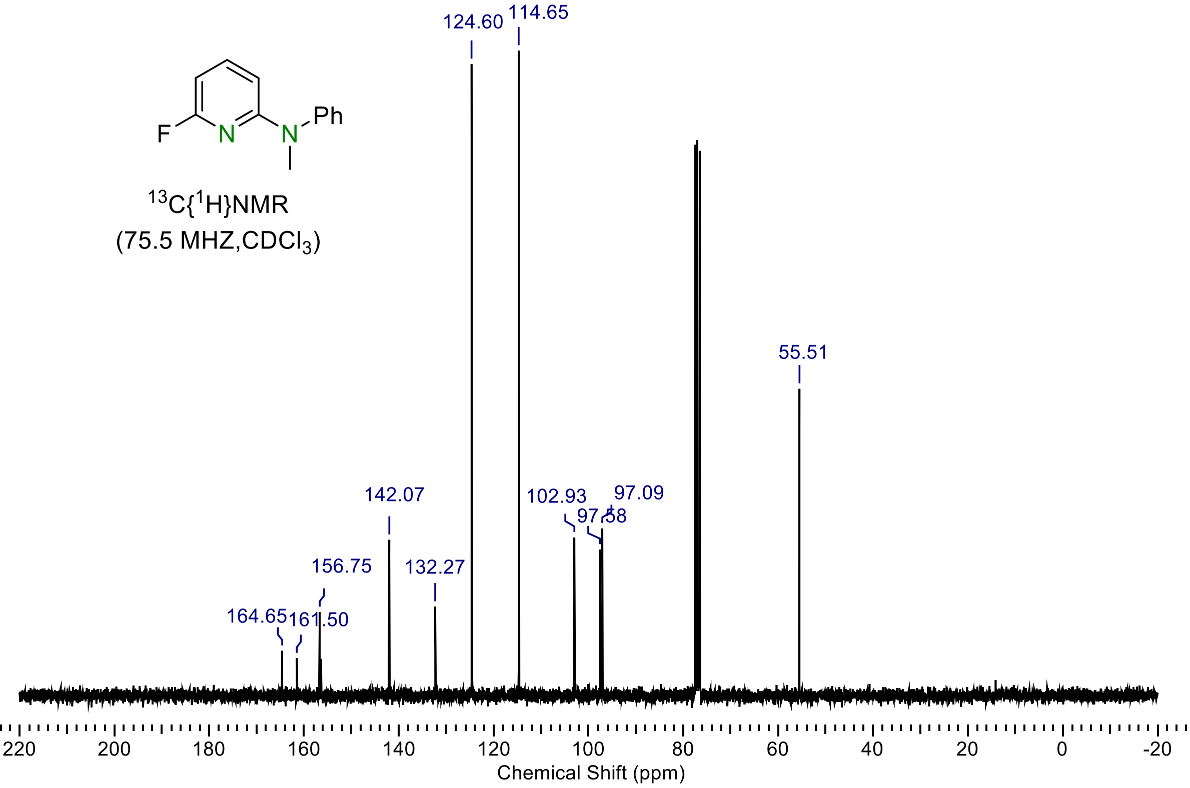

Spectra S24: ^{13}C(^{1}H) NMR spectrum of isolated 5a

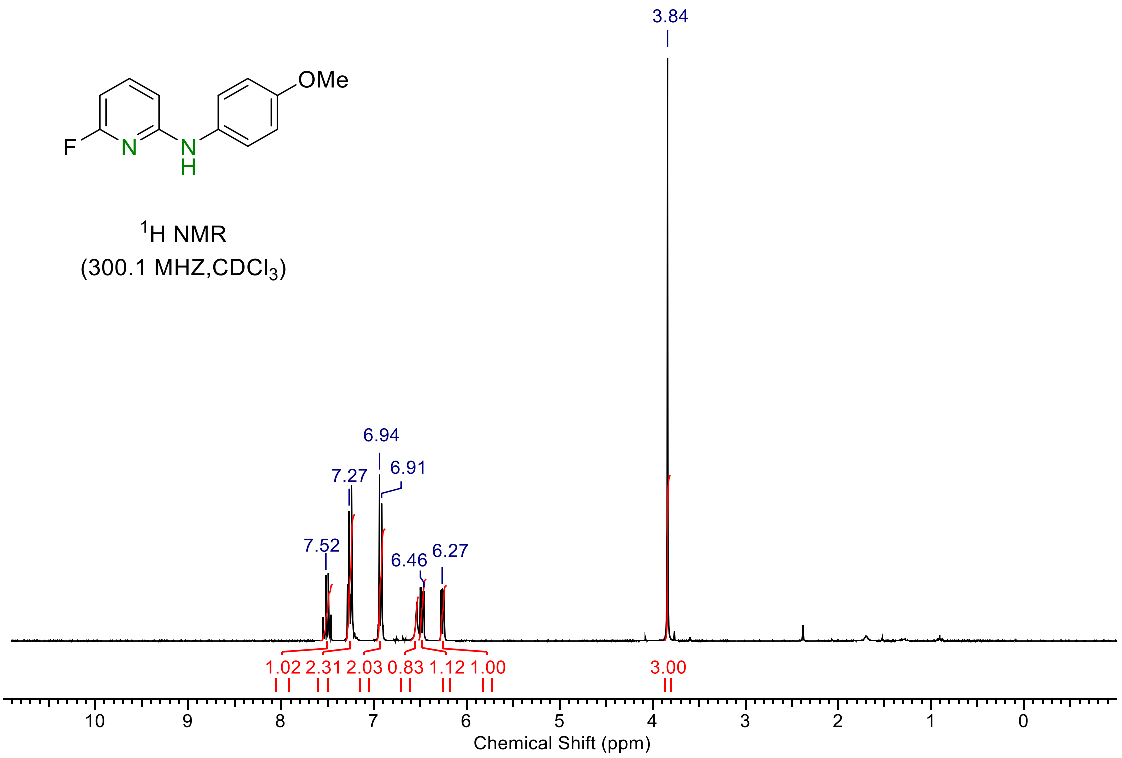

Spectra S25: ^{1}H NMR spectrum of isolated 5b

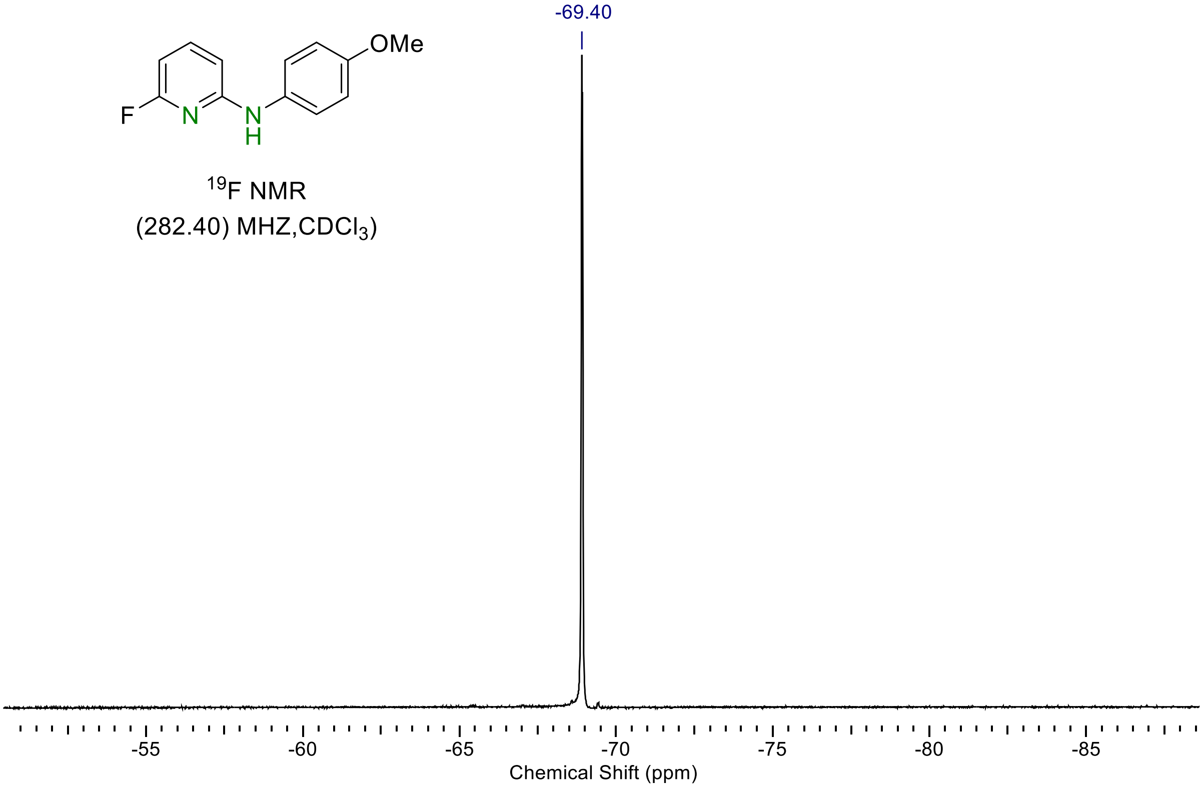

Spectra S26: ^{19}F NMR spectrum of isolated 5b

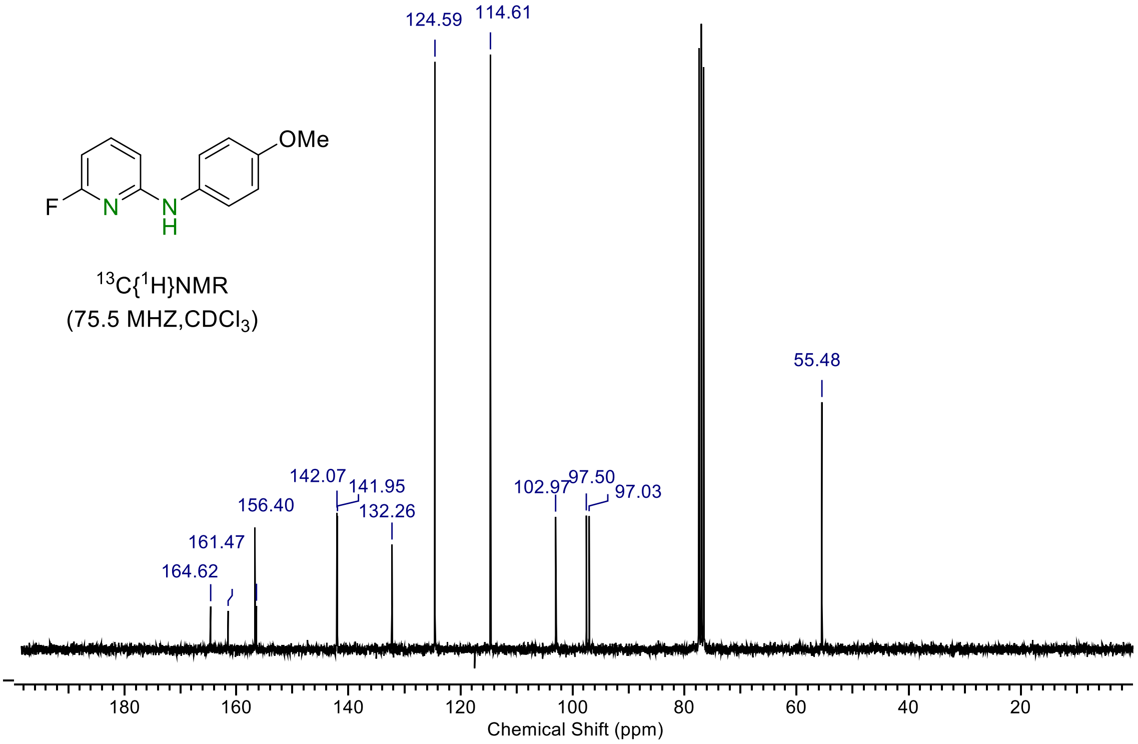

Spectra S27: $^{13}C(^1H)$ NMR spectrum of isolated 5b

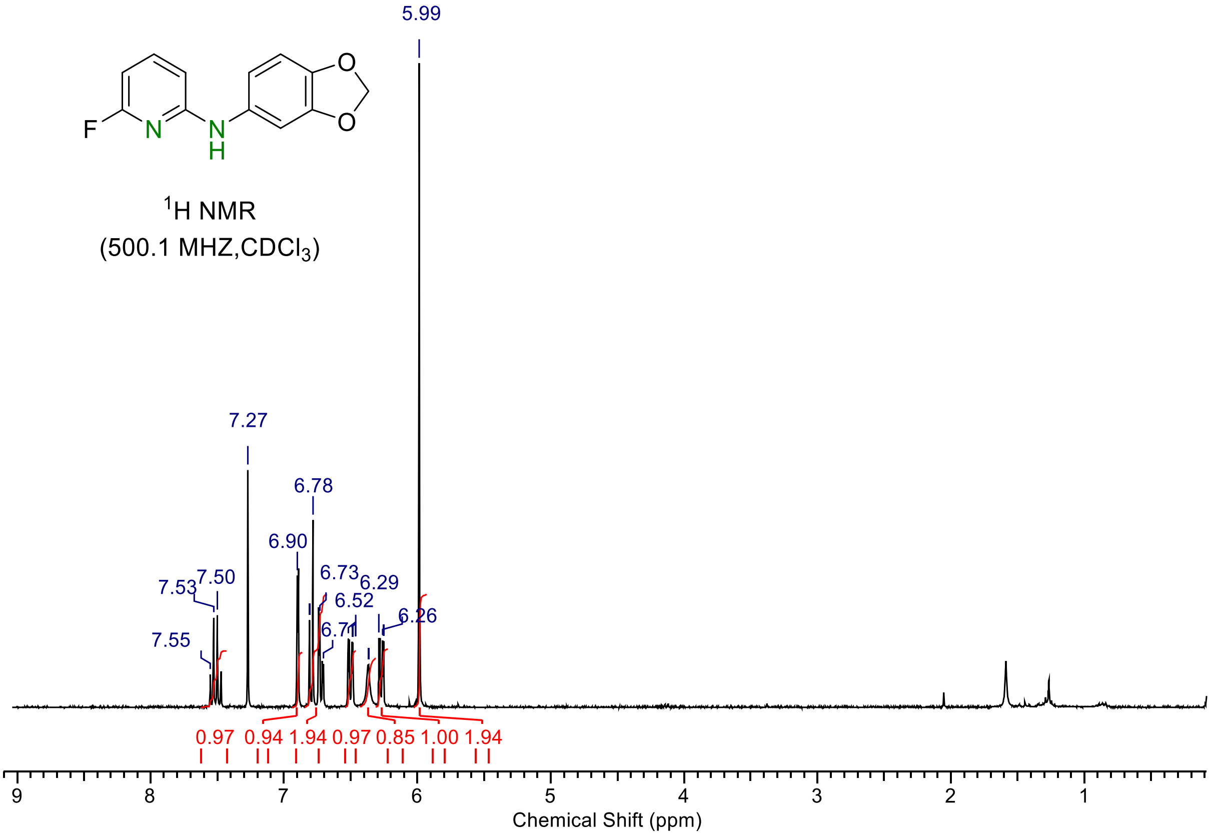

Spectra S28: ^1H NMR spectrum of isolated 5c

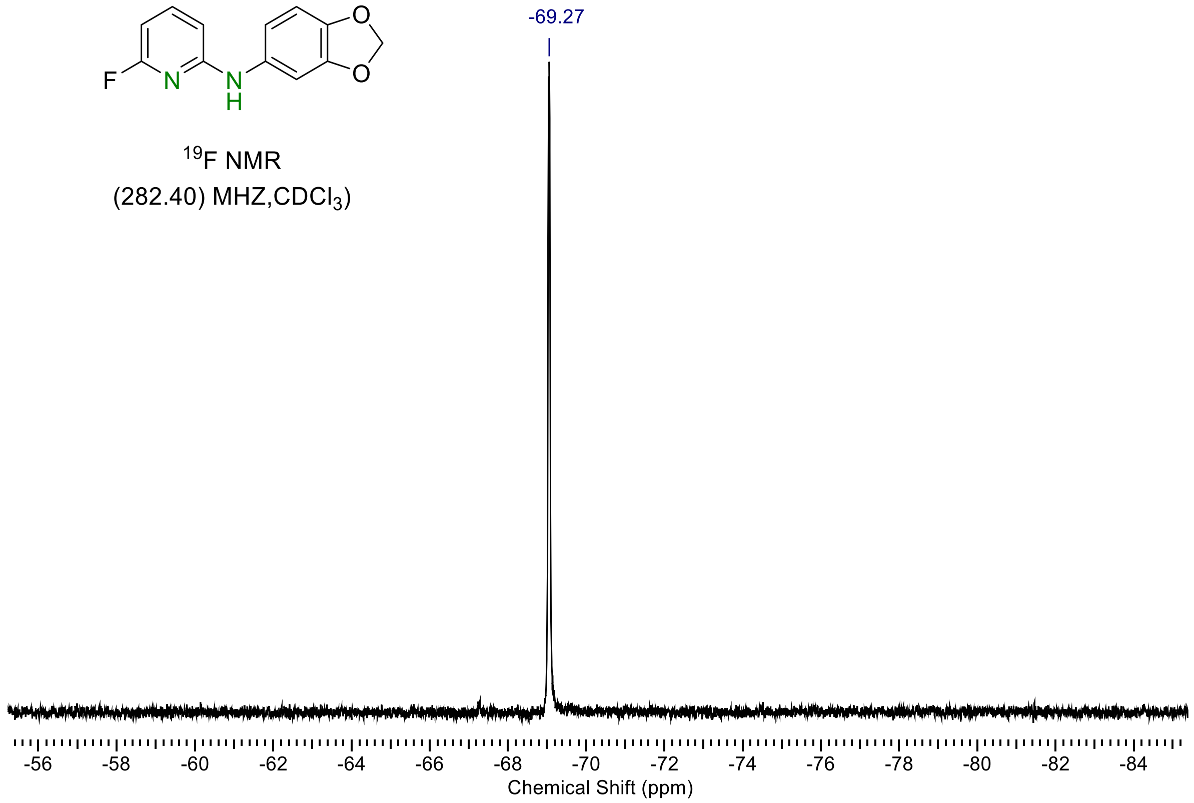

Spectra S29: ¹⁹ F NMR spectrum of isolated 5c

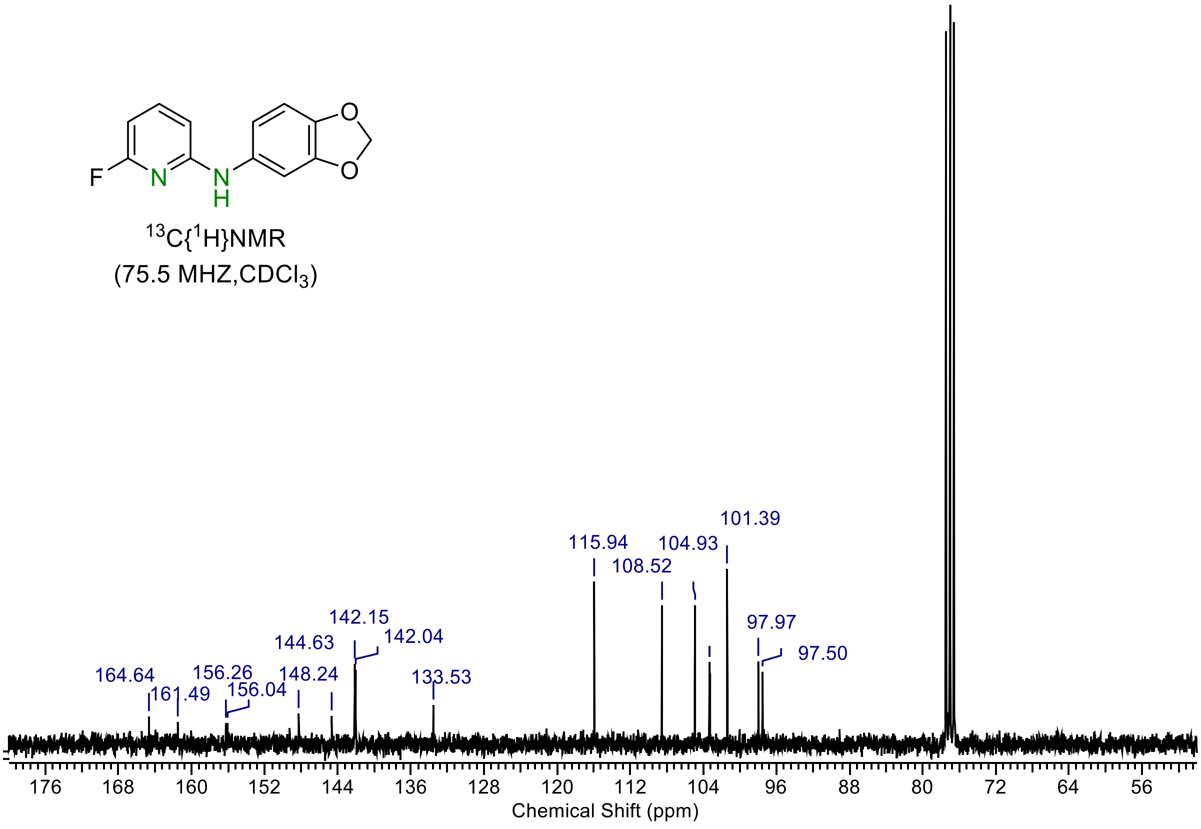

Spectra S30: $^{13}C(^{1}H)$ NMR spectrum of isolated 5c

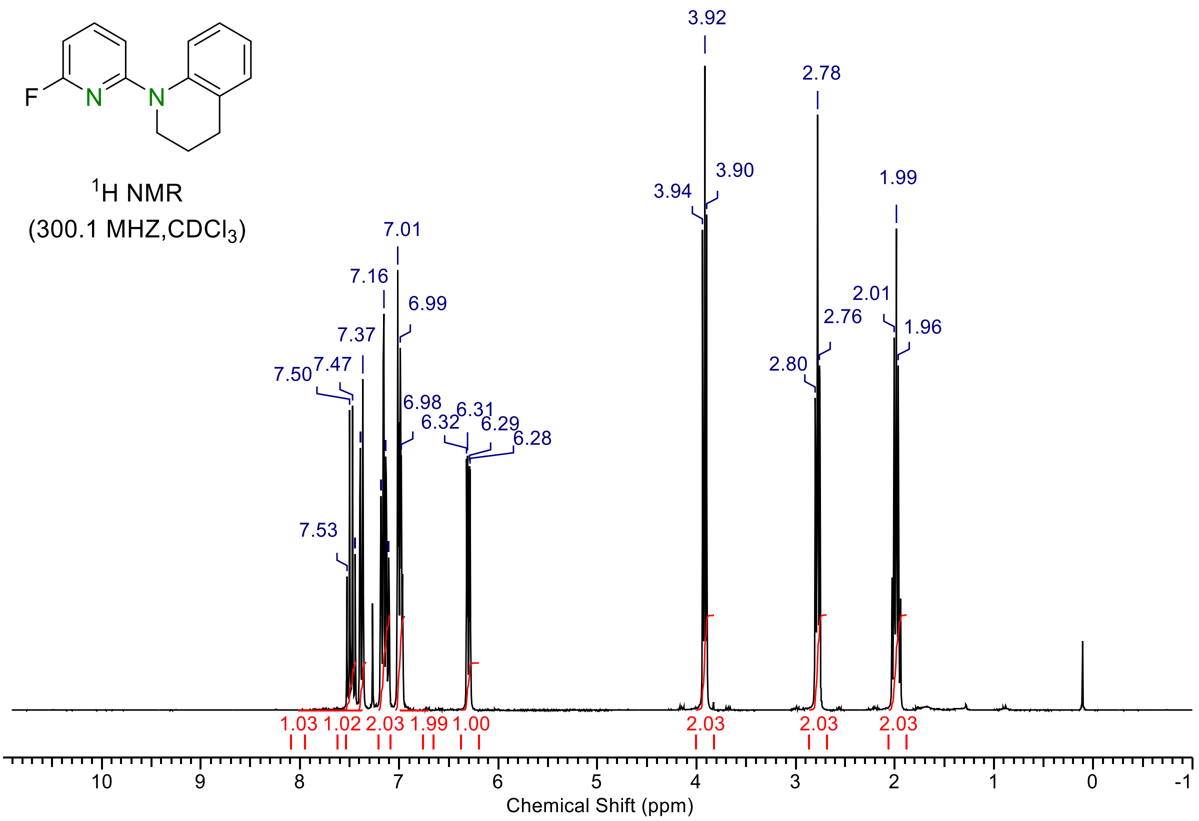

Spectra S31: ^{1}H NMR spectrum of isolated 5d

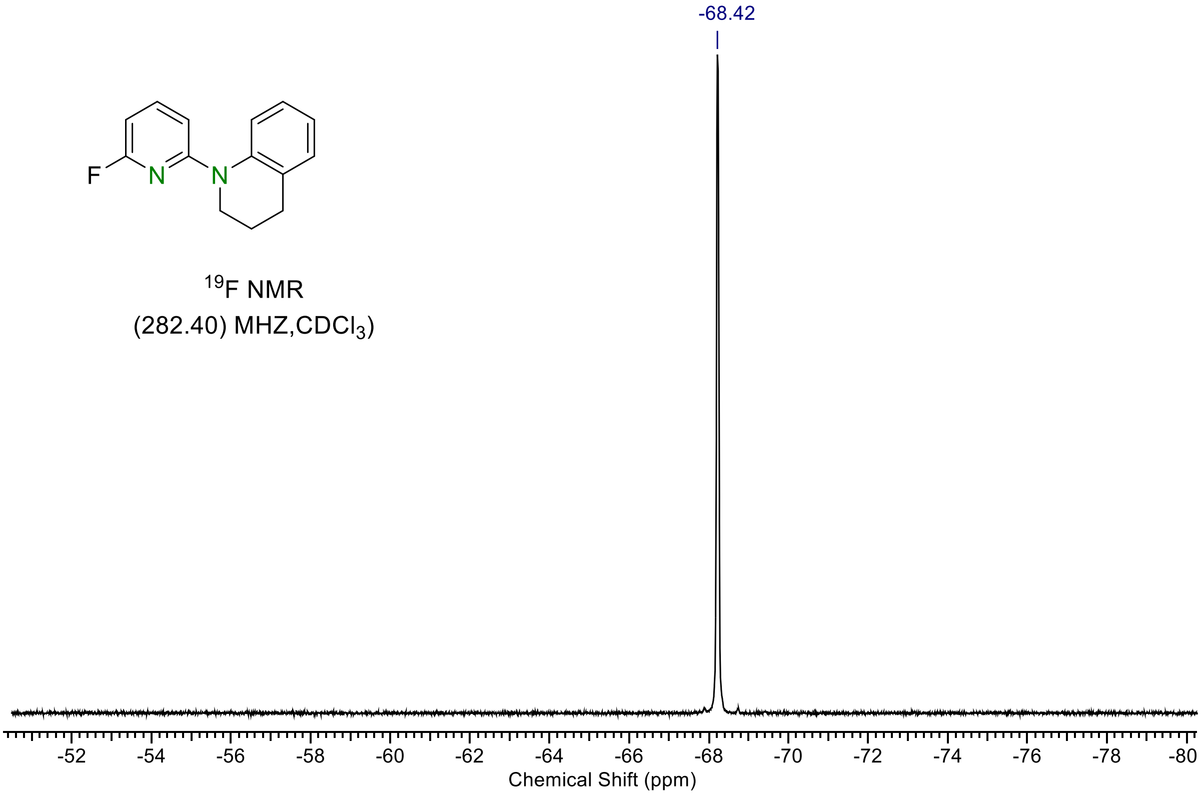

Spectra S32: ^{19}F NMR spectrum of isolated 5d

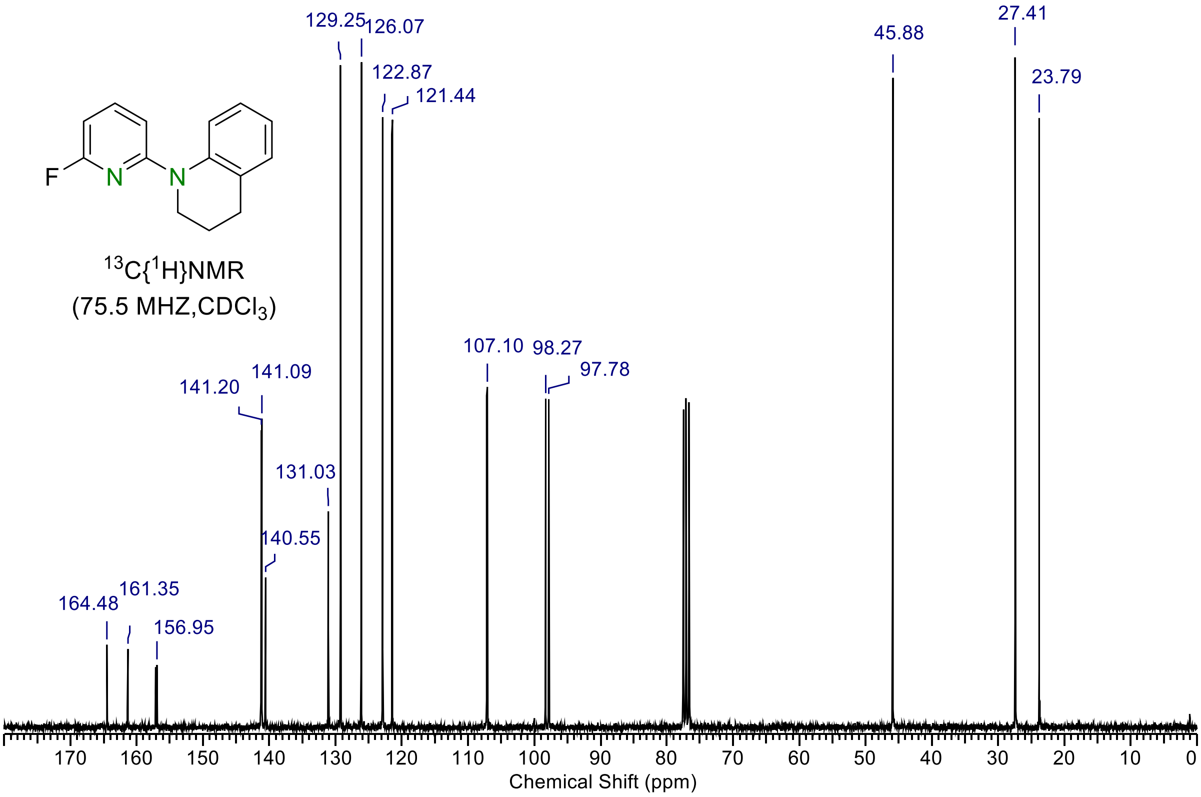

Spectra S33: $^{13}C(^1H)$ NMR spectrum of isolated 5d

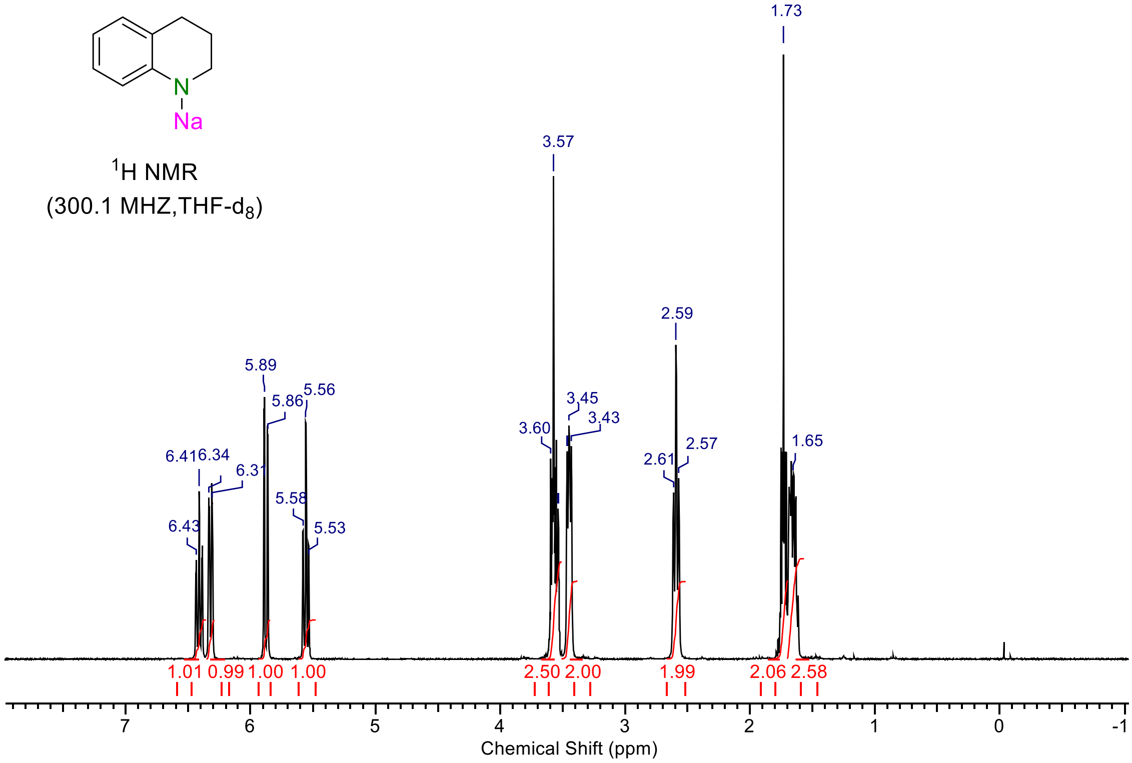

Spectra S34: ^1H NMR spectrum of isolated 6

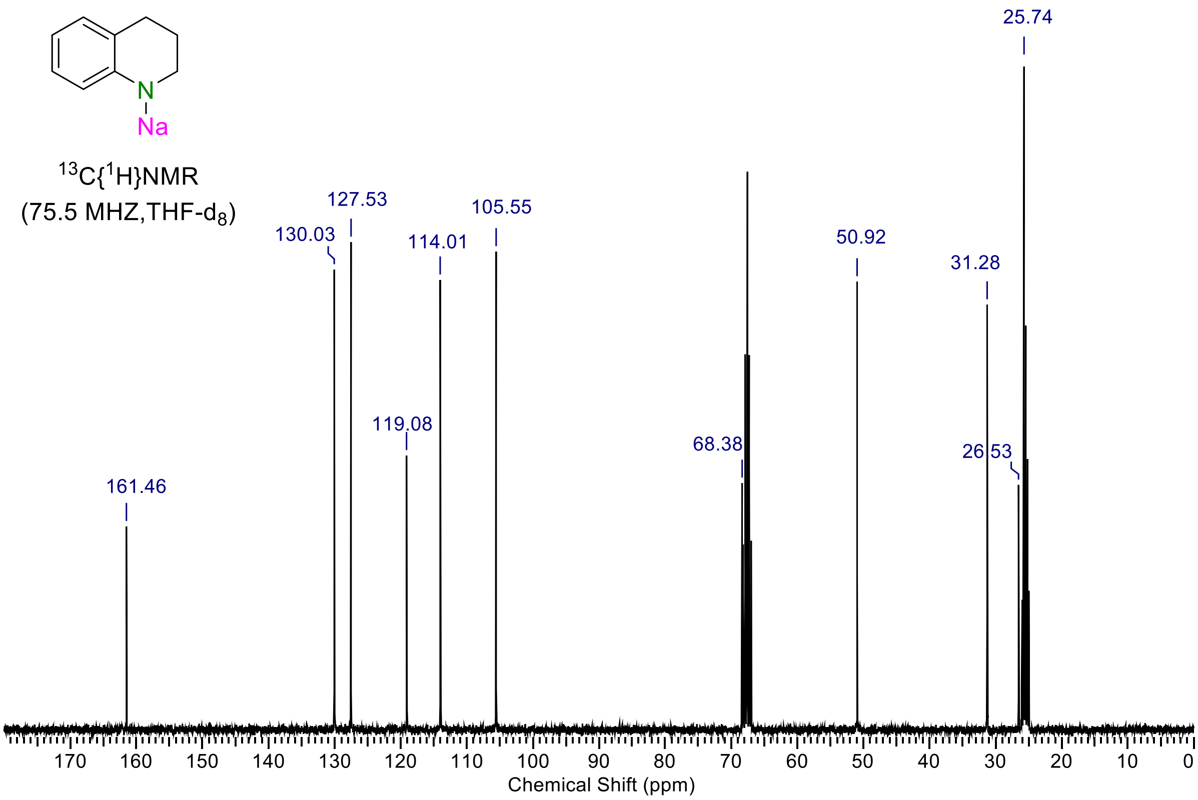

Spectra S35: $^{13}C(^{1}H)$ NMR spectrum of isolated 6

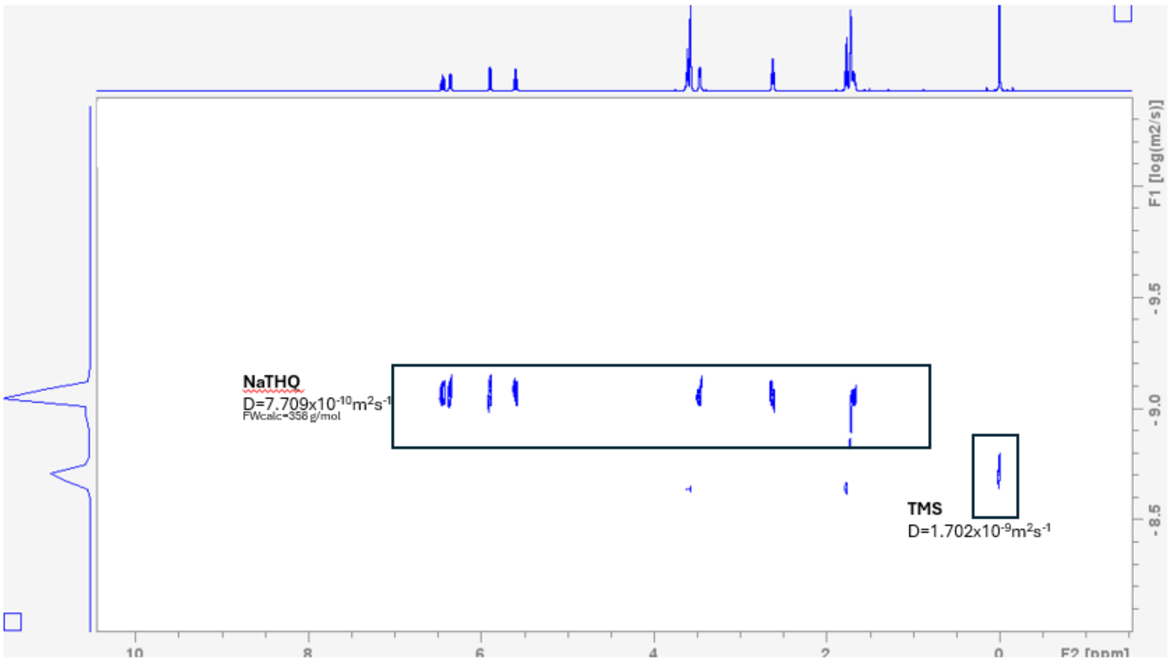

Entry	Aggregate	FW	ΔFW
1	NaTHQ(THF)$_2$	299	-16%
2	NaTHQ(THF)$_3$	371	4%
4	(NaTHQ)$_2$(THF)$_2$	454	27%

Spectra S36: ^{1}H DOSY NMR spectrum of isolated 6 in THF-d_8 (20 mM), potential aggregates listed above

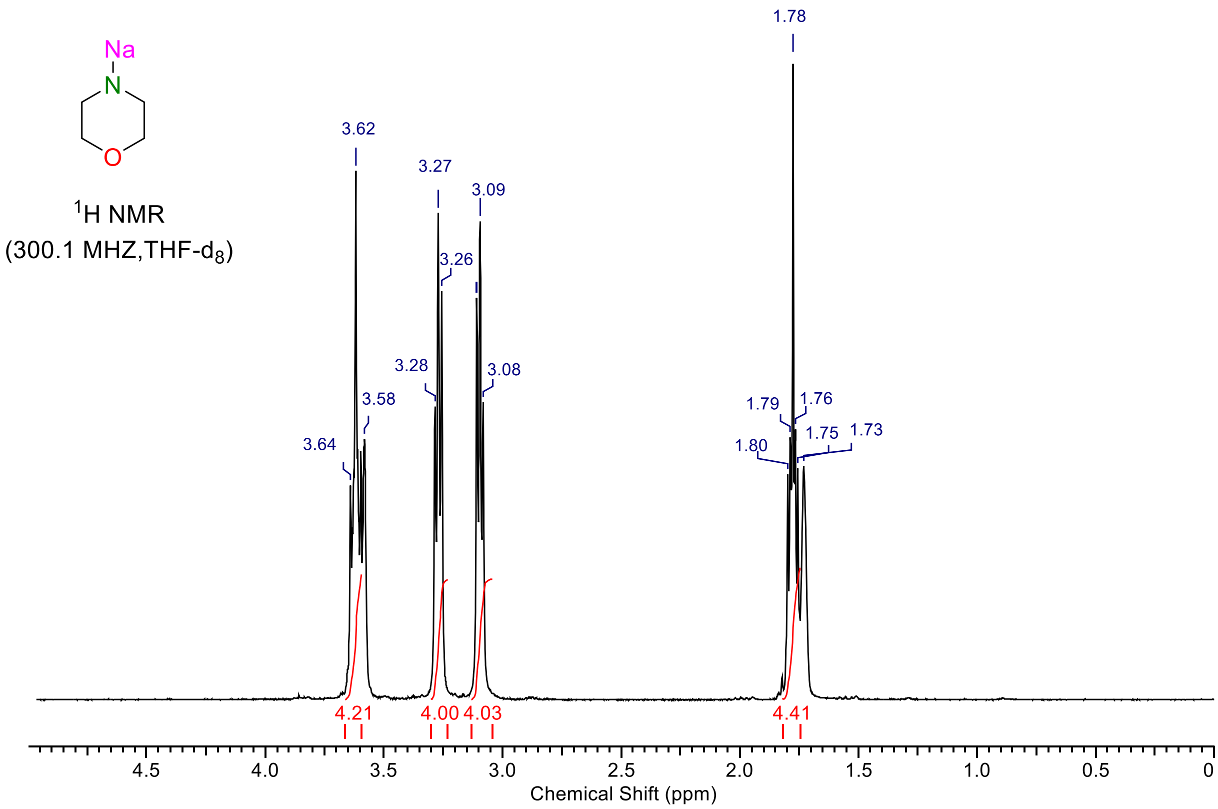

Spectra S37: ^{1}H NMR spectrum of isolated 7

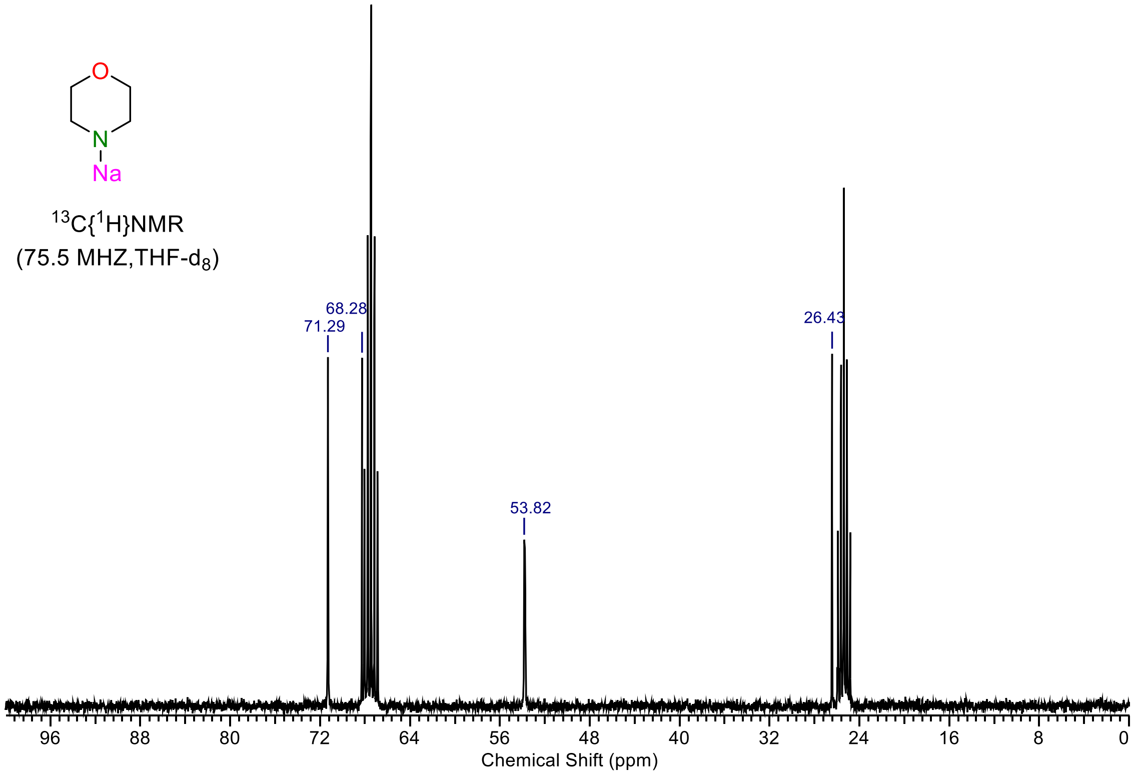

Spectra S38: ^{13}C(^{1}H) NMR spectrum of isolated 7

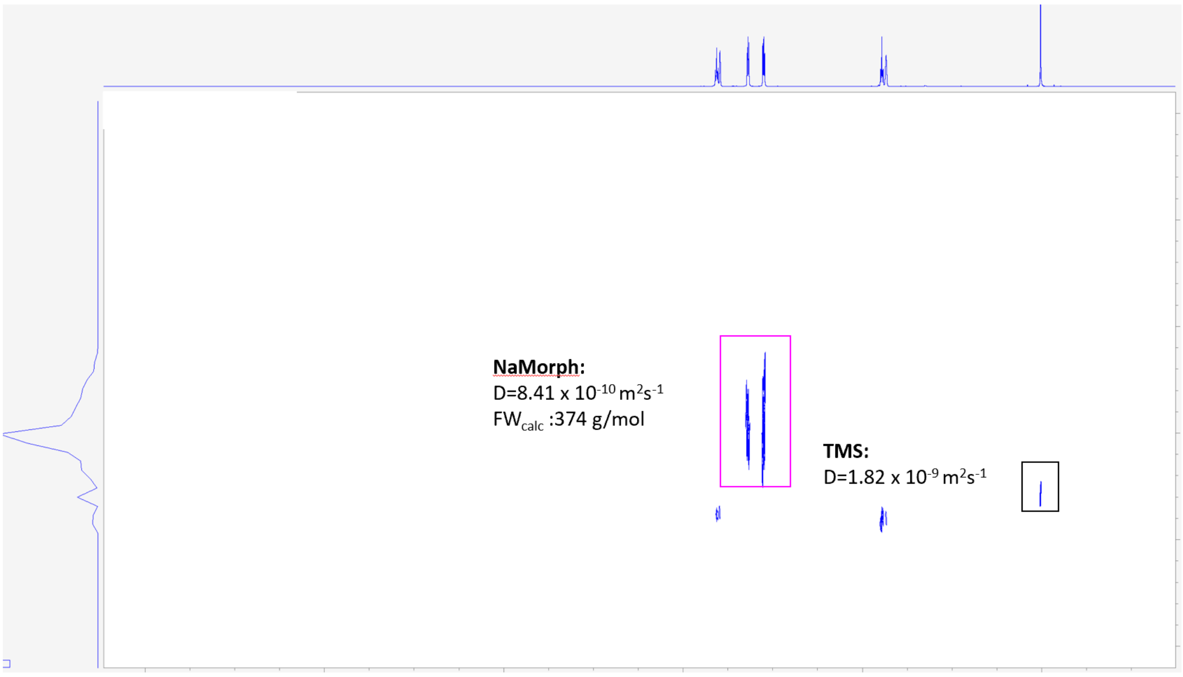

With FW det = 374 g/mol (DSE), two viable aggregations are possible either a dimeric structure entry 4 or a tetrasolvated structure entry 3.

Entry	Aggregate	FW	ΔFW
1	$NaMorph(THF)_2$	253	-32%
2	$NaMorph(THF)_3$	325	-13%
3	$NaMorph(THF)_4$	397	6%
4	$(NaMorph)_2(THF)_2$	362	-3%

Spectra S39: ^{1}H DOSY NMR spectrum of isolated 7 in THF-d_8 (20 mM) potential aggregates listed above.

X-Ray Crystallography

The crystal structures of all novel compounds have been deposited into the Cambridge Crystallographic Data Centre (CCDC) and have been assigned the following numbers: **6-2414322; 7- 2414321 s**elected crystallographic and refinement parameters are presented below (**Table S14**). In the case of 2414321 the crystals were immersed in an inert parabar oil were mounted at low temperature using the X-TEMP2[11,12] cold temperature device and transferred into the nitrogen stream at 173 K. 2414322 crystals were mounted at ambient temperature. All measurements were made on a RIGAKU Synergy S area-detector diffractometer[13] using mirror optics monochromated Cu Kα radiation (λ = 1.54184 Å) or on an Oxford Diffraction SuperNova area-detector diffractometer using mirror optics monochromated Mo Kα radiation (λ = 0.71073 Å) and Al filtered. Data reduction was performed using the CrysAlisPro program.[13] The intensities were corrected for Lorentz and polarization effects, and an absorption correction based on the Gaussian method using SCALE3 ABSPACK in CrysAlisPro was applied. The structure was solved by direct methods or intrinsic phasing usingSHELXT,[14] which revealed the positions of all non-hydrogen atoms of the compounds. All non-hydrogen atoms were refined anisotropically. H-atoms were assigned in geometricallycalculated positions and refined using a riding model where each H-atom was assigned a fixed S40isotropic displacement parameter with a value equal to 1.2Ueq of its parent atom (1.5Ueq for methyl groups). Refinement of the structure was carried out on F 2 using full-matrix least- squares procedures, which minimized the function $\Sigma w(Fo^2 – Fc^2)^2$. The weighting scheme was based on counting statistics and included a factor to downweight the intense reflections. Allcalculations were performed using the SHELXL-2014/718 program in OLEX2[15].

Compound	**6**	**7**
Empirical formula	$C_{34}H_{52}N_2Na_2O_4$	$C_8H_{16}NNaO_2$
Formula weight	598.75	181.21
Temperature/K	173.00(10)	173.00(10)
Crystal system	monoclinic	orthorhombic
Space group	$P2_1/c$	Pbca
a/Å	8.54477(17)	12.23749(9)

b/Å	15.8450(3)	10.14149(8)
c/Å	12.8194(2)	16.48477(16)
α/°	90	90
β/°	100.7336(19)	90
γ/°	90	90
Volume/Å^3	1705.28(6)	2045.87(3)
Z	2	8
ρ_{calc}g/cm^3	1.166	1.177
μ/mm^{-1}	0.097	1.037
F(000)	648.0	784.0
Crystal size/mm^3	0.219 × 0.188 × 0.157	0.372 × 0.247 × 0.176
Radiation	Mo Kα (λ = 0.71073)	Cu Kα (λ = 1.54184)
2Θ range for data collection/°	4.132 to 61.012	10.734 to 148.994
Index ranges	-12 ≤ h ≤ 12, -22 ≤ k ≤ 22, -18 ≤ l ≤ 18	-14 ≤ h ≤ 15, -10 ≤ k ≤ 12, -15 ≤ l ≤ 20
Reflections collected	111679	20220
Independent reflections	5193 [R_{int} = 0.0350, R_{sigma} = 0.0128]	2091 [R_{int} = 0.0209, R_{sigma} = 0.0109]
Data/restraints/parameters	5193/14/219	2091/17/129
Goodness-of-fit on F^2	1.046	1.083
Final R indexes [I>=2σ (I)]	R_1 = 0.0461, wR_2 = 0.1276	R_1 = 0.0342, wR_2 = 0.1032
Final R indexes [all data]	R_1 = 0.0613, wR_2 = 0.1368	R_1 = 0.0354, wR_2 = 0.1043
Largest diff. peak/hole / e Å^{-3}	0.34/-0.15	0.23/-0.24

Table S14: Crystal data and structure refinement details for compounds 6 and 7

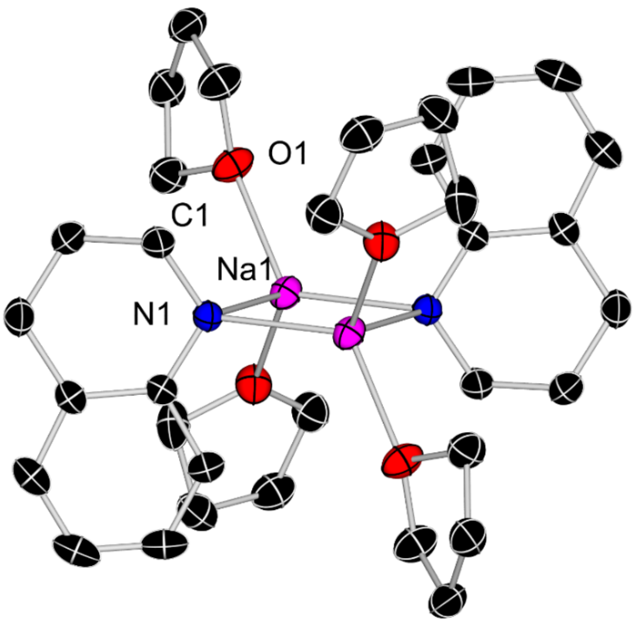

Figure S8 Solid-state structure of 6. Thermal ellipsoids shown at 30 % probability and hydrogen atoms. Selected bond lengths [Å]: Na1-O1 2.3571(10) Na1 O2 2.3110(10) Na1-N1 2.3783(10) N1-C1 1.4571(15)

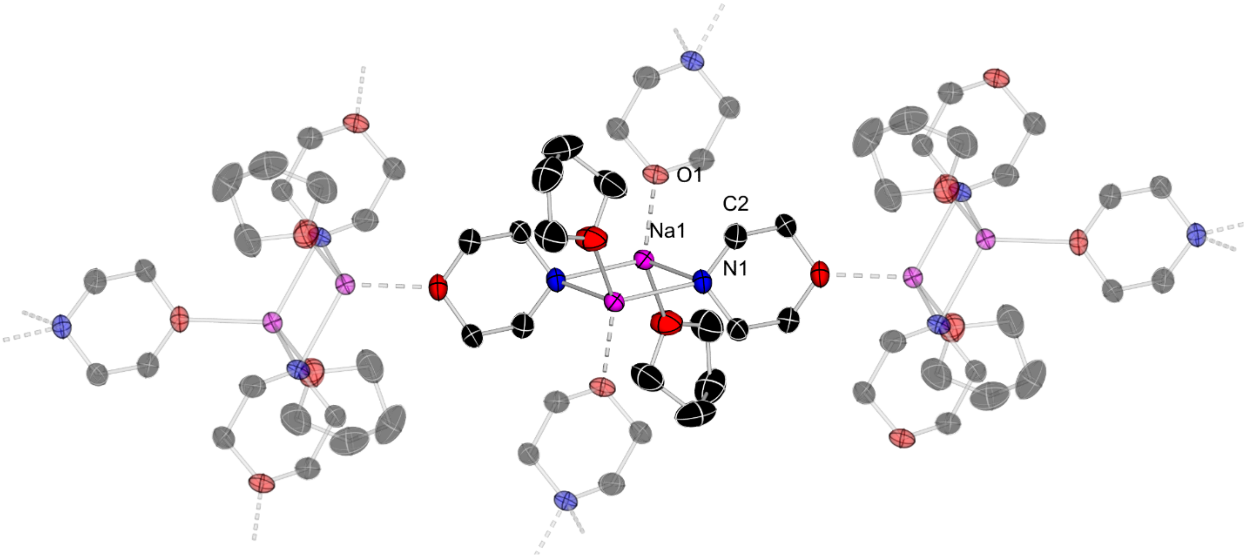

Figure S9 solid-state structure of 7. Thermal ellipsoids shown at 30 % probability and hydrogen atoms. Selected bond lengths [Å]: Na1-O1 2.315(8), Na1-O2 2.3614(10),Na-N1 2.3880(11)N1 C2 1.4424(15)

All structures were measured and solved by the X-ray crystal structure determination service unit of the Department of Chemistry, Biochemistry and Pharmaceutical Sciences of the University of Bern is acknowledged for measuring, solving, refining and summarizing the structures of compounds **6** and **7.** The Synergy diffractometer was partially funded by the Swiss National Science Foundation (SNF) within the R'Equip programme (project number 206021_177033).

References

(1) Borys, A. M. *The Schlenk Line Survival Guide*. https://schlenklinesurvivalguide.com.

(2) Fairley, M.; Bole, L. J.; Mulks, F. F.; Main, L.; Kennedy, A. R.; O'Hara, C. T.; García-Alvarez, J.; Hevia, E. Ultrafast Amidation of Esters Using Lithium Amides under Aerobic Ambient Temperature Conditions in Sustainable Solvents. *Chem. Sci.* **2020**, *11* (25), 6500–6509. https://doi.org/10.1039/D0SC01349H.

(3) Liu, C.; Chen, H.-N.; Xiao, T.-F.; Hu, X.-Q.; Xu, P.-F.; Xu, G.-Q. Organic Photoredox Catalyzed Dealkylation/Acylation of Tertiary Amines to Access Amides. *Chem. Commun.* **2023**, *59* (14), 2003–2006. https://doi.org/10.1039/D2CC05842A.

(4) Li, L.; Wang, X.; Fu, N. Electrochemical Nickel-Catalyzed Hydrogenation. *Angewandte Chemie International Edition* **2024**, *63* (22), e202403475. https://doi.org/10.1002/anie.202403475.

(5) Lian, Q.; Chen, J.; Huang, K.; Hou, K.; Fang, J.; Wei, W.; Zhou, J. Alkali-Driven Photoinduced N-Dealkylation of Aryl Tertiary Amines and Amides. *Org. Lett.* **2023**, *25* (46), 8387–8392. https://doi.org/10.1021/acs.orglett.3c03519.

(6) Urgoitia, G.; Obieta, M.; Herrero, M. T.; Lezama, L.; SanMartin, R. Molecular Oxygen-Induced Transamidation of Unactivated Amides in Diethyl Carbonate in the Presence of a Palladium Catalyst. *Advanced Synthesis & Catalysis* **2023**, *365* (24), 4713–4725. https://doi.org/10.1002/adsc.202300984.

(7) Li, G.; Ji, C.-L.; Hong, X.; Szostak, M. Highly Chemoselective, Transition-Metal-Free Transamidation of Unactivated Amides and Direct Amidation of Alkyl Esters by N–C/O–C Cleavage. *J. Am. Chem. Soc.* **2019**, *141* (28), 11161–11172. https://doi.org/10.1021/jacs.9b04136.

(8) Deng, X.; Jiang, F.; Wang, X. Asymmetric Deoxygenative Functionalization of Secondary Amides with Vinylpyridines Enabled by a Triple Iridium-Photoredox-Chiral Phosphoric Acid System. *Org. Lett.* **2024**, *26* (12), 2483–2488. https://doi.org/10.1021/acs.orglett.4c00692.

(9) Rao, W.-H.; Li, Y.-G.; Jiang, L.-L.; Li, Q.; Zou, G.-D.; Cao, X. Metal-Free Selective Ortho-C–H Amidation of Hypervalent(III) Iodobezenes with N-Methoxy Amides under Mild Conditions. *J. Org. Chem.* **2023**, *88* (19), 13825–13837. https://doi.org/10.1021/acs.joc.3c01472.

(10) Lebedev, A. N.; Rodygin, K. S.; Vakhrusheva, S. A.; Ananikov, V. P. A 60-Times Faster Digital-Discovery-Compatible Reaction Setup with Enhanced Safety for Chemical Applications. *Green Chem.* **2024**, *26* (7), 3776–3785. https://doi.org/10.1039/D3GC04064J.

(11) Kottke, T.; Stalke, D. Crystal Handling at Low Temperatures. *J Appl Cryst* **1993**, *26* (4), 615–619. https://doi.org/10.1107/S0021889893002018.
(12) Stalke, D. Cryo Crystal Structure Determination and Application to Intermediates. *Chemical Society Reviews* **1998**, *27* (3), 171–178. https://doi.org/10.1039/A827171Z.
(13) Oxford Diffraction (2018). CrysAlisPro (Version 1.171.40.37a). Oxford Diffraction Ltd., Yarnton, Oxfordshire, UK. *Oxford Diffraction (2018). CrysAlisPro (Version 1.171.40.37a). Oxford Diffraction Ltd., Yarnton, Oxfordshire, UK.*
(14) Sheldrick, G. M. SHELXT – Integrated Space-Group and Crystal-Structure Determination. *Acta Cryst A* **2015**, *71* (1), 3–8. https://doi.org/10.1107/S2053273314026370.
(15) Dolomanov, O. V.; Bourhis, L. J.; Gildea, R. J.; Howard, J. a. K.; Puschmann, H. OLEX2: A Complete Structure Solution, Refinement and Analysis Program. *J Appl Cryst* **2009**, *42* (2), 339–341. https://doi.org/10.1107/S0021889808042726.
